# Total Synthesis of (±)-Paroxetine by Diastereoconvergent Cobalt-Catalysed Arylation

**DOI:** 10.1002/ejoc.201402108

**Published:** 2014-05-27

**Authors:** Carole F Despiau, Andrew P Dominey, David C Harrowven, Bruno Linclau

**Affiliations:** a Chemistry, University of Southampton, Highfield, Southampton SO17 1BJ, UK http://www.southampton.ac.uk/chemistry/about/staff/linclau.page; b GlaxoSmithKline, Medicines Research Centre Gunnels Wood Road, Stevenage SG1 2NY, UK

**Keywords:** Total synthesis, Homogeneous catalysis, Nitrogen heterocycles, Cross-coupling, Cobalt, Radicals

## Abstract

A total synthesis of paroxetine is reported, with a diastereoselective and diastereoconvergent cobalt-catalysed sp^3^–sp^2^ coupling reaction involving a 3-substituted 4-bromo-*N*-Boc-piperidine (Boc = *tert*-butoxycarbonyl) substrate as a key step. A 9:1 diastereoselectivity was obtained, while a control experiment involving a conformationally locked 3-substituted 4-bromo-*tert*-butyl cyclohexane ring proceeded with essentially complete stereoselectivity.

## Introduction

Recent years have seen a marked rise in the use of cheaper transition metals for catalytic C–C bond formation. Iron and cobalt are particularly attractive for large-scale metal catalysis as they have far lower toxicities than nickel or palladium. Recent demonstrations of their ability to catalyse the union of aryl Grignard reagents with unactivated secondary alkyl halides represent a significant advance in the synthetic potential of these emerging protocols.^1^,^2^

**Scheme 1:**
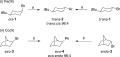
Diastereoconvergent coupling reactions;3a,3b a) FeCl_3_ (5 mol-%), ArMgBr (1.3 equiv.), TMEDA (1.2 equiv.), THF, room temp., 30 min; b) CoCl_2_ (5 mol-%), PhMgBr (1.2 equiv.), (*R*,*R*)-*N*,*N*,*N′*,*N′*-tetramethyl-1,2-cyclohexanediamine (6 mol-%), THF, 25 °C, 15 min.

To date, investigations into the diastereoselectivity of such cross-couplings are limited.^3^ With 3- and 4-substituted bromocyclohexane derivatives, a number of reports have demonstrated the preferential incorporation of the “nucleophilic” component into the less encumbered equatorial position in iron-catalysed cross-coupling reactions [e.g., Scheme 1 (i)].3a Similar observations have been made for the analogous cobalt-catalysed reactions, with bicycloheptanes *exo*-**3** and *endo*-**3** both giving *exo*-**4** when treated with PhMgBr [Scheme 1 (ii)].3b

Mechanistically, the stereochemical outcome has been explained by the involvement of a radical intermediate, as seen in the proposed mechanism shown in Scheme 2.3c,^4^ Ferrate complexes (**I**) have been proposed as the reactive species when the Grignard reagent involved is unable to undergo β-hydride elimination. Indeed, such complexes have been prepared by Fürstner et al., who showed that they efficiently catalyse cross-coupling reactions.^5^ An alkyl radical (**II**) can then be generated by reaction of the reduced ferrate complex with the alkyl bromide.^6^ Recombination of the alkyl radical with the metal complex to give **III**, followed by reductive elimination, would liberate the cross-coupling product. Similar mechanisms have been proposed for the cobalt-catalysed allylation of alkyl halides,3b,^7^ as well as for a cobalt-catalysed tandem cyclisation and arylation reaction.^8^ The configurational lability of the radical intermediate (**II**) nicely accounts for the formation of the most stable diastereoisomer (e.g., *trans*-**2**, *exo***-4**). A late transition state for the reductive elimination step has also been invoked to explain the bias towards production of the thermodynamic product.^1^,^3^

**Scheme 2:**
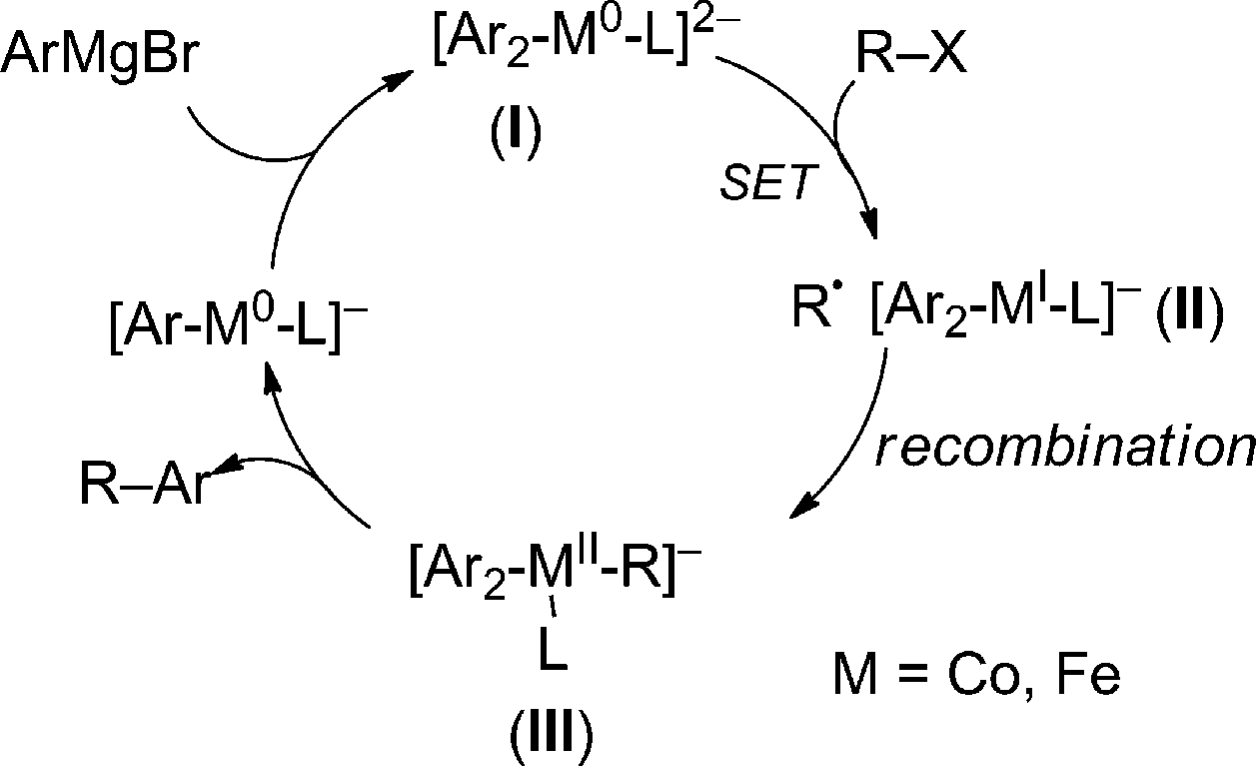
Proposed mechanism for the cross-coupling reaction.^3^–^8^ SET = single-electron transfer.

**Scheme 3:**
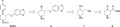
Retrosynthetic analysis. Boc = *tert*-butoxycarbonyl.

In this paper, we report a successful application of this diastereoconvergent cross-coupling methodology in a short synthesis of paroxetine **5** (Paxil®, Scheme 3).^9^ Paroxetine is a potent inhibitor of serotonin reuptake, and is widely prescribed for the treatment of depression, obsessive-compulsive disorder, panic disorder, social and anxiety disorder, and post-traumatic stress disorder.^10^

## Results and Discussion

Our retrosynthetic analysis is shown in Scheme 3, with secondary bromide **6** as a key intermediate for the introduction of the aryl residue. As the coupling is expected to be diastereoconvergent, our strategy allows for its production as a mixture of diastereomers. Consequently, the synthesis of **6** can be envisioned from commercially available **8** using standard chemistry.

A literature survey revealed that *N*-protected 4-bromopiperidine derivatives have been used as substrates in iron/cobalt-catalysed coupling reactions,^11^ yet none of the examples reported featured α-alkyl substitution. Moreover, cross-couplings on six-membered rings with α-alkyl substituents have little precedent.^12^,^13^ Hence, both the reactivity and the stereochemical outcome of our key step would be instructive, given that substituted piperidines are ubiquitous in natural products and medicines.9j,^14^,^15^

**Scheme 4:**
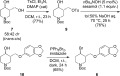
Synthesis of the cross-coupling precursor; DMAP = 4-(dimethylamino)pyridine.

The synthesis of bromide precursor **6** was readily accomplished in three steps from known diol **7** (Scheme 4).^16^ Regioselective tosylation of **7** (*dr* 58:42) proceeded in good yield using triethylamine (2.1 equiv.) as the base. Introduction of the sesamol group with Cs_2_CO_3_ in DMF gave adduct **10** in 51 % yield. This yield improved to 76 % when a toluene solution of **9** and sesamol was exposed to aqueous NaOH using tetrabutylammonium hydroxide as phase-transfer catalyst. Finally, conversion of alcohol **10** into bromide **6** was achieved in 65 % yield through the action of bromotriphenylphosphonium bromide. Bromination of the electron-rich sesamol ring was never observed with this reagent, in contrast to related procedures using triphenylphosphine and bromine, where it proved to be a minor side-reaction (10 % yield).

We were now in a position to examine our key cross-coupling step with *p*-fluorophenylmagnesium bromide. For completeness, we decided to separate the diastereoisomers of **6** in order to rigorously establish diastereoconvergence for each stereoisomer. To that end, the *cis* and *trans* diastereomers of alcohol **10** were separated by chromatography, then each was brominated to give *trans*- and *cis*-**6**, respectively. However, it proved more convenient to separate *cis* and *trans*bromides **6** by selective precipitation from hexane/Et_2_O.

The optimisation studies for the cross-coupling of bromides **6** with 4-fluorophenylmagnesium bromide are summarised in Table1. Preliminary studies with iron(III) chloride/TMEDA (*N*,*N*,*N′*,*N′*-tetramethylethylenediamine; Table1, entries 1 and 2) and (FeCl_3_)_2_(TMEDA)_3_^4^ (Table1, entry 3) mainly returned unreacted starting material. However, a switch to iron(III) acetylacetonate (acac) in combination with TMEDA and hexamethylenetetramine (HMTA)^4^ showed some promise, with *cis*-**6** giving a 16 % yield of coupling product **11** as a 78:22 mixture of *trans*/*cis* isomers (Table1, entry 4). The same reaction using *trans*-**6** gave a lower yield, with a similar ratio of *trans*- and *cis*-**11** (Table1, entry 5). Hence, these results demonstrate the expected diastereoconvergence.

**Table 1:** Investigation of the cross-coupling step. 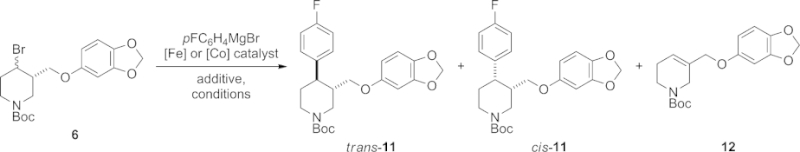


Entry	*dr* of6[a][b]	Catalyst (equiv.)	Additive (equiv.)	ArMgBr [equiv.]	Conditions	*dr* of11[a][c]	Ratio6:11:12[d]	Yield [%][e]
1	<1:99	FeCl_3_ (0.05)	TMEDA[f] (1.2)	1.2[g]	THF, 0.5 h, 0 °C	–	–	<1
2	59:41	FeCl_3_ (0.05)	TMEDA (1.2)	1.2[g]	THF, 18 h, 0 °C to r.t.	–	72:<1:27	<1
3	>99:1	[(FeCl_3_)_2_(TMEDA)_3_] (0.05)	–	1.3[g]	THF, 0.5 h, r.t.	–	–	0
4	<1:99	Fe(acac)_3_ (0.05)	TMEDA (0.1), HMTA[h] (0.05)	1.3[g]	THF, 0.5 h, 0 °C	78:22	67:32:<1	16
5	>99:1	Fe(acac)_3_ (0.05)	TMEDA (0.1), HMTA (0.05)	1.3[g]	THF, 4 h, 0 °C	79:21	73:27:<1	7
6	11:89	Fe(acac)_3_ (0.1)	TMEDA (0.1), HMTA (0.05)	2.0[g]	THF, 23 h, r.t.	77:23	46:45:9	20
7	<1:99	Fe(acac)_3_ (0.05)	TMEDA (0.1), HMTA (0.05)	1.3[g]	Et_2_O, 4 h, 0 °C	74:26	69:23:8	<10
8	<1:99	Fe(acac)_3_ (0.1)	NMP (5.8)	3.2[g]	THF, 7 h, 0 °C	66:34	85:10:5	3
9	30:70	bmim-FeCl_4_ (0.05)	–	1.5[i]	MeTHF, 0.5 h, 0 °C to r.t.	68:32	67:15:18	12
10	>99:1	Co(acac)_3_ (0.05)	TMEDA (0.05)	1.1[g]	THF, 40 min, 0 °C	83:17	69:25:6	20
11	<1:99	Co(acac)_3_ (0.05)	TMEDA (1.0)	2.1[g]	THF, 3 h, 0 °C	87:13	46:53:<1	31
12	>99:1	Co(acac)_3_ (0.05)	TMEDA (0.05)	2.0[g]	Et_2_O, 1 h, 0 °C	79:21	71:29:<1	n.d.
13	>99:1	Co(acac)_3_ (0.05)	TMEDA (0.5), HMTA (0.1)	2.0[i]	MeTHF, 2 h, 0 °C to r.t.	89:11	45:50:5	43
14	>99:1	Co(acac)_3_ (0.1)	TMEDA (0.5), HMTA (0.5)	2.0[i]	MeTHF, 2 h, 0 °C to r.t.	90:10	15:80:5	77
15	>99:1	Co(acac)_3_ (0.1)	TMEDA (0.5), HMTA (0.5)	2.0[i]	MeTHF, 2 h, –5 °C	88:12	43:51:6	26
16	30:70	Co(acac)_3_ (0.1)	TMEDA (0.5), HMTA (0.5)	2.0[j]	MeTHF, 2 h, 0 °C to r.t.	88:12	58:29:14	26
17	>99:1	Co(acac)_3_ (0.1)	bpy[k] (0.5), HMTA (0.5)	2.0[i]	MeTHF, 2 h, 0 °C to r.t.	71:29	89:8:3	4
18[l]	30:70	Co(acac)_3_ (0.1)	TMEDA (0.5), HMTA (0.5)	2.0[i]	MeTHF, 5 h, 0 °C to r.t.	88:12	11:88:<1	76

[a] *trans*/*cis*.

[b] Determined by ^1^H NMR spectroscopy.

[c] Determined by ^19^F NMR spectroscopy of the crude material.

[d] Determined by ^1^H NMR spectroscopy of the crude material.

[e] Isolated yield of **11**.

[f] *N*,*N*,*N′*,*N′*-Tetramethylethylenediamine.

[g] Added as a 1 m solution in THF.

[h] Hexamethylenetetramine.

[i] Added as a 1 m solution in MeTHF.

[j] Added as a 0.5 m solution in MeTHF.

[k] 2,2′-Bipyridine.

[l] Reaction carried out on 1.05 g (2.53 mmol) of **6**.

Increasing the temperature and the amount of Grignard reagent used led to a modest increase in yield (Table1, entry 6), but the product mixture now contained significant levels of elimination product **12**. It is unclear whether **12** was formed by a reductive elimination process, or by degradation of the starting material. The formation of thermodynamically more stable alkene **12** as the only observed elimination product suggests that the latter process predominates. In the iron-catalysed coupling reaction between tolylmagnesium bromide and 5-phenyl-1-bromopentane, Nagano and Hayashi described that the reaction was improved by using diethyl ether as the solvent instead of THF.^17^ Unfortunately, in our case, switching the solvent to diethyl ether (Table1, entry 7), lowered both the yield and the selectivity, as did the use of *N*-methyl-2-pyrrolidone (NMP) in combination with THF (Table1, entry 8).^18^ The procedure of Bica and Gaertner, i.e., the use of the ionic liquid bmim-FeCl_4_ as an iron source, was also investigated,11b but this too gave low yields and poor selectivities (Table1, entry 9).

At this juncture we decided to examine the use of more reactive Co^III^ catalysts. Pleasingly, our first reaction with Co(acac)_3_/TMEDA,^19^ gave **11** in 20 % yield (Table1, entry 10) with an improved selectivity for *trans*-**11**. The yield was elevated to 31 % by using a molar equivalent of TMEDA and 2.1 equiv. of ArMgBr (Table1, entry 11). As with the iron-catalysed examples, the selectivity dropped when diethyl ether was used as solvent (Table1, entry 12), although it improved slightly when cat. HMTA was used in MeTHF (Table1, entry 13).

A step change in performance was noted when we employed 10 mol-% of Co(acac)_3_ with a combination of TMEDA and HMTA as additives in MeTHF (Table1, entry 14).^4^ Under these conditions, the desired product (i.e., **11**) was obtained in 77 % yield with a *trans*/*cis* ratio of 90:10 (Table1, entry 14). The yield dropped significantly when the reaction was carried out at lower temperature (Table1, entry 15), though the same product ratio was obtained. Attempts to increase the selectivity by adding a more dilute solution of the Grignard reagent (Table1, entry 16) or by substituting TMEDA with bpy (Table1, entry 17) were also unsuccessful. Pleasingly, using our optimised conditions on a gram scale (Table1, entry 18) with a diastereoisomeric mixture of bromide **6** gave paroxetine precursor **11** as a separable 88:12 mixture of *trans* and *cis* diastereomers in 76 % yield. Comparison between Table1, entries 14 and 18 again confirmed the diastereoconvergence of the process.

**Scheme 5:**
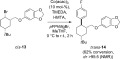
Control experiment with a conformationally locked cyclohexane derivative.

Although the diastereoselectivity was satisfactory, it did not match the levels obtained with locked cyclohexyl derivative *cis*-**1** (i.e., 96:4; Scheme 1). As a control experiment, we decided to prepare cyclohexyl analogue *cis*-**13** and test it, using our optimised conditions, in a cross-coupling reaction with (4-fluorophenyl)magnesium bromide (Scheme 5). Surprisingly, it gave *trans*-**14** as the sole reaction product, as judged by ^1^H and ^19^F NMR spectroscopic analysis. The strong conformational lock imposed by the *tert*-butyl group provides a possible explanation for the improved diastereoselectivity obtained in this system compared to the *N*-Boc-piperidine substrates (see Table1). However, it is also plausible that *N*-Boc chelation prior to the reductive elimination provides additional stabilisation for an axial organocobalt intermediate.

**Scheme 6:**
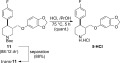
Completion of the synthesis.

Finally, the synthesis of (±)-paroxetine was completed by removal of the Boc protecting group (Scheme 6). Following Jacobsen's conditions,20a our target **5·**HCl was obtained in quantitative yield as off-white crystals by recrystallisation from 2-propanol.20b All spectroscopic data matched literature values.20b

## Conclusions

In conclusion, we have developed a short route to (±)-paroxetine using a cobalt-mediated cross-coupling reaction to construct the 3,4-disubstituted piperidine scaffold. The key step is notable for being diastereoconvergent, consistent with reported mechanistic studies. Importantly, for bromocyclohexane **13**, the diastereoselectivity was essentially complete, whereas for *N*-Boc-piperidine **6** it dropped to 9:1. Notably, our synthesis of (±)-paroxetine **5** is unique in that the *p*-fluorophenyl ring is introduced in the penultimate step. An enantioselective total synthesis is currently under investigation.

## Experimental Section

***N*-Boc-4-hydroxy-3-(tosyloxymethyl)piperidine (9):** A solution of *p*-toluenesulfonyl chloride (1.26 g, 6.61 mmol, 1.1 equiv.) in anhydrous CH_2_Cl_2_ (11 mL) was added to a solution of *N*-Boc-3-hydroxymethyl-4-piperidol **7** (1.39 g, 6.01 mmol, 1 equiv.), dry triethylamine (1.76 mL, 12.62 mmol, 2.1 equiv.), and DMAP (73 mg, 0.60 mmol, 0.1 equiv.) in dry CH_2_Cl_2_ (22 mL) under argon at room temp. The reaction mixture was stirred at room temp. After 23 h, water (36 mL) was added, and the product was extracted with CH_2_Cl_2_ (3 × 36 mL). The combined organic layers were washed with brine (36 mL), dried with magnesium sulfate, and concentrated under reduced pressure. The crude mixture was purified by column chromatography (petroleum ether/acetone, from 80:20 to 70:30) to give **9** [1.79 g, 77 %, 67:33 *dr* (*trans*:*cis*)] as a colourless oil. The two diastereoisomers could be separated by HPLC (hexane/acetone 80:20).

**Figure:**
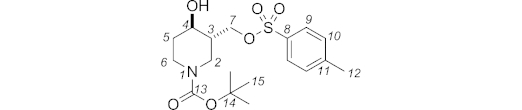


Data for *trans* diastereoisomer: *R*_f_ (hexane/acetone, 80:20): 0.18. IR (neat): 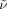 = 3424 (br. w), 2975 (w), 2929 (w), 1694 (m), 1669 (m), 1428 (br. m), 1175 (s), 959 (br. w) cm^–1^. ^1^H NMR (400 MHz, [D_6_]DMSO, 343 K): *δ* = 7.78 (d, *J* = 8.2 Hz, 2 H, 9-H or 10-H), 7.49 (d, *J* = 8.1 Hz, 2 H, 9-H or 10-H), 4.24 (dd, *J* = 10.0, 3.8 Hz, 1 H, 7-H), 3.98 (dd, *J* = 10.0, 8.1 Hz, 1 H, 7-H), 3.86 (br. ddd, *J* = 13.4, 4.0, 1.8 Hz, 1 H, 2_eq_-H), 3.78 (dtd, *J* = 13.4, 4.3, 1.9 Hz, 1 H, 6_eq_-H), 3.34 (td, *J* = 9.5, 4.4 Hz, 1 H, 4_ax_-H), 2.77 (ddd, *J* = 13.5, 11.6, 3.0 Hz, 1 H, 6_ax_-H), 2.57 (dd, *J* = 13.1, 10.5 Hz, 1 H, 2_ax_-H), 2.43 (s, 3 H, 12-H), 1.74 (dq, *J* = 12.9, 3.7 Hz, 1 H, 5_eq_-H), 1.52–1.64 (m, 1 H, 3-H), 1.39 (s, 9 H, 15-H), 1.24 (dddd, *J* = 12.9, 11.6, 10.0, 4.5 Hz, 1 H, 5_ax_-H) ppm. ^13^C NMR + DEPT 135 (100 MHz, [D_6_]DMSO, 343 K): *δ* = 153.6, 144.5 (C, C-8, C-13), 132.4 (C, C-11), 129.8, 127.1 (CH, C-9, C-10), 78.5 (C, C-14), 69.7 (CH_2_, C-7), 66.4 (CH, C-4), 43.6 (CH_2_, C-2), 42.9 (CH, C-3), 41.5 (CH_2_, C-6), 33.2 (CH_2_, C-5), 27.7 (CH_3_, C-15), 20.7 (CH_3_, C-12) ppm. MS (ES^+^): *m*/*z* = 408.2 [M + Na]^+^. HRMS (ES^+^): calcd. for C_18_H_27_NNaO_6_S^+^ [M + Na]^+^ 408.1451; found 408.1454.

**Figure:**
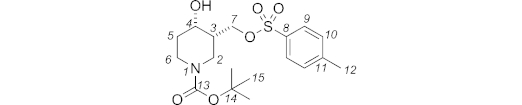


Data for *cis* diastereoisomer: *R*_f_ (hexane/acetone, 80:20): 0.21. IR (neat): 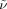 = 3425 (br. w), 2975 (w), 2927 (w), 1694 (m), 1670 (m), 1428 (br. m), 1175 (s), 959 (br. w) cm^–1^. ^1^H NMR (400 MHz, [D_6_]DMSO, 363 K): *δ* = 7.77 (d, *J* = 8.3 Hz, 2 H, 9-H or 10-H), 7.48 (d, *J* = 8.1 Hz, 2 H, 9-H or 10-H), 4.13 (dd, *J* = 9.9, 5.2 Hz, 1 H, 7-H), 3.92 (dd, *J* = 9.9, 9.4 Hz, 1 H, 7-H), 3.84 (q, *J* = 4.0 Hz, 1 H, 4_eq_-H), 3.49 (dd, *J* = 13.1, 3.8 Hz, 1 H, 2_eq_-H), 3.43 (dt, *J* = 13.3, 4.8 Hz, 1 H, 6_eq_-H), 3.23 (ddd, *J* = 13.3, 7.6, 5.8 Hz, 1 H, 6_ax_-H), 3.04 (app. dd, *J* = 12.9, 9.3 Hz, 1 H, 2_ax_-H), 2.44 (s, 3 H, 12-H), 1.87 (tdt, *J* = 9.4, 5.2, 3.8 Hz, 1 H, 3_ax_-H), 1.46–1.53 (m, 2 H, 2 5-H), 1.39 (s, 9 H, 15-H) ppm. ^13^C NMR + DEPT 135 (100 MHz, [D_6_]DMSO, 343 K): *δ* = 153.7, 144.4 (C, C-8, C-13), 132.5 (C, C-11), 129.8, 127.1 (CH, C-9, C-10), 78.3 (C, C-14), 69.5 (CH_2_, C-7), 63.5 (CH, C-4), 40.7 (CH_2_, C-2), 39.7 (CH, C-3), 38.7 (CH_2_, C-6), 31.4 (CH_2_, C-5), 27.7 (CH_3_, C-15), 20.7 (CH_3_, C-12) ppm. MS (ES^+^): *m*/*z* = 408.2 [M + Na]^+^. HRMS (ES^+^): calcd. for C_18_H_27_NNaO_6_S^+^ [M + Na]^+^ 408.1451; found 408.1458.

**Figure:**
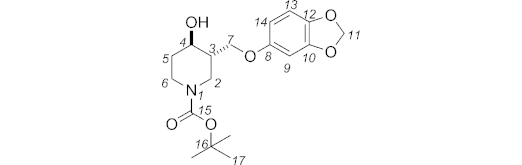


Data for *trans* diastereoisomer: *R*_f_ (hexane/acetone, 80:20): 0.18. IR (neat): 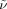 = 3424 (br. w), 2975 (w), 2929 (w), 1694 (m), 1669 (m), 1428 (br. m), 1175 (s), 959 (br. w) cm^–1^. ^1^H NMR (400 MHz, [D_6_]DMSO, 343 K): *δ* = 7.78 (d, *J* = 8.2 Hz, 2 H, 9-H or 10-H), 7.49 (d, *J* = 8.1 Hz, 2 H, 9-H or 10-H), 4.24 (dd, *J* = 10.0, 3.8 Hz, 1 H, 7-H), 3.98 (dd, *J* = 10.0, 8.1 Hz, 1 H, 7-H), 3.86 (br. ddd, *J* = 13.4, 4.0, 1.8 Hz, 1 H, 2_eq_-H), 3.78 (dtd, *J* = 13.4, 4.3, 1.9 Hz, 1 H, 6_eq_-H), 3.34 (td, *J* = 9.5, 4.4 Hz, 1 H, 4_ax_-H), 2.77 (ddd, *J* = 13.5, 11.6, 3.0 Hz, 1 H, 6_ax_-H), 2.57 (dd, *J* = 13.1, 10.5 Hz, 1 H, 2_ax_-H), 2.43 (s, 3 H, 12-H), 1.74 (dq, *J* = 12.9, 3.7 Hz, 1 H, 5_eq_-H), 1.52–1.64 (m, 1 H, 3-H), 1.39 (s, 9 H, 15-H), 1.24 (dddd, *J* = 12.9, 11.6, 10.0, 4.5 Hz, 1 H, 5_ax_-H) ppm. ^13^C NMR + DEPT 135 (100 MHz, [D_6_]DMSO, 343 K): *δ* = 153.6, 144.5 (C, C-8, C-13), 132.4 (C, C-11), 129.8, 127.1 (CH, C-9, C-10), 78.5 (C, C-14), 69.7 (CH_2_, C-7), 66.4 (CH, C-4), 43.6 (CH_2_, C-2), 42.9 (CH, C-3), 41.5 (CH_2_, C-6), 33.2 (CH_2_, C-5), 27.7 (CH_3_, C-15), 20.7 (CH_3_, C-12) ppm. MS (ES^+^): *m*/*z* = 408.2 [M + Na]^+^. HRMS (ES^+^): calcd. for C_18_H_27_NNaO_6_S^+^ [M + Na]^+^ 408.1451; found 408.1454.

***N*-Boc-3-{[3,4-(methylenedioxy)phenoxy]methyl}piperidin-4-ol (10):** NaOH (50 % aq.; 2.9 mL) was added to a mixture of *N*-Boc-4-hydroxy-3-[(tosyloxy)methyl]piperidine **9** (*cis* and *trans*; 591 mg, 1.53 mmol, 1 equiv.), tetra-*n*-butylammonium hydroxide (1.0 m in H_2_O; 77 μL, 0.08 mmol, 0.05 equiv.), and sesamol (233 mg, 1.69 mmol, 1.1 equiv.) in toluene (5.7 mL). The reaction mixture was stirred at 70 °C. After 25 h, the aqueous and organic phases were separated. The aqueous phase was extracted with EtOAc (3 × 20 mL), then the combined organic phases were dried with magnesium sulfate and concentrated under reduced pressure. The crude mixture was purified by column chromatography (petroleum ether/EtOAc, 60:40) to give **10** (412 mg, 76 %) as a yellow oil. The two diastereoisomers were separated by HPLC (hexane/EtOAc, 60:40) to give the *trans* diastereoisomer (280 mg, 52 %) as a pale yellow oil, and the *cis* diastereoisomer (128 mg, 24 %) as a colourless oil.

**Figure:**
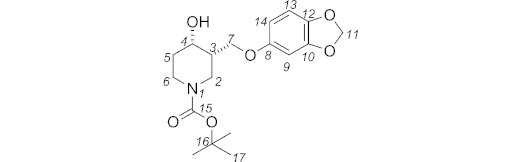


Data for *trans* diastereoisomer: *R*_f_ (hexane/EtOAc, 50:50): 0.27. IR (neat): 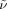 = 3049 (br. w), 2975 (w), 2927 (br. w), 1686 (br. s), 1670 (br. s), 1489 (s), 1182 (br. s), 1038 (m) cm^–1^. ^1^H NMR (400 MHz, [D_6_]DMSO, 363 K): *δ* = 6.77 (d, *J* = 8.5 Hz, 1 H, 13-H), 6.58 (d, *J* = 2.5 Hz, 1 H, 9-H), 6.38 (dd, *J* = 8.5, 2.5 Hz, 1 H, 14-H), 5.93 (s, 2 H, 11-H), 4.56 (d, *J* = 5.4 Hz, 1 H, OH), 4.12 (dd, *J* = 9.9, 3.7 Hz, 1 H, 7-H), 4.00 (ddd, *J* = 13.3, 4.3, 1.9 Hz, 1 H, 2_eq_-H), 3.84 (dd, *J* = 10.0, 8.0 Hz, 1 H, 7-H), 3.81 (dtd, *J* = 13.3, 4.3, 1.8 Hz, 1 H, 6_eq_-H), 3.50 (tdd, *J* = 9.2, 5.3, 4.8 Hz, 1 H, 4_ax_-H), 2.88 (ddd, *J* = 13.4, 11.1, 3.2 Hz, 1 H, 6_ax_-H), 2.78 (dd, *J* = 13.3, 9.9 Hz, 1 H, 2_ax_-H), 1.81 (dtd, *J* = 12.9, 4.3, 3.5 Hz, 1 H, 5_eq_-H), 1.71 (ddddd, *J* = 9.9, 9.2, 8.0, 4.3, 3.7 Hz, 1 H, 3_ax_-H), 1.40 (s, 9 H, 17-H), 1.33 (dddd, *J* = 12.9, 11.1, 9.2, 4.3 Hz, 2 H, 5_ax_-H) ppm. ^13^C NMR + DEPT 135 (100 MHz, [D_6_]DMSO, 343 K): *δ* = 154.0, 153.7, 147.6, 140.9 (C, C-15, C-8, C-10, C-12), 107.6 (CH, C-13), 105.9 (CH, C-14), 100.6 (CH_2_, C-11), 97.7 (CH, C-9), 78.3 (C, C-16), 67.9 (CH_2_, C-7), 66.9 (CH, C-4), 44.2 (CH_2_, C-2), 43.2 (CH, C-3), 41.4 (CH_2_, C-6), 33.3 (CH_2_, C-5), 27.7 (CH_3_, C-17) ppm. MS (ES^+^): *m*/*z* = 374.2 [M + Na]^+^. HRMS (ES^+^): calcd. for C_18_H_25_NNaO_6_^+^ [M + Na]^+^ 374.1574; found 374.1577.

Data for *cis* diastereoisomer: *R*_f_ (hexane/EtOAc, 60:40): 0.24. IR (neat): 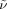 = 3446 (br. w), 2974 (w), 2928 (w), 1665 (br. m), 1489 (s), 1183 (br. s), 1038 (m) cm^–1^. ^1^H NMR (400 MHz, [D_6_]DMSO, 363 K): *δ* = 6.77 (d, *J* = 8.6 Hz, 1 H, 13-H), 6.57 (d, *J* = 2.5 Hz, 1 H, 9-H), 6.37 (dd, *J* = 8.6, 2.5 Hz, 1 H, 14-H), 5.93 (s, 2 H, 11-H), 4.56 (br. d, *J* = 3.5 Hz, 1 H, OH), 3.99 (dd, *J* = 9.9, 5.3 Hz, 1 H, 7-H), 3.94 (app. t, *J* = 3.5 Hz, 1 H, 4_eq_-H), 3.76 (dd, *J* = 9.6, 8.6 Hz, 1 H, 7-H), 3.58 (br. d, *J* = 12.6, 3.5 Hz, 1 H, 2_eq_-H), 3.46 (dt, *J* = 12.9, 4.9 Hz, 1 H, 6_eq_-H), 3.32 (m, 1 H, 6-H), 3.20 (m, 1 H, 2-H), 1.96 (m, 1 H, 3-H), 1.51–1.62 (m, 2 H, 5-H), 1.37 (s, 9 H, 17-H) ppm. ^13^C NMR + DEPT 135 (100 MHz, [D_6_]DMSO, 353 K): *δ* = 154.0, 153.8, 147.6, 140.8 (C, C-15, C-8, C-10, C-12), 107.5 (CH, C-13), 105.9 (CH, C-14), 100.5 (CH_2_, C-11), 97.6 (CH, C-9), 78.1 (C, C-16), 67.4 (CH_2_, C-7), 64.0 (CH, C-4), 41.3 (CH_2_, C-2), 40.2 (CH, C-3), 38.9 (CH_2_, C-6), 31.6 (CH_2_, C-5), 27.7 (CH_3_, C-17) ppm. MS (ES^+^): *m*/*z* = 374.2 [M + Na]^+^. HRMS (ES^+^): calcd. for C_18_H_25_NNaO_6_^+^ [M + Na]^+^ 374.1574; found 374.1580.

***N*-Boc-4-bromo-3-{[3,4-(methylenedioxy)phenoxy]methyl}piperidine (6):** A mixture of *N*-Boc-3-{[3,4-(methylenedioxy)phenoxy]methyl}piperidin-4-ol **10** (1.29 g, 3.67 mmol, 1.0 equiv.) and imidazole (300 mg, 4.41 mmol, 1.2 equiv.) in dry dichloromethane (8.5 mL) was cooled to 0 °C. A solution of bromotriphenylphosphonium bromide (1.704 g, 4.04 mmol, 1.1 equiv.) in dry CH_2_Cl_2_ (9.1 mL) was stirred at room temp. for 15 min, and then added dropwise to the starting material. The reaction mixture was stirred in the dark for 24 h at room temp., then it was diluted with water (25 mL) and extracted with CH_2_Cl_2_ (3 × 70 mL). The organic layer was dried with sodium sulfate and concentrated under reduced pressure. The crude mixture was purified by column chromatography (petroleum ether/EtOAc, 90:10) to give **6** [1.521 g, 65 %, 70:30 *dr* (*cis*/*trans*)] as a colourless oil. The two diastereoisomers could be separated by selective precipitation of *cis*-**6** from hexane/diethyl ether (90:10).

**Figure:**
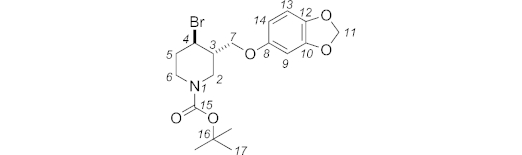


Data for *trans* diastereoisomer: *R*_f_ (petroleum ether 40–60 °C/EtOAc, 90:10): 0.20. IR (neat): 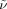 = 2957 (w), 1698 (m), 1489 (m), 1264 (s), 1184 (br. w), 730 (br. s), 703 (m), 651 (w) cm^–1^. ^1^H NMR (400 MHz, [D_6_]DMSO, 373 K): *δ* = 6.78 (d, *J* = 8.3 Hz, 1 H, 13-H), 6.60 (d, *J* = 2.4 Hz, 1 H, 9-H), 6.41 (dd, *J* = 8.3, 2.4 Hz, 1 H, 14-H), 5.94 (s, 2 H, 11-H), 4.41 (td, *J* = 9.7, 4.3 Hz, 1 H, 4_ax_-H), 4.11 (dd, *J* = 10.1, 3.6 Hz, 1 H, 7-H), 4.05 (ddd, *J* = 13.6, 4.2, 2.0 Hz, 1 H, 2_eq_-H), 3.98 (dd, *J* = 10.2, 7.0 Hz, 1 H, 7-H), 3.78 (dtd, *J* = 13.5, 4.4, 1.9 Hz, 1 H, 6_eq_-H), 2.98–3.09 (m, 2 H, 2-H, 6-H), 2.28 (dtd, *J* = 13.2, 4.3, 3.2 Hz, 1 H, 5_eq_-H), 2.06–2.17 (m, 1 H, 3-H), 1.84–1.97 (m, 1 H, 5-H), 1.42 (s, 9 H, 17-H) ppm. ^13^C NMR + DEPT 135 (100 MHz, [D_6_]DMSO, 353 K): *δ* = 153.5, 153.5, 147.6, 141.2 (C, C-15, C-8, C-10, C-12), 107.6 (CH, C-13), 106.1 (CH, C-14), 100.6 (CH_2_, C-11), 97.8 (CH, C-9), 78.7 (C, C-16), 69.1 (CH_2_, C-7), 51.6 (CH, C-4), 45.2 (CH_2_, C-2), 44.1 (CH, C-3), 43.0 (CH_2_, C-6), 35.5 (CH_2_, C-5), 27.6 (CH_3_, C-17) ppm. MS (ES^+^): *m*/*z* = 436.1, 438.0 [M + Na]^+^. HRMS (ES^+^): calcd. for C_18_H_24_^79^BrNNaO_5_^+^ [M + Na]^+^ 436.0730; found 436.0733.

**Figure:**
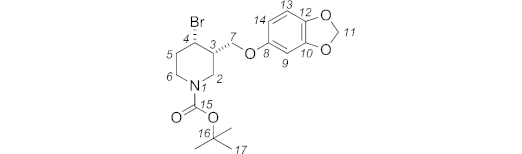


Data for *cis* diastereoisomer: *R*_f_ (petroleum ether 40–60 °C/EtOAc, 90:10): 0.17. IR (neat): 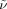 = 2958 (w), 1696 (m), 1489 (m), 1265 (s), 1183 (br. m), 732 (br. s), 702 (m), 650 (w) cm^–1^. ^1^H NMR (400 MHz, [D_6_]DMSO, 353 K): *δ* = 6.78 (d, *J* = 8.6 Hz, 1 H, 13-H), 6.61 (d, *J* = 2.5 Hz, 1 H, 9-H), 6.40 (dd, *J* = 8.6, 2.5 Hz, 1 H, 14-H), 5.94 (s, 2 H, 11-H), 4.86 (app. q, *J* = 3.2 Hz, 1 H, 4_eq_-H), 3.81–3.96 (m, 3 H, 2-H, 2 7-H), 3.77 (dt, *J* = 13.4, 4.2 Hz, 1 H, 6_eq_-H), 3.21 (ddd, *J* = 13.8, 10.0, 4.0 Hz, 1 H, 6_ax_-H), 2.97 (br. dd, *J* = 13.1, 10.1 Hz, 1 H, 2_ax_-H), 1.95–2.17 (m, 3 H, 3-H, 2 5-H), 1.40 (s, 9 H, 17-H) ppm. MS (ES^+^): *m*/*z* = 436.1, 438.2 [M + Na]^+^. HRMS (ES^+^): calcd. for C_18_H_24_^79^BrNNaO_5_^+^ [M + Na]^+^ 436.0730; found 436.0729.

***N*-Boc-4-(*p*-fluorophenyl)-3-[3,4-(methylenedioxy)phenoxymethyl]piperidine (11):** *N*-Boc-4-bromo-3-{[3,4-(methylenedioxy)phenoxy]methyl}piperidine (**6**) (1.049 g, 2.53 mmol, 1 equiv.), Co(acac)_3_ (90 mg, 0.25 mmol, 0.1 equiv.), TMEDA (190 μL, 1.27 mmol, 0.5 equiv.), HMTA (177 mg, 1.27 mmol, 0.5 equiv.), and MeTHF (5.1 mL) were put into a flame-dried two-necked round-bottomed flask. The reaction mixture was cooled to 0 °C, then a solution of 4-fluorophenylmagnesium bromide (1.0 m in MeTHF; 5.1 mL, 5.1 mmol, 2.0 equiv.) was added over 3 h. After the addition was complete, the reaction mixture was stirred for 1 h at 0 °C, and then for 1 h at room temp. Then the mixture was quenched with aqueous HCl (1 m aq.; 7.3 mL). The aqueous layer was extracted with diethyl ether (3 × 28 mL), and the combined organic layers were dried with magnesium sulfate and concentrated under reduced pressure. The crude mixture [88:12 *dr* (*trans*:*cis*)] was purified by column chromatography (pentane/acetone, 94:6) to give **11** (826 mg, 76 %) as a colourless oil, in a mixture with unreacted starting material **6**. The mixture was purified by HPLC (pentane/acetone, 94:6) to give *trans*-**11** (722 mg, 66 %) as a colourless oil, which solidified on standing and was recrystallised from pentane, and *cis*-**11** (46 mg, 4 %) as a colourless oil.

**Figure:**
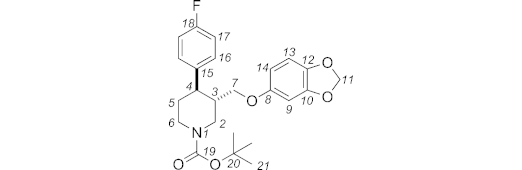


Data for *trans* diastereoisomer: *R*_f_ (pentane/acetone, 94:6): 0.13, m.p. 96.0–97.0 °C (pentane). ^1^H NMR (400 MHz, [D_6_]DMSO, 353 K): *δ* = 7.27 (dd, *J* = 8.8, 5.6 Hz, 2 H, 16-H), 7.08 (t, *J* = 8.8 Hz, 2 H, 17-H), 6.70 (d, *J* = 8.4 Hz, 1 H, 13-H), 6.40 (d, *J* = 2.5 Hz, 1 H, 9-H), 6.18 (dd, *J* = 8.6, 2.5 Hz, 1 H, 14-H), 5.90 (s, 2 H, 11-H), 4.30 (ddd, *J* = 13.1, 4.0, 1.5 Hz, 1 H, 2_eq_-H), 4.06 (ddt, *J* = 13.1, 4.0, 2.0 Hz, 1 H, 6_eq_-H), 3.58 (dd, *J* = 10.1, 3.5 Hz, 1 H, 7-H), 3.53 (dd, *J* = 10.1, 7.6 Hz, 1 H, 7-H), 2.83 (td, *J* = 12.9, 3.0 Hz, 1 H, 4_ax_-H), 2.75 (dd, *J* = 13.1, 11.1 Hz, 1 H, 2_ax_-H), 2.68 (m, 1 H, 6_ax_-H), 2.02 (tdt, *J* = 11.1, 7.6, 3.9 Hz, 1 H, 3_ax_-H), 1.73 (dtd, *J* = 13.1, 3.5, 2.5 Hz, 1 H, 5_eq_-H), 1.62 (qd, *J* = 12.6, 4.5 Hz, 1 H, 5_ax_-H), 1.44 (s, 9 H, 21-H) ppm. ^13^C NMR + DEPT 135 (100 MHz, [D_6_]DMSO, 353 K): *δ* = 160.5 (C, d, *J* = 241.0 Hz, C-18), 154.3, 153.6, 147.5, 141.0 (4 C, C-19, C-8, C-10, C-12), 139.3 (C, d, *J* = 2.9 Hz, C-15), 128.7 (CH, d, *J* = 7.8 Hz, C-16), 114.7 (CH, d, *J* = 21.4 Hz, C-17), 107.5 (CH, C-13), 105.8 (CH, C-14), 100.5 (CH_2_, C-11), 97.6 (CH, C-9), 78.3 (C, C-20), 68.9 (CH_2_, C-7), 46.4, 43.6 (CH_2_, C-2, C-6), 43.1 (CH, C-4), 40.8 (CH, C-3), 33.2 (CH_2_, C-5), 27.8 (CH_3_, C-21) ppm. ^19^F NMR (282 MHz, [D_6_]DMSO, 298 K): *δ* = –116.2 (s, 1 F) ppm.

**(±)-Paroxetine Hydrochloride Salt (5·HCl):** *trans*-*N*-Boc-4-(*p*-fluorophenyl)-3-[3,4-(methylenedioxy)phenoxymethyl]piperidine (*trans*-**11**; 286 mg, 0.67 mmol, 1 equiv.) was dissolved in 2-propanol (3.3 mL). Concentrated hydrochloric acid (101 μL, 1.00 mmol, 1.5 equiv.) was added, and the solution was stirred for 5 h at 75 °C. The resulting mixture was cooled to room temp. and concentrated under reduced pressure. The residue was dried by azeotroping with absolute ethanol (3 × 3 mL) to give **5·**HCl (243 mg, > 99 %) as an off-white solid, which was recrystallised from 2-propanol.

**Figure:**
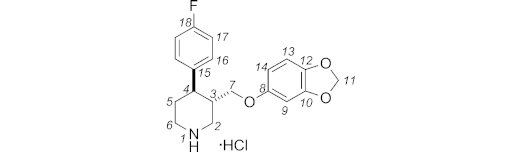


M.p. 124–126 °C (*i*PrOH). ^1^H NMR (400 MHz, CDCl_3_): *δ* = 9.94 (br. s, 2 H, NH_2_), 7.22 (dd, *J* = 8.3, 5.3 Hz, 2 H, 16-H), 6.99 (t, *J* = 8.6 Hz, 2 H, 17-H), 6.62 (d, *J* = 8.6 Hz, 1 H, 13-H), 6.33 (d, *J* = 2.5 Hz, 1 H, 9-H), 6.12 (dd, *J* = 8.3, 2.3 Hz, 1 H, 14-H), 5.88 (s, 2 H, 11-H), 3.63–3.83 (m, 2 H, 2-H, 6-H), 3.60 (br. dd, *J* = 9.6, 2.0 Hz, 1 H, 7-H), 3.48 (dd, *J* = 9.3, 4.8 Hz, 1 H, 7-H), 3.17 (br. t, *J* = 12.4 Hz, 1 H, 2-H), 3.04 (br. t, *J* = 12.1 Hz, 1 H, 6-H), 2.91 (td, *J* = 11.9, 3.0 Hz, 1 H, 4-H), 2.68 (m, 1 H, 3-H), 2.42 (m, 1 H, 5-H), 2.03 (br. d, *J* = 13.6 Hz, 1 H, 5-H) ppm. ^13^C NMR + DEPT 135 (100 MHz, CDCl_3_): *δ* = 161.9 (d, *J* = 245.9 Hz, C, C-18), 153.7, 148.2, 142.1 (C, C-8, C-10, C-12), 137.0 (d, *J* = 2.9 Hz, C, C-15), 128.9 (d, *J* = 8.8 Hz, CH, C-16), 115.8 (d, *J* = 22.0 Hz, CH, C-17), 107.9, 105.6 (CH, C-13, C-14), 101.2 (CH_2_, C-11), 97.9 (CH, C-9), 67.4 (CH_2_, C-7), 46.8 (CH_2_, C-2), 44.5 (CH_2_, C-6), 41.7 (CH, C-3), 39.4 (CH, C-4), 30.0 (CH_2_, C-5) ppm. ^19^F NMR (282 MHz, CDCl_3_): *δ* = –115.2 (s, 1 F, F18) ppm. The spectra are consistent with reported data.20b
