# Supplementary material for: Total Synthesis of (±)-Paroxetine by Diastereoconvergent Cobalt-Catalysed Arylation
Source: European J Org Chem. 2014 May 27;2014(20):4335–41. doi: 10.1002/ejoc.201402108 (PMC4255755; doi:10.1002/ejoc.201402108)
Supplement: Supplementary file 1 [file ejoc2014-4335-SD1.pdf]

Eur. J. Org. Chem. **2014** · © WILEY-VCH Verlag GmbH & Co. KGaA, 69451 Weinheim, 2014 · ISSN 1099–0690

SUPPORTING INFORMATION

DOI: 10.1002/ejoc.201402108
Title: Total Synthesis of (±)-Paroxetine by Diastereoconvergent Cobalt-Catalysed Arylation
Author(s): Carole F. Despiau, Andrew P. Dominey, David C. Harrowven, Bruno Linclau*

General. Chemical reagents were obtained from commercial sources and used without further purification, unless stated otherwise. Anhydrous solvents were distilled immediately before use. Et_2O and THF were distilled from Na/benzophenone immediately. Toluene was distilled from Na. DCM, MeOH, *i*PrOH, TMEDA and Et_3N were dried over CaH_2 and distilled prior to use. All glassware was flame-dried under vacuum and cooled under Ar prior to use. Water or air sensitive reactions were performed under inert atmosphere, using dry solvents.

Reactions were monitored by TLC (MERCK Kieselgel 60 F_{254}, aluminium sheet). Detection was carried out using the following dying reagent:

$KMnO_4$-reagent: a solution of 3 g $KMnO_4$, 20 g K_2CO_3 and 5 mL NaOH (aq., 5 w%) in 300 mL H_2O gives a reagent that will show yellow spots after development with a heat gun.

Column chromatography was performed on silica gel (60 Å, particle size 40–63 µm). All reported solvent mixtures are volume measures.

Preparative HPLC was carried out using Biorad Bio-Sil D 90-10 columns (250 × 22 mm at 15–20 mL min^{-1} and 250 × 10 mm at 5 mL min^{-1}).

^1H, ^{19}F and ^{13}C NMR spectra were recorded at room temperature on a BRUKER AV300 or a DPX400 spectrometer. High temperature ^1H and ^{13}C NMR spectra were recorded on a BRUKER DPX400 spectrometer. ^1H and ^{13}C chemical shifts (δ) are quoted in ppm relative to residual solvent peaks as appropriate. ^{19}F spectra were externally referenced to $CFCl_3$. The coupling constants (*J*) are given in Hertz (Hz). The proton NMR signals signals were designated as follows: s (singlet), d (doublet), t (triplet), q (quartet), quin (quintet), sxt (sextet), spt (septet), m (multiplet), or a combination of the above. The coupling constants are uncorrected.

IR spectra were recorded as neat films on a Nicolet 380 FT-IR. Absorption peaks are given in cm^{-1} and the intensities were designated as follows: w (weak), m (medium), s (strong), br. (broad).

Optical rotations were recorded on an OPTICAL ACTIVITY POLAAR 2001 polarimeter at 589 nm.

Melting points were recorded on a Reichert melting point apparatus, equipped with a Reichert microscope.

Low resolution ES mass spectra were recorded on a WATERS ZMD single quadrupole system. High resolution ES mass spectra were recorded on a BRUKER MaXis ESI-TOF.

Low resolution EI and CI mass spectra were recorded on a THERMO Trace Gas chromatography-mass spectrometer (GC-MS) equipped with a single quadrupole analyzer. High resolution EI and CI mass spectra were recorded on a VG 70-250SE mass spectrometer.

Experimental for the synthesis of (±)-paroxetine

1. *N*-Boc-3-hydroxymethyl-4-piperidol (7)

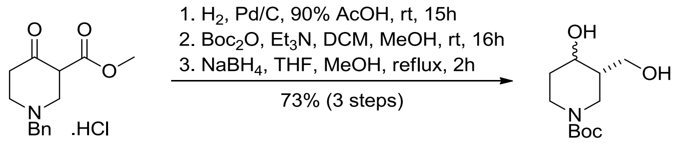

8 **7**
58:42 *dr*
(*trans*:*cis*)

Following the method of Lázár,[1] methyl 1-benzyl-4-oxo-3-piperidinecarboxylate hydrochloride **8** (20.0 g, 70.48 mmol, 1 equiv.) was dissolved in 90% (w/w) acetic acid (120 mL). The mixture was hydrogenated in the presence of 10% palladium on carbon (2.03 g, 1.90 mmol, 0.027 equiv.) for 15 h. The reaction mixture was filtered on celite, washed with methanol (3.0 L) then concentrated under reduced pressure to give a red solid. The crude solid was recrystallized from hot methanol and washed with cold diethyl ether to give methyl 4-oxo-3-piperidinecarboxylate hydrochloride as a pale red solid (13.6 g, quantitative). A solution of di-*tert*-butyl dicarbonate (2.85 mL, 12.41 mmol, 1.2 equiv.) in dry DCM (4.8 mL) was then added dropwise at 0 °C to a stirred solution of methyl 4-oxo-3-piperidinecarboxylate hydrochloride (2.000 g, 10.34 mmol, 1 equiv.) and triethylamine (2.02 mL, 14.48 mmol, 1.4 equiv.) in dry DCM (2.7 mL) and dry MeOH (2.5 mL). After being stirred at rt for 16 h, the reaction mixture was washed with HCl (1 M, 2 x 20 mL) and brine (2 x 12 mL). The organic layer was dried over sodium sulphate and evaporated to dryness under reduced pressure. The crude mixture was purified by column chromatography (petroleum ether 40-60 °C/acetone 96:4) to give *N*-Boc-3-methyl-4-oxopiperidine-1,3-dicarboxylate as a colourless oil (2.100 g, 79%).
Following the method of Soai,[2] methanol (6.8 mL) was added dropwise over a period of 1 h to a refluxing mixture of *N*-Boc-3-methyl-4-oxopiperidine-1,3-dicarboxylate (2.100 g, 8.16 mmol, 1 equiv.) and sodium borohydride (926 mg, 24.49 mmol, 3 equiv.) in THF (34 mL). Stirring was continued at reflux for 1 h. After cooling to rt, 1 M HCl (10 mL) was added, the mixture was extracted with ether (5 x 40 mL) and the organic phases were combined and dried with sodium sulphate. The solvent was evaporated under reduced pressure. The crude product was purified by column chromatography (petroleum ether/acetone 60:40) to give **7** as a colourless oil (1.763 g, 93%, 58:42 *dr* (*trans*:*cis*)).

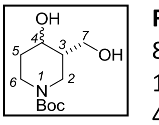

$\mathbf{R_f}$ 0.27 (hexane/acetone 50:50); **IR** (neat) 3385 (br. w), 2929 (w), 1662 (br. s), 1427 (m), 1165 (s), 1053 (br. m), 877 (w), 734 (w), 591 (br. w) cm^{-1}; **^{1}H NMR** (400MHz, $CDCl_3$) δ (ppm) 3.31–4.35 (6H, m), 2.33–3.30 (3H, m), 1.62–2.07 (3H, m), 1.45 (9H, s); **^{1}H NMR** (performed on 71:29 *dr* (*trans*:*cis*)) (400MHz, DMSO-d_6, 343 K) δ (ppm) 4.46 (1H, d, *J* 5.1 Hz, O<u>H</u>-C4 *trans*), 4.36 (0.4H, d, *J* 3.9 Hz, O<u>H</u>-C4 *cis*), 4.24 (1H, t, *J* 5.2 Hz, O<u>H</u>-C7 *trans*), 4.16 (0.4H, t, *J* 5.2 Hz, O<u>H</u>-C7 *cis*); 3.94 (1H, ddd, *J* 13.3, 4.4, 2.0 Hz, $H2_{eq}$ *trans*), 3.88 (0.4H, m, H4 *cis*), 3.80 (1H, dtd, *J* 13.3, 4.2, 2.0 Hz, $H6_{eq}$ *trans*), 3.59–3.68 (1.4H, m, H7 *trans*, H2 *cis*), 3.55 (0.4H, dtd, *J* 12.9, 4.8, 1.4 Hz, H6 *cis*), 3.48 (0.4H, m, H7 *cis*), 3.26–3.43 (2.4H, m, H4 *trans*, H7 *trans*, H7 *cis*), 3.17 (0.4H, m, H6 *cis*), 2.95 (0.4H, br. t, *J* 11.5 Hz, H2 *cis*), 2.78 (1H, ddd, *J* 13.3, 11.5, 3.0 Hz, $H6_{ax}$ *trans*), 2.58 (1H, dd, *J* 13.2, 10.4 Hz, $H2_{ax}$ *trans*), 1.75 (1H, m, H5 *trans*), 1.32–1.64 (14.8H, m, H3 *trans*, H3 *cis*, 2 H5 *cis*, CH_3 *trans* and *cis*), 1.26 (1H, dddd, *J* 12.7, 11.5, 9.8, 4.5 Hz, H5 *trans*); **^{13}C NMR + DEPT 135** (100MHz, DMSO-d_6, 343 K) δ (ppm) 153.9 (C, C=O *trans*), 153.8 (C, C=O *cis*), 78.1 (C, <u>C</u>(CH_3)$_3$ *trans*), 78.0 (C, <u>C</u>(CH_3)$_3$ *cis*), 67.6 (CH, C4 *trans*), 64.0 (CH, C4 *cis*), 60.5 (CH_2, C7 *trans*), 60.4 (CH_2, C7 *cis*), 45.7 (CH, C3 *trans*), 44.4 (CH_2, C2 *trans*), 42.9 (CH, C3 *cis*), 41.6 (CH_2, C6 *trans*), 41.3 (CH_2, C2 *cis*), 38.6 (CH_2, C6 *cis*), 33.4 (CH_2, C5 *trans*), 32.1 (CH_2, C5 *cis*), 27.8 (CH_3, $CH_{3,\,Boc}$ *trans* and *cis*); **ES$^+$MS** m/z 254.2 $(M+Na)^+$, 295.2 $(M+Na+MeCN)^+$; **HRMS** (ES$^+$) for $C_{11}H_{21}NNaO_4^+$ $(M+Na)^+$ calcd. 254.1363, found. 254.1368.

2. *N*-Boc-4-hydroxy-3-(tosyloxymethyl)piperidine (9)

***Trans-N*-Boc-4-hydroxy-3-(tosyloxymethyl)piperidine**

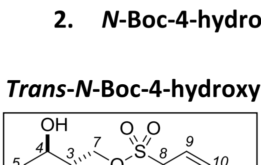

^{1}H NMR (400MHz, $CDCl_3$) δ (ppm) 7.80 (2H, d, *J* 8.1 Hz, H9 or H10), 7.36 (2H, d, *J* 8.1 Hz, H9 or H10), 3.39–4.57 (5H, m, H2, H4, H6, 2 H7), 2.58–2.89 (2H, m, H2, H6), 2.46 (3H, s, H12), 1.61–2.04 (3H, m, H3, 2 H5), 1.44 (9H, s, H15); **^{13}C NMR + DEPT 135** (100MHz, $CDCl_3$) δ (ppm) 154.6, 145.0 (C, C8, C13), 132.6 (C, C11), 130.0, 127.9 (CH, C9, C10), 80.0 (C, C14), 68.9 (CH_2, C7), 68.1 (CH, C4), 43.4 (CH, C3), C2 and C6 broad signals around 43 ppm, 33.7 (CH_2, C5), 28.3 (CH_3, C15), 21.6 (CH_3, C12); **^{1}H NMR** (400MHz, DMSO-d_6, 298 K) δ (ppm) 7.78 (2H, d, *J* 8.1 Hz, H9 or H10), 7.49 (2H, d, *J* 7.8 Hz, H9 or H10), 4.89 (1H, d, *J* 5.4 Hz, OH), 4.19 (1H, dd, *J* 9.9, 3.2 Hz, H7), 3.94 (1H, br. t, *J* 9.2 Hz, H7), 3.68–3.91 (2H, m, H2, H6), 3.31 (1H, m, H4), 2.52–2.83 (2H, m, H2, H6), 2.42 (3H, s, H12), 1.72 (1H, m, H5), 1.53 (1H, m, H3), 1.37 (9H, s, H15), 1.05–1.29 (1H, m, H5); **^{13}C NMR + DEPT 135** (100MHz, DMSO-d_6, 298K) δ (ppm) 153.7, 144.9 (C, C8, C13), 132.2 (C, C11), 130.2, 127.5 (CH, C9, C10),

78.8 (C, C14), 70.0 (CH$_2$, C7), 66.6 (CH, C4), 43.9 (CH$_2$, C2), 43.1 (CH, C3), C6 not visible, 33.7 (CH$_2$, C5), 27.7 (CH$_3$, C15), 20.7 (CH$_3$, C12).

***Cis-N*-Boc-4-hydroxy-3-(tosyloxymethyl)piperidine**

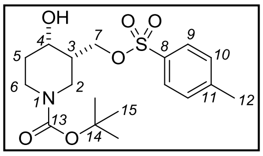

^{1}H NMR (400MHz, CDCl$_3$) δ (ppm) 7.80 (2H, d, *J* 8.2 Hz, H9 or H10), 7.36 (2H, d, *J* 8.1 Hz, H9 or H10), 4.04–4.23 (2H, m, H7, H4), 3.07–4.02 (4H, m, H4, H2, H6), 2.46 (3H, s, H12), 2.03 (1H, m, H3), 1.57–1.75 (3H, m, H5, H2 or H6), 1.45 (9H, s, H15); **^{13}C NMR + DEPT 135** (100MHz, CDCl$_3$) δ (ppm) 154.8, 145.0 (C, C8, C13), C11 not visible, 130.0, 127.9 (CH, C9, C10), 79.8 (C, C14), 68.8 (CH$_2$, C7), 40.2 (CH, C3), 38.7 (CH$_2$, C2 or C6), C2 or C6 not visible, 32.1 (CH$_2$, C5), 28.4 (CH$_3$, C15), 21.7 (CH$_3$, C12); **^{1}H NMR** (400MHz, DMSO-d_6, 298K) δ (ppm) 7.73–7.86 (2H, m, H9 or H10), 7.49 (2H, d, *J* 7.8 Hz, H9 or H10), 4.85 (1H, br. s., OH), 3.98–4.12 (1H, m, H7), 3.72–3.94 (2H, m, H7, H4), 3.33–3.67 (2H, m, H2, H6), 2.97–3.29 (2H, m, H2, H6), 2.42 (3H, s, H12), 1.84 (1H, br. s., H3), 1.48 (1H, m, 2 H5), 1.37 (16H, s, H15); **^{13}C NMR + DEPT 135** (100MHz, DMSO-d_6, 298K) δ (ppm) 153.9, 144.9 (C, C8, C13), 132.3 (C, C11), 130.2, 127.5 (CH, C9, C10), 78.6 (C, C14), C7, C4, C2, C3, C6 and C5 not visible, 27.9 (CH$_3$, C15), 21.0 (CH$_3$, C12).

3. *N*-Boc-3-((3,4-(methylenedioxy)phenoxy)methyl)piperidin-4-ol (10)

***Trans-N*-Boc-3-((3,4-(methylenedioxy)phenoxy)methyl)piperidin-4-ol**

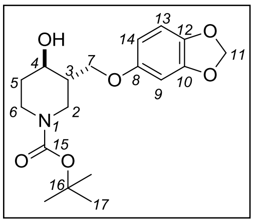

^{1}H NMR (400MHz, CDCl$_3$) δ (ppm) 6.70 (1H, d, *J* 8.5 Hz, H13), 6.50 (1H, d, *J* 2.5 Hz, H9), 6.34 (1H, dd, *J* 8.5, 2.5 Hz, H14), 5.92 (2H, s, H11), 3.88–4.33 (4H, m, H2, H6, 2 H7), 3.65–3.87 (1H, m, H4), 2.54–2.93 (2H, m, H2, H6), 1.80–2.03 (2H, m, H3, H5), 1.36–1.59 (10H, m, H17, H5); **^{13}C NMR + DEPT 135** (100MHz, CDCl$_3$) δ (ppm) 154.7, 154.1, 148.3, 142.0 (C, C15, C8, C10, C12), 107.9 (CH, C13), 105.8 (CH, C14), 101.2 (CH$_2$, C11), 98.1 (CH, C9), 79.8 (C, C16), 70.7 (CH, C4), 69.2 (CH$_2$, C7), 44.4 (CH$_2$, C2 or C6), 43.4 (CH, C3), ~40 (C2 or C6, not visible), 33.8 (CH$_2$, C5), 28.4 (CH$_3$, C17); **^{1}H NMR** (400MHz, DMSO-d_6, 298K) δ (ppm) 6.81 (1H, d, *J* 8.6 Hz, H13), 6.61 (1H, d, *J* 2.4 Hz, H9), 6.37 (1H, dd, *J* 8.5, 2.5 Hz, H14), 5.95 (2H, s, H11), 4.87 (1H, d, *J* 5.4 Hz, OH), 3.87–4.18 (2H, m, H7, H2), 3.71–3.87 (2H, m, H6, H7), 3.46 (1H, m, H4), 2.69–2.95 (2H, m, H6, H2), 1.59–1.88 (2H, m, H5, H3), 1.17–1.47 (10H, m, 9 H17, H5); **^{13}C NMR + DEPT 135** (100MHz, DMSO-d_6, 298K) δ (ppm) 154.1, 153.9, 147.9, 141.1 (C, C15, C8, C10, C12), 108.0 (CH, C13), 105.7 (CH, C14), 100.9 (CH$_2$, C11), 97.8 (CH, C9), 78.6 (C, C16), 67.7 (CH$_2$, C7), 67.0 (CH, C4), 44.4 (CH$_2$, C2), 43.4 (CH, C3), C6 and C5 not visible, 28.0 (CH$_3$, C17).

***Cis-N*-Boc-3-((3,4-(methylenedioxy)phenoxy)methyl)piperidin-4-ol**

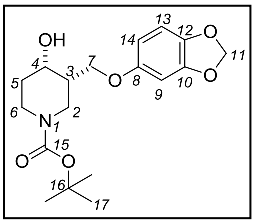

^{1}H NMR (400MHz, CDCl$_3$) δ (ppm) 6.70 (1H, d, *J* 8.5 Hz, H13), 6.49 (1H, d, *J* 2.5 Hz, H9), 6.33 (1H, dd, *J* 8.5, 2.5 Hz, H14), 5.91 (2H, s, H11), 4.21 (1H, br. s., H4), 3.86–4.11 (2H, m, H7), 3.04–3.85 (4H, m, H2 and H6), 2.18–2.45 (1H, m, OH), 2.02–2.18 (1H, m, H3), 1.73 (2H, br. d, *J* 4.3 Hz, H5), 1.45 (9H, s, H17); **^{13}C NMR + DEPT 135** (100MHz, CDCl$_3$) δ (ppm) 155.0, 154.0, 148.3, 141.9 (4 C, C15, C8, C10, C12), 107.9 (CH, C13), 105.8 (CH, C14), 101.2 (CH$_2$, C11), 98.1 (CH, C9), 79.6 (C, C16), 68.8 (CH$_2$, C7), 66.5 (CH, C4), 40.3 (CH, C3), 39.0 (CH$_2$, C2 and C6), 32.2 (CH$_2$, C5), 28.4 (CH$_3$, C17); **^{1}H NMR** (400MHz, DMSO-d_6, 298K) δ (ppm) 6.79 (1H, d, *J* 8.5 Hz, H13), 6.60 (1H, d, *J* 2.4 Hz, H9), 6.35 (1H, dd, *J* 8.5, 2.5 Hz, H14), 5.94 (2H, s, H11), 4.82 (1H, br. s., OH), 3.83–4.00 (2H, m, H7, H4), 3.71 (1H, app. t, *J* 9.2 Hz, H7), 1.84–2.06 (2H, m, H3), 1.47–1.62 (2H, m, H5), 1.34 (9H, br. s., H17); **^{13}C NMR + DEPT 135** (100 MHz, DMSO-d_6, 298K) δ (ppm) 154.1, 154.0, 147.9, 141.0 (C, C15, C8, C10, C12), 108.0 (CH, C13), 105.7 (CH, C14), 100.9 (CH$_2$, C11), 97.7 (CH, C9), 78.4 (C, C16), 40.3 (CH, C3), 39.1 (CH$_2$, C6 or C2), 27.9 (CH$_3$, C17).

4. *N*-Boc-4-bromo-3-((3,4-(methylenedioxy)phenoxy)methyl)piperidine (6)

***Trans-N*-Boc-4-bromo-3-((3,4-(methylenedioxy)phenoxy)methyl)piperidine**

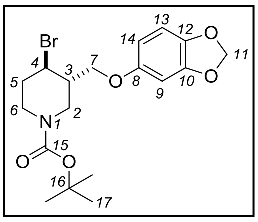

^{1}H NMR (400MHz, CDCl$_3$) δ (ppm) 6.70 (1H, d, *J* 8.6 Hz, H13), 6.50 (1H, d, *J* 2.0 Hz, H9), 6.34 (1H, dd, *J* 8.6, 2.5 Hz, H14), 5.92 (2H, s, H11), 3.65–4.39 (5H, m, H4, H2, 2 H7, H6), 2.89–3.15 (2H, m, H2, H6), 2.31 (1H, m, H5), 2.18 (1H, m, H3), 2.03 (1H, m, H5), 1.46 (9H, s, H17); **^{13}C NMR + DEPT 135** (100MHz, CDCl$_3$) δ (ppm) 154.5, 154.1, 148.3, 141.9 (C, C15, C8, C10, C12), 107.9 (CH, C13), 105.9 (CH, C14), 101.1 (CH$_2$, C11), 98.2 (CH, C9), 80.0 (C, C16), 69.2 (CH$_2$, C7), 50.7 (CH, C4), 46.0 (CH$_2$, C2), 44.6 (CH, C3), 43.9 (CH$_2$, C6), 36.2 (CH$_2$, C5), 28.3 (CH$_3$, C17); **^{1}H NMR** (400MHz, DMSO-d_6, 298K) δ (ppm) 6.81 (1H, d, *J* 8.1 Hz, H13), 6.63 (1H, br. s, H9), 6.38 (1H, br. d, *J* 8.6 Hz, H14), 5.96 (2H, s, H11), 4.41 (1H, m, H4), 3.60–4.17 (4H, m, H2, H6, 2 H7), 3.03 (1H, m, H2 or H6), 2.27 (1H, br. d, *J* 12.6 Hz, H5), 2.08 (1H, m, H3), 1.87 (1H, m, H5), 1.38 (9H, s, H17); **^{13}C NMR + DEPT 135** (100MHz, DMSO-d_6, 298K) δ (ppm) 153.7, 147.9, 141.4

(C, C15, C8, C10, C12), 108.0 (CH, C13), 105.9 (CH, C14), 101.0 (CH_2, C11), 98.0 (CH, C9), 79.0 (C, C16), 68.9 (CH_2, C7), 52.1 (CH, C4), 45.4 (CH_2, C2), 44.2 (CH, C3), 27.9 (CH_3, C17).

***Cis-N*-Boc-4-bromo-3-((3,4-(methylenedioxy)phenoxy)methyl)piperidine**

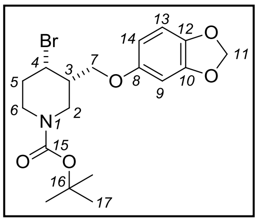

^{1}H NMR (400MHz, $CDCl_3$) δ (ppm) 6.70 (1H, d, *J* 8.6 Hz, H13), 6.50 (1H, d, *J* 2.5 Hz, H9), 6.34 (1H, dd, *J* 8.6, 2.5 Hz, H14), 5.92 (2H, s, H11), 4.76 (1H, br. s., H4), 3.69–4.22 (4H, m, H2, H6, 2 H7), 3.28 (1H, ddd, *J* 14.1, 10.1, 4.5 Hz, H6), 2.96 (1H, m, H2), 1.98–2.20 (3H, m, H3, H5, H5), 1.47 (9H, s, H17); **^{13}C NMR + DEPT 135** (100MHz, $CDCl_3$) δ (ppm) 154.7, 154.1, 148.3, 141.9 (C, C15, C8, C10, C12), 107.9 (CH, C13), 105.9 (CH, C14), 101.2 (CH_2, C11), 98.3 (CH, C9), 79.9 (C, C16), 69.6 (CH_2, C7), 54.1 (CH, C4), 40.8 (CH, C3), 39.4 (CH_2, C6), 33.8 (CH_2, C5), 28.4 (CH_3, C17); **^{1}H NMR** (400MHz, DMSO-d_6, 298K) δ (ppm) 6.80 (1H, d, *J* 8.6 Hz, H13), 6.63 (1H, d, *J* 2.5 Hz, H9), 6.38 (1H, dd, *J* 8.6, 2.5 Hz, H14), 5.95 (2H, s, H11), 4.86 (1H, m, H4), 3.58–4.10 (4H, m, H2, 2 H7, H6), 3.17 (1H, m, H2 or H6), 1.92–2.17 (3H, m, H3, 2 H5), 1.37 (9H, s, H17); **^{13}C NMR + DEPT 135** (100MHz, DMSO-d_6, 298K) δ (ppm) 153.6, 153.5, 147.6, 141.2 (C, C15, C8, C10, C12), 107.6 (CH, C13), 106.1 (CH, C14), 100.6 (CH_2, C11), 97.8 (CH, C9), 78.6 (C, C16), 69.1 (CH_2, C7), 55.1 (CH_2, C4), 41.5 (CH_2, C2 or C6), 39.8 (CH, C3), 39.4 (CH_2, C2 or C6), 33.0 (CH_2, C5), 27.7 (CH_3, C17).

5. *N*-Boc-4-(*p*-fluorophenyl)-3-[3,4-(methylenedioxy)phenoxymethyl]piperidine (11)

***Trans-N*-Boc-4-(*p*-fluorophenyl)-3-[3,4-(methylenedioxy)phenoxymethyl]piperidine**

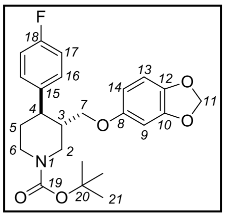

^{1}H NMR (300MHz, $CDCl_3$) δ (ppm) 7.08–7.22 (2H, m), 6.98 (2H, br. t, *J* 8.8 Hz), 6.63 (1H, d, *J* 8.4 Hz, H13), 6.36 (1H, d, *J* 2.6 Hz, H9), 6.14 (1H, dd, *J* 8.4, 2.6 Hz, H14), 5.89 (2H, s, H11), 4.35–4.58 (1H, m), 4.12–4.34 (1H, m), 3.61 (1H, dd, *J* 9.3, 2.7 Hz), 3.45 (1H, dd, *J* 9.5, 6.6 Hz), 2.74–2.96 (2H, m), 2.68 (1H, td, *J* 11.5, 4.0 Hz), 1.93–2.09 (1H, m), 1.61–1.87 (2H, m), 1.51 (9H, s, H21); **^{13}C NMR + DEPT 135** (75MHz, $CDCl_3$) δ (ppm) 161.6 (C, d, *J* 244.4 Hz, C18), 154.8, 154.2, 148.1, 141.1 (C, C19, C8, C10, C12), 139.1 (C, d, *J* 2.2 Hz, C15), 128.7 (CH, d, *J* 7.7 Hz, C16), 115.5 (CH, d, *J* 21.0 Hz, C17), 107.8 (CH_{Ar}), 105.5 (CH_{Ar}), 101.1 (CH_2, C11), 98.0 (CH_{Ar}), 79.7 (C, C20), 68.8 (CH_2, C7), C2 and C6 not visible, 44.0, 41.9 (CH, C3, C4), 33.9 (CH_2, C5), 28.5 (CH_3, C21); **^{19}F NMR** (282MHz, $CDCl_3$) δ (ppm) -116.4 (1F, s, F18). Spectra consistent with reported data.[3] **^{1}H NMR** (400MHz, DMSO-d_6, 298K) δ (ppm) 7.29 (2H, dd, *J* 8.6, 5.6 Hz, H16), 7.10 (2H, t, *J* 8.8 Hz, H17), 6.72 (1H, d, *J* 8.6 Hz, H13), 6.44 (1H, d, *J* 2.5 Hz, H9), 6.16 (1H, dd, *J* 8.3, 2.3 Hz, H14), 5.92 (2H, s, H11), 4.28 (1H, br. s., H2), 4.05 (1H, br. d, *J* 12.6 Hz, H6), 3.43–3.59 (2H, m, H7), 2.60–2.96 (3H, m, H2, H6, H4), 2.00 (1H, m, H3), 1.50–1.77 (2H, m, H5), 1.42 (9H, s, H21). **^{19}F NMR** (282MHz, DMSO-d_6, 298K) δ (ppm) -116.2 (1F, s, F18).

***Cis-N*-Boc-4-(*p*-fluorophenyl)-3-[3,4-(methylenedioxy)phenoxymethyl]piperidine**

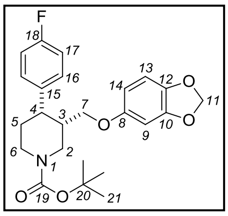

R_f 0.08 (pentane/acetone 94:6). **R_f** 0.08 (pentane/acetone 94:6); **IR (neat)** 2981 (w), 2929 (w), 1686 (br. s), 1489 (m), 1242 (br. m), 1183 (br. s), 1038 (m) cm^{-1}; **^{1}H NMR** (400MHz, $CDCl_3$) δ (ppm) 7.13–7.23 (2H, m, H16), 7.03 (2H, br. t, *J* 9.1 Hz, H17), 6.61 (1H, d, *J* 8.6 Hz, H13), 6.31 (1H, d, *J* 2.5 Hz, H9), 6.11 (1H, dd, *J* 8.3, 2.3 Hz, H14), 5.87 (2H, s, H11), 4.28–4.61 (2H, m, H2, H6), 3.77 (1H, t, *J* 9.6 Hz, H7), 3.42 (1H, m, H7), 2.95–3.15 (2H, m, H4, H2), 2.83 (1H, m, H6), 2.31 (1H, m, H3), 1.95 (1H, qd, *J* 12.8, 4.5 Hz, H5), 1.76 (1H, br. dd, *J* 13.1, 2.5 Hz, H5), 1.33 (9H, br. s., H21); **^{13}C NMR + DEPT 135** (100 MHz, $CDCl_3$) δ (ppm) 161.5 (C, d, *J* 244.4 Hz, C18), 155.1, 154.2, 148.1, 141.5, 138.4 (C, C19, C8, C10, C12, C15), 128.5 (CH, d, *J* 8.8 Hz, C16), 115.3 (CH, d, *J* 22.0 Hz, C17), 107.8 (CH, C13), 105.2 (CH, C14), 101.0 (CH_2, C11), 97.8 (CH, C9), 79.6 (C, C20), 64.0 (CH_2, C7), 45.9 (CH_2, C2), 43.6 (CH_2, C6), 42.6 (CH, C4), 40.2 (CH, C3), 28.2 (CH_3, C21), 25.5 (CH_2, C5); **^{19}F NMR** (282MHz, $CDCl_3$) δ (ppm) -116.6 (1F, s, F18); **^{1}H NMR** (400MHz, DMSO-d_6, 298K) δ (ppm) 7.34 (2H, dd, *J* 8.6, 5.6 Hz, H16), 7.14 (2H, t, *J* 9.1 Hz, H17), 6.72 (1H, d, *J* 8.6 Hz, H13), 6.46 (1H, br. s., H9), 6.20 (1H, br. d, *J* 5.6 Hz, H14), 5.90 (2H, s, H11), 4.07–4.37 (2H, m, H2, H4), 3.76 (1H, t, *J* 9.9 Hz, H7), 3.19–3.29 (1H, m), 2.70–3.18 (3H, m), 2.22 (1H, m, H3), 1.87–2.07 (1H, m, H5), 1.52–1.69 (1H, m, H5), 1.19 (9H, br. s., H21); **^{19}F NMR** (282MHz, DMSO-d_6, 298K) δ (ppm) -116.5 (1F, s, F18); **^{1}H NMR** (400MHz, DMSO-d_6, 353K) δ (ppm) 7.33 (2H, dd, *J* 8.1, 5.6 Hz, H16), 7.11 (2H, t, *J* 8.8 Hz, H17), 6.68 (1H, d, *J* 8.1 Hz, H13), 6.38 (1H, d, *J* 2.5 Hz, H9), 6.16 (1H, dd, *J* 8.6, 2.5 Hz, H14), 5.89 (2H, s, H11), 4.31 (1H, dt, *J* 13.1, 2.0 Hz, $H2_{eq}$), 4.21 (1H, dt, *J* 13.1, 2.0 Hz, $H4_{ax}$), 3.78 (1H, t, *J* 9.6 Hz, H7), 3.38 (1H, dd, *J* 9.6, 2.5 Hz, H7), 3.13 (1H, dt, *J* 13.1, 4.0 Hz, H2), 3.01–3.09 (1H, m, $H6_{eq}$, under H_2O peak), 2.86 (1H, td, *J* 12.6, 3.0 Hz, $H6_{ax}$), 2.25 (1H, dd, *J* 6.3, 3.3 Hz, $H3_{eq}$), 1.96 (1H, qd, *J* 12.8, 4.5 Hz, $H5_{ax}$), 1.66 (1H, dq, *J* 13.1, 3.0 Hz, $H5_{eq}$), 1.31 (9H, s, H21); **^{13}C NMR + DEPT 135** (100 MHz, DMSO-d_6, 353K) δ (ppm) 160.5 (C, d, *J* 241.0 Hz, C18), 153.9, 153.8, 147.5, 140.8 (4C, C19, C8, C10, C12), 138.7 (C, d, *J* 2.9 Hz, C15), 128.7 (CH, d, *J* 7.8 Hz, C16), 114.4 (CH, d, *J* 20.4 Hz, C17), 107.4 (CH, C13), 105.5 (CH, C14), 100.5 (CH_2, C11), 97.3 (CH, C9), 78.1 (C, C20), 64.6 (CH_2, C7), 44.9 (CH_2, C2), 43.0 (CH_2, C6), 41.5 (CH, C4), 39.5 (CH, C3), 27.6 (CH_3, C21), 24.8 (CH_2, C5); **ES^+MS** m/z 430.0 $(M+H)^+$; **HRMS** (ES^+) for $C_{24}H_{28}FNNaO_5$ $(M+Na)^+$ calcd. 452.1844, found. 452.1850.

6. 1-bromo-2-[3,4-(methylenedioxy)phenoxymethyl]-4-(tert-butyl)cyclohexane (*cis*-13)

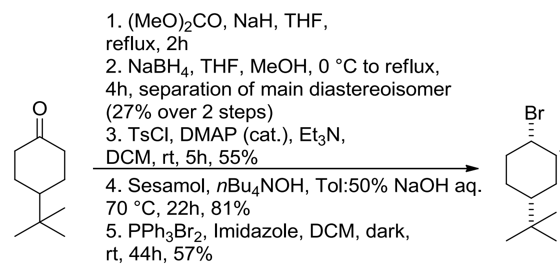

***cis*-13**

Following the method of Renaud,[5] to a suspension of sodium hydride (60% in mineral oil, 4.019 g, 100.5 mmol, 3.1 equiv.) washed with distilled pentane (2 x 50 mL) was added anhydrous THF (41.5 mL) and then dimethylcarbonate (6.83 mL, 81.0 mmol, 2.5 equiv.). 2.5 mL of a solution of 4-*tert*-butylcyclohexanone (5.000 g, 32.4 mmol, 1 equiv.) in THF (10 mL) were added and the solution was heated to reflux until the beginning of the reaction (H_2 emission). Then, the rest of the solution was added slowly over the period of 50 min. The semi-solid mixture was stirred another hour until which point the reaction had precipitated suddenly. Then, the reaction was allowed to cool down to rt then cooled to 0 °C. Then 10% aq. AcOH (50 mL) was added slowly at 0 °C and the mixture was stirred at rt until all the solid had dissolved. The aqueous layer was extracted with AcOEt (4 x 50 mL). The combined organic layers were dried over sodium sulphate and the solvent removed under reduced pressure to give methyl 5-(*tert*-butyl)-2-oxocyclohexanecarboxylate as an orange oil (6.789 g, 99%) which was engaged directly in the next step.

Methanol (27 mL) was added dropwise at 0 °C over a period of 20 min to mixture of methyl 5-(*tert*-butyl)-2-oxocyclohexanecarboxylate (6.789 g, 32.0 mmol, 1 equiv.) and sodium borohydride (3.630 g, 95.9 mmol, 3 equiv.) in THF (138 mL). Stirring was continued at 0 °C for 20 min, rt for 2 h then at reflux for 1.5 h. After cooling to rt, 1 M HCl (40 mL) was added at 0 °C, the mixture was filtered, extracted with ether (5 x 175 mL) and the organic phases were combined, washed with brine (175 mL) and dried with sodium sulphate. The solvent was evaporated under reduced pressure. The crude mixture was purified by column chromatography (petroleum ether/acetone from 90:10 to 70:30) to give 4-(*tert*-butyl)-2-(hydroxymethyl)cyclohexanol as a mixture of diastereoisomers (3.323 g, 56%). Recrystallization from hexane/diethyl ether gave *trans*-4-(*tert*-butyl)-2-(hydroxymethyl) cyclohexanol as a single diastereoisomer (1.630 g, 27%), where the substituents on the cyclohexane ring are all in equatorial position.

To a solution of *trans*-4-(*tert*-butyl)-2-(hydroxymethyl) cyclohexanol (1.500 g, 8.1 mmol, 1 equiv.), dry triethylamine (1.24 mL, 8.9 mmol, 1.1 equiv.) and DMAP (98 mg, 0.8 mmol, 0.1 equiv.) in dry DCM (29 mL) was added a solution of *p*-toluenesulfonyl chloride (1.689 g, 8.9 mmol, 1.1 equiv.) in anhydrous DCM (13 mL) under argon at rt. The reaction mixture was stirred at rt for 5h then water (50 mL) was added and the product was extracted with DCM (3 x 50 mL). Combined organic layers were dried over magnesium sulphate then concentrated under reduced pressure. The crude mixture was purified by column chromatography (petroleum ether:EtOAc 80:20 then 70:30) to give *trans*-4-(*tert*-butyl)-2-(tosyloxymethyl)cyclohexanol as a colourless oil (1.675 g, 55%).

To a solution of *trans*-4-(*tert*-butyl)-2-(tosyloxymethyl)cyclohexanol (1.658 g, 4.9 mmol, 1 equiv.), tetra-*n*-butylammonium hydroxide (1.0 M in H_2O, 0.24 mL, 0.24 mmol, 0.05 equiv.) and sesamol (740 mg, 5.6 mmol, 1.1 equiv.) in toluene (18.7 mL) is added a 50% NaOH aqueous solution (9.3 mL). The reaction mixture is stirred at 70 °C. After 22 hours, the aqueous and organic phases are separated. The aqueous phase is extracted with EtOAc (3 x 70 mL) then the combined organic phases are dried with magnesium sulphate and concentrated under reduced pressure. The crude mixture was purified by column chromatography (petroleum ether:EtOAc 80:20) to give *trans*-4-(*tert*-butyl)-2-(((3,4-methylenedioxy)phenoxy)methyl) cyclohexanol as a yellow oil (1.202 g, 81%).

To a solution of *trans*-4-(*tert*-butyl)-2-(((3,4-methylenedioxy)phenoxy)methyl)cyclohexanol (498 mg, 1.6 mmol, 1 equiv.) in dry DCM (3.6 mL) was added imidazole (133 mg, 2.0 mmol, 1.2 equiv.). The resulting slurry was cooled to 0 °C. in an ice bath. Bromotriphenylphosphonum bromide (755 mg, 1.8 mmol, 1.1 equiv.) in dry DCM (4.0 mL) was stirred for 15 min at rt then added dropwise to the reaction mixture. The solution was stirred in the dark for 44 hours at rt then diluted with water (11.5 mL) and extracted with DCM (3 x 33 mL). The organic layer was dried over sodium sulphate and concentrated under reduced pressure. The crude mixture was purified by column chromatography (hexane:EtOAc 98:2) to give *cis*-1-bromo-4-(*tert*-butyl)-2-(((3,4-methylenedioxy)phenoxy) methyl)cyclohexane ***cis*-13** as a colourless oil (342 mg, 57%).

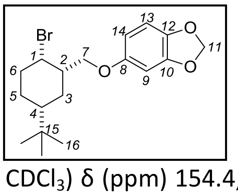

R_f 0.25 (hexane/EtOAc 98:2); **IR** (neat) 2947 (br. m), 1487 (s), 1184 (br. s), 1038 (m), 938 (m), 676 (w) cm^{-1}; **^{1}H NMR** (400MHz, $CDCl_3$) δ (ppm) 6.71 (1H, d, *J* 8.6 Hz, H13), 6.51 (1H, d, *J* 2.0 Hz, H9), 6.35 (1H, dd, *J* 8.3, 2.3 Hz, H14), 5.92 (2H, s, H11), 4.80 (1H, br. s., H1), 3.85 (1H, t, *J* 8.6 Hz, H7), 3.75 (1H, dd, *J* 9.1, 5.1 Hz, H7), 2.28 (1H, br. dd, *J* 14.7, 3.0 Hz, H6), 1.82–1.96 (2H, m, H2, H6), 1.57–1.71 (3H, m, H3, H5, H3 or H5), 1.10–1.32 (2H, m, H4, H3 or H5), 0.91 (9H, s, H16); **^{13}C NMR + DEPT 135** (100 MHz, $CDCl_3$) δ (ppm) 154.4, 148.2, 141.7 (C, C8, C10, C12), 107.9 (CH, C13), 106.0 (CH, C14), 101.1 (CH_2, C11), 98.3 (CH, C9), 72.9

(CH_2, C7), 57.8 (CH, C1), 47.1 (CH, C4), 42.7 (CH, C2), 35.0 (CH_2, C6), 32.6 (C, C15), 27.4 (CH_3, C16), 24.5, 21.7 (CH_2, C3, C5); **HRMS** (ES^+) for $C_{18}H_{26}{}^{79}BrO_3$ $(M+H)^+$ calcd. 369.1060, found. 369.1062.

7. 1-(4-fluorophenyl)-2-[3,4-(methylenedioxy)phenoxymethyl]-4-(tert-butyl) cyclohexane (*trans*-14)

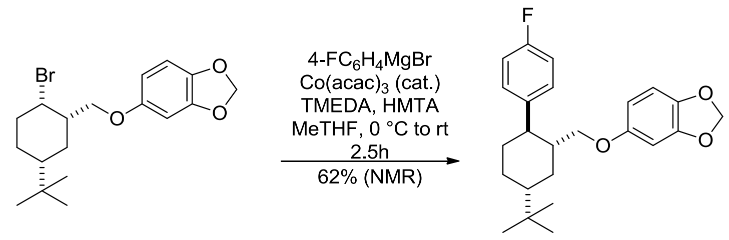

***cis*-13**

***trans*-14**
>95:5 *dr* (*trans*:*cis*)

To a flame dried 2 necks RBF were added 1-bromo-2-[3,4-(methylenedioxy)phenoxymethyl]-4-(tert-butyl)cyclohexane **13** (200 mg, 0.54 mmol, 1 equiv.), $Co(acac)_3$ (19 mg, 0.05 mmol, 0.1 equiv.), TMEDA (41 µL, 0.27 mmol, 0.5 equiv.), HMTA (38 mg, 0.27 mmol, 0.5 equiv.) and MeTHF (1.5 mL). The reaction mixture was cooled to 0 °C then a solution of 4-fluorophenylmagnesium bromide (1.0 M in MeTHF, 1.1 mL, 1.1 mmol, 2 equiv.) was added over 20 min. After completion of the addition, the reaction mixture was stirred for 1 h at 0 °C and 1 h at rt, then quenched with aqueous HCl 1M (2.2 mL). The aqueous layer was extracted with ether (3 x 8 mL) then the combined organic layers were dried with magnesium sulphate and concentrated under reduced pressure to give 1-(4-fluorophenyl)-2-[3,4-(methylenedioxy)phenoxymethyl]-4-(tert-butyl)cyclohexane **14** (62% conversion by ^{1}H NMR, >95:5 *dr* (*trans*:*cis*)). The crude mixture was purified by column chromatography (pentane:acetone 99:1) followed by HPLC (pentane:acetone 99:1) however ***trans*-14** was still obtained in mixture with remaining starting material.

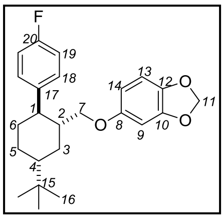

Rf 0.30 (pentane:acetone 99:1); **^{1}H NMR** (400MHz, $CDCl_3$) δ (ppm) 7.13 (2H, dd, *J* 8.6, 5.6 Hz, H18), 6.96 (2H, br. t, *J* 8.6 Hz, H19), 6.63 (1H, d, *J* 8.6 Hz, H13), 6.36 (1H, d, *J* 2.5 Hz, H9), 6.15 (1H, dd, *J* 8.6, 2.5 Hz, H14), 5.88 (2H, s, H11), 3.57 (1H, dd, *J* 8.8, 2.8 Hz, H7), 3.43 (1H, dd, *J* 9.1, 6.6 Hz, H7), 2.44 (1H, td, *J* 11.7, 3.3 Hz, H1), 2.12 (1H, br. dd, *J* 12.1, 2.5 Hz, H3), 1.81–2.00 (3H, m, H2, H5, H6), 1.43–1.72 (1H, m, H6), 1.05–1.34 (3H, m, H3, H4, H5), 0.93 (9H, s, H16); **^{13}C NMR + DEPT 135** (100MHz, $CDCl_3$) δ (ppm) 161.3 (C, d, *J* 244.4 Hz, C20), 154.7, 148.1, 141.4 (C, C8, C10, C12), 141.1 (C, d, *J* 2.9 Hz, C17), 128.7 (CH, d, *J* 7.3 Hz, C18), 115.2 (CH, d, *J* 22.0 Hz, C19), 107.8 (CH, C13), 105.6 (CH, C14), 101.0 (CH_2, C11), 98.0 (CH, C9), 71.9 (CH_2, C7), 47.7 (CH, C4), 45.8 (CH, C1), 43.5 (CH, C2), 35.7 (CH_2, C6), 32.5 (C, C15), 31.3 (CH_2, C3), 27.6 (CH_3, C16), 27.5 (CH_2, C5); **^{19}F NMR** (282MHz, $CDCl_3$) δ (ppm) -117.4 (1F, s, F20).

Confirmation of relative stereochemistry

Confirmation of the relative configuration of the different intermediates **9**, **10** and **6** was achieved by ^{1}H NMR studies. In the *trans* diastereoisomer, the $^{3}J_{H3\text{-}H4}$ value should be 10–13 Hz as both H3 and H4 are axial. However, in the *cis* diastereoisomer, one substituent must be in the equatorial position, hence $^{3}J_{H3\text{-}H4}$ will be around 2–5 Hz.

However, analysis proved difficult due to the rotamers arising from the presence of an *N*-boc protecting group. The NMR spectra in $CDCl_3$ at room temperature showed broad signals from which the coupling constants could not be determined. Therefore, high temperature NMR experiments were carried on our substrates in order to resolve the signals. These experiments require a high boiling NMR solvent, and therefore were carried out in DMSO-d_6. As an illustration, we show the confirmation of relative configuration for the monotosylate **9**. The signals of H4 and H3 can be resolved at high temperature.

For diastereoisomer **A**, H4 appeared to be a quadruplet with a coupling constant of 4.0 Hz and H3 came out as a triplet of doublets of triplets with coupling constants being 9.5, 5.2 and 3.8 Hz respectively. These coupling constants are in accordance with a *cis* configuration where H4 is equatorial and therefore couples with H3, $H5_{ax}$ and $H5_{eq}$ with a small coupling constant (4.0 Hz). H3 is axial and forms a triplet with H4 and $H2_{eq}$ with a small coupling constant (3.8 Hz). The triplet (9.5 Hz) and doublet (5.2 Hz) result from coupling with H7 and $H2_{ax}$.

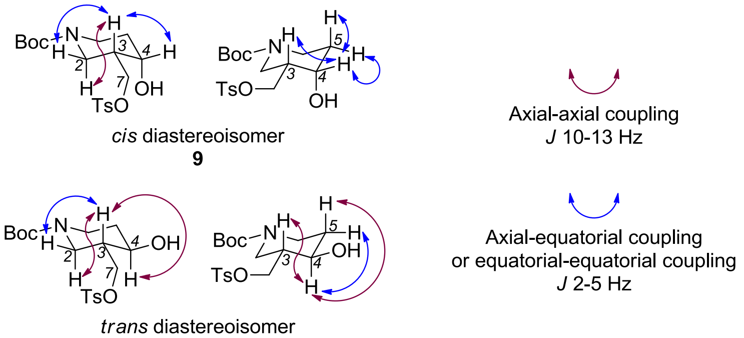

cis diastereoisomer
9

trans diastereoisomer
9

The signal for H4 in diastereoisomer **B** appeared to be a triplet of doublets (9.5 and 4.4 Hz respectively), which confirmed the *trans* configuration of this compound, where H3 and H4 are both in axial position. Indeed, the triplet with a large coupling constant of 9.5 Hz (axial-axial) results from the coupling with H3 and $H5_{ax}$. The doublet (4.4 Hz) is due to the coupling with $H5_{eq}$.

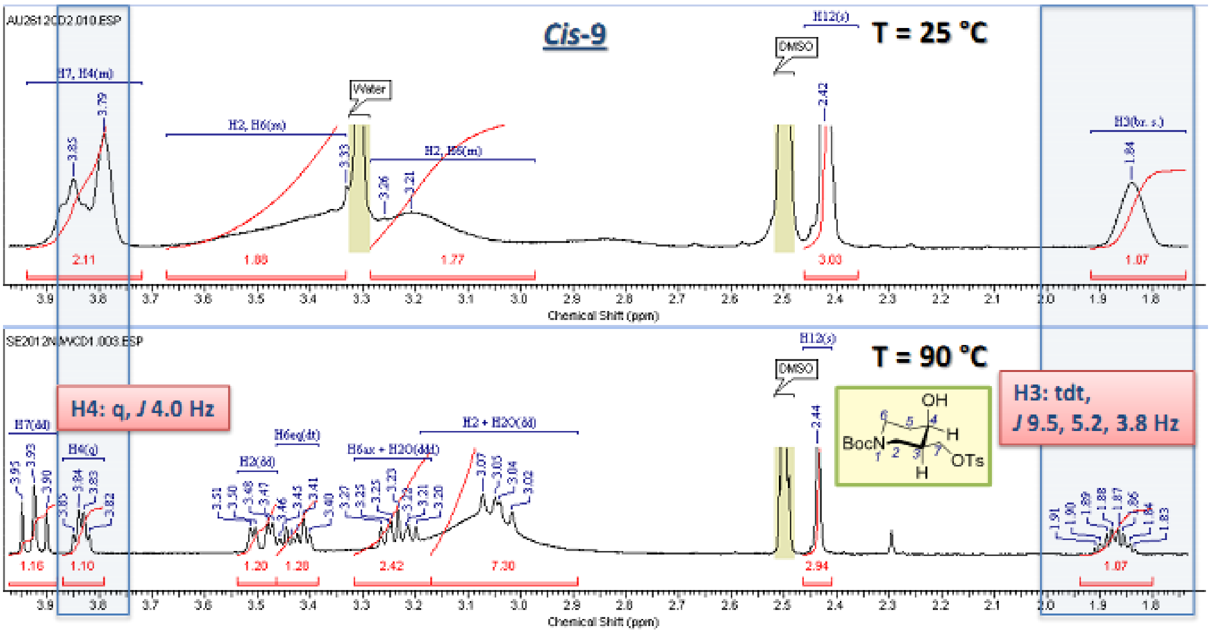

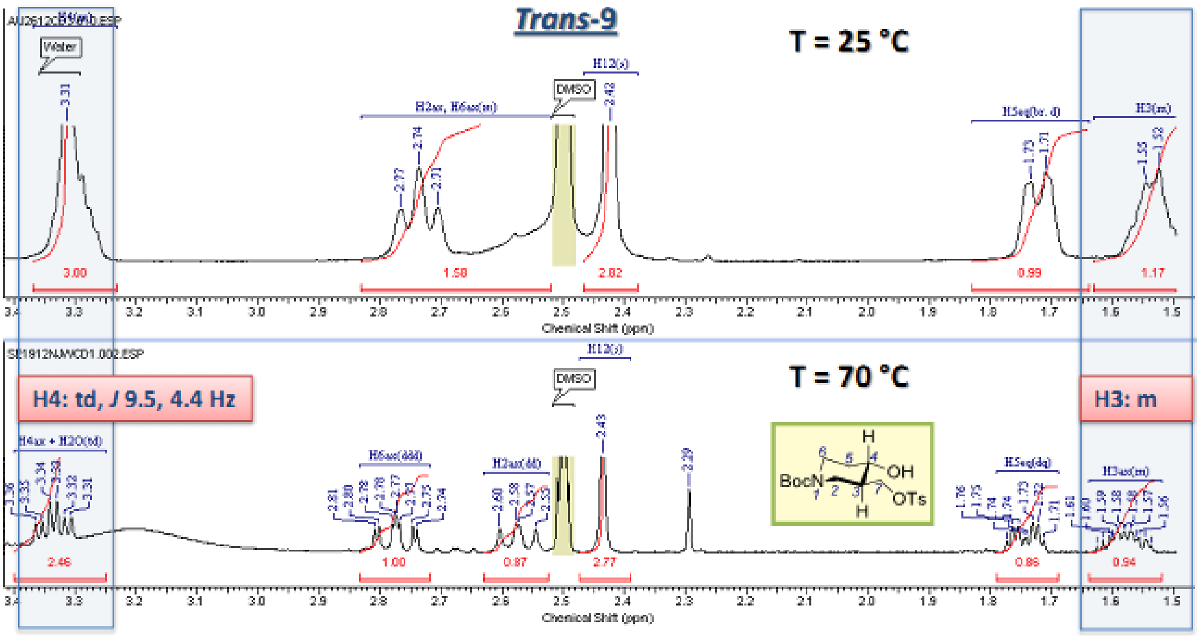

NMR spectra in DMSO-d_6 of the two diastereoisomers of **9**

Determination of diastereomeric ratio by crude ^{19}F NMR

1. **Table 1, entry 14**

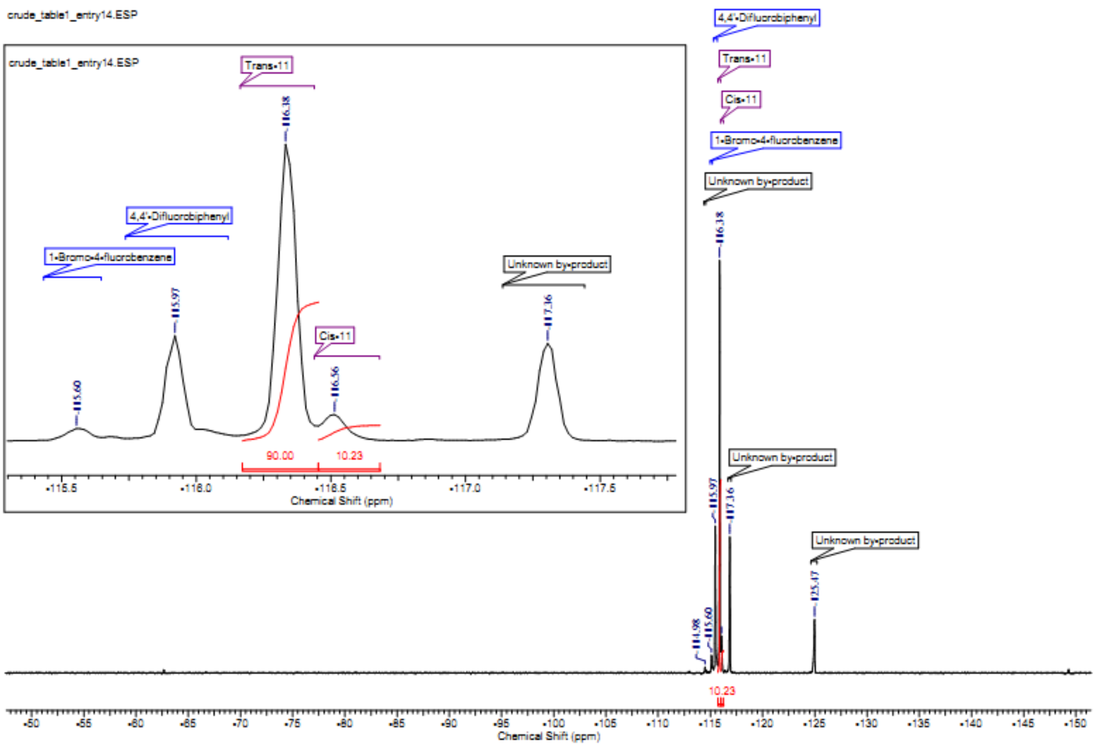

2. **Table 1, entry 18**

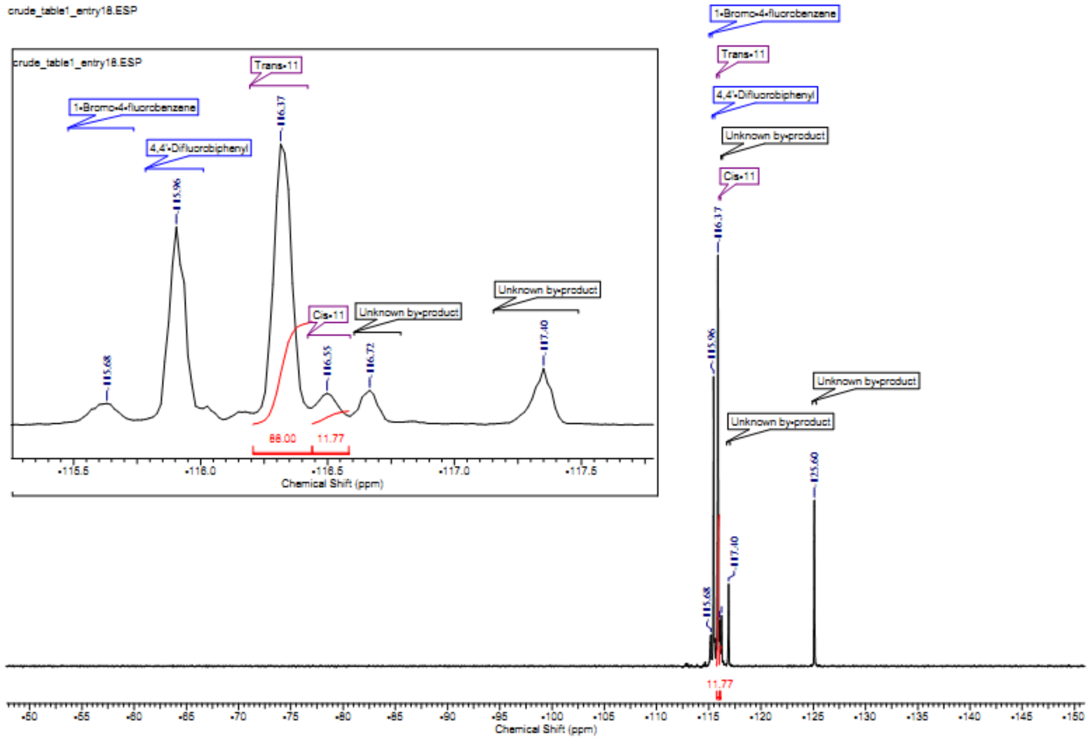

3. Scheme 4

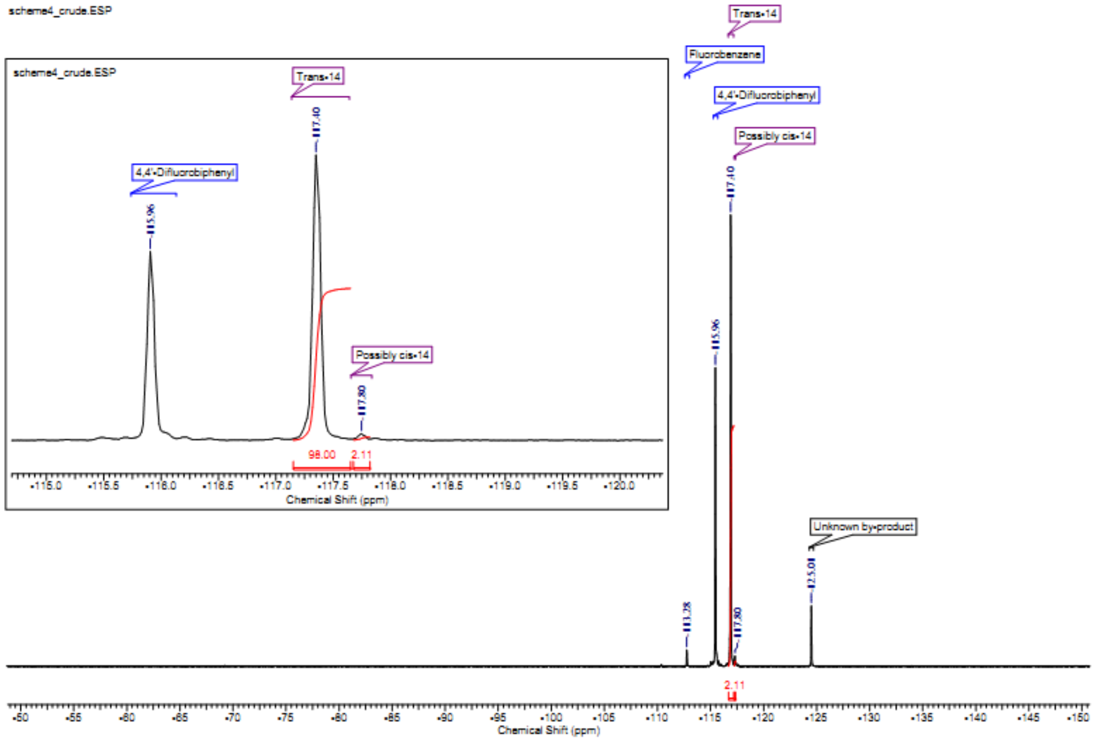

The signal around -125 ppm correspond to an unknown compound which is observed in every crude NMR spectra of the cross coupling reaction.

A proportion of fluorobenzene (-113.3 ppm), 4,4'-difluorobisphenyl (-116.0 ppm) and 1-bromo-4-fluorobenzene (-115.6 ppm) can already be found in the solution of Grignard reagent before the reaction.

NMR Spectra

4. *N*-Boc-3-hydroxymethyl-4-piperidol (7)

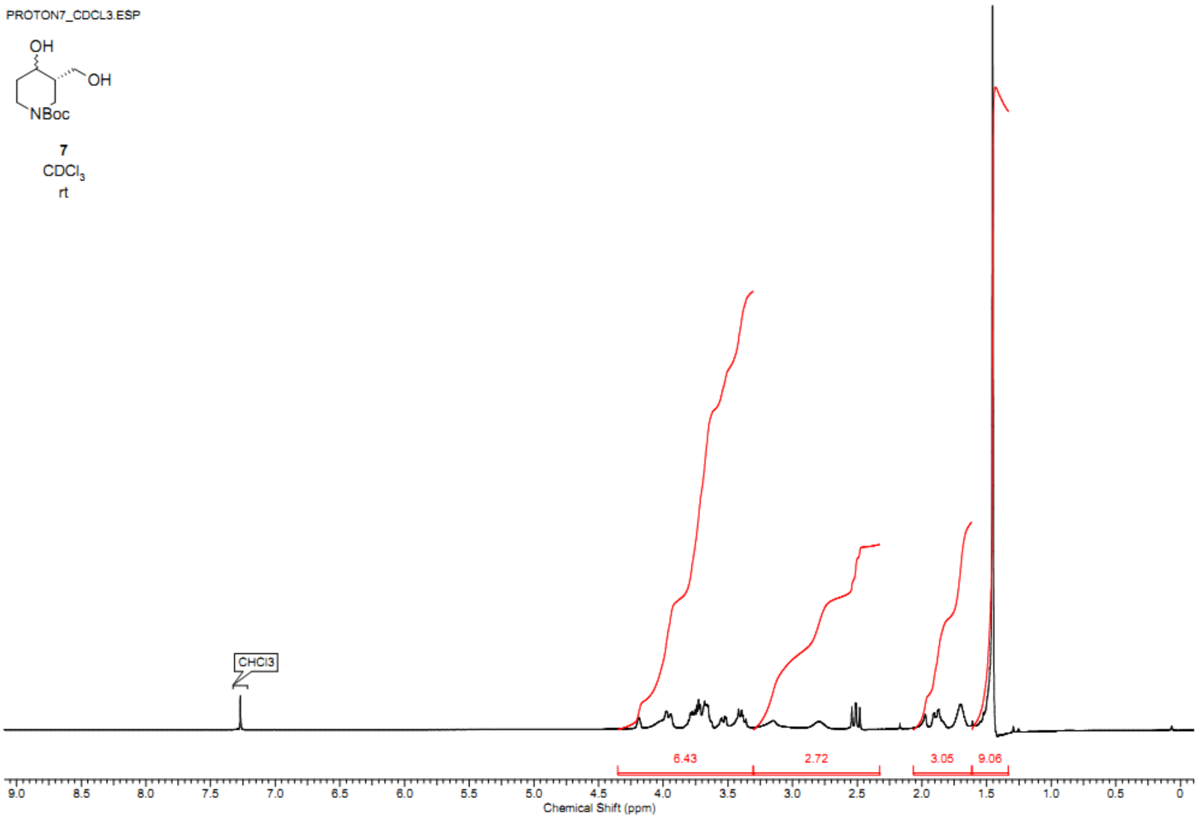

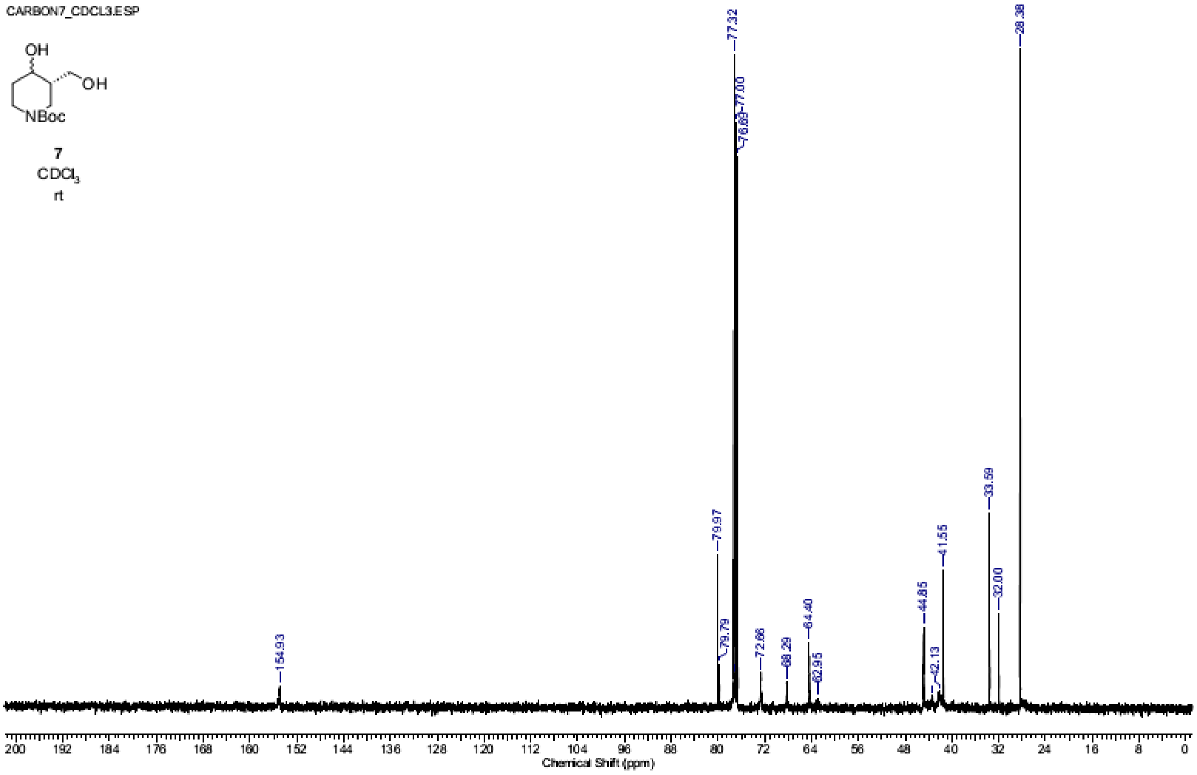

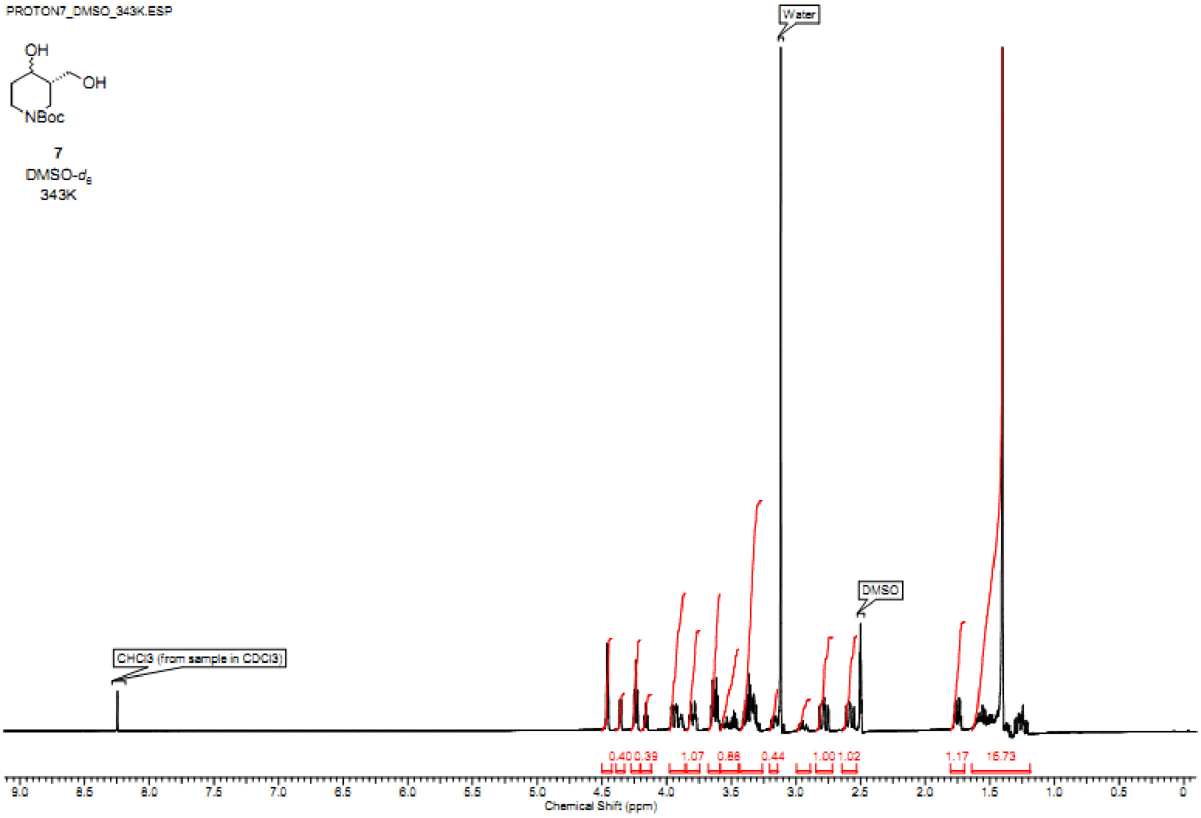
PROTON7_DMSO_343K.ESP
OH
OH
NBoc
7
DMSO-d_6
343K
Water
DMSO
CHCl3 (from sample in CDCl3)
0.40 0.39 1.07 0.88 0.44 1.00 1.02 1.17 16.73
Chemical Shift (ppm)

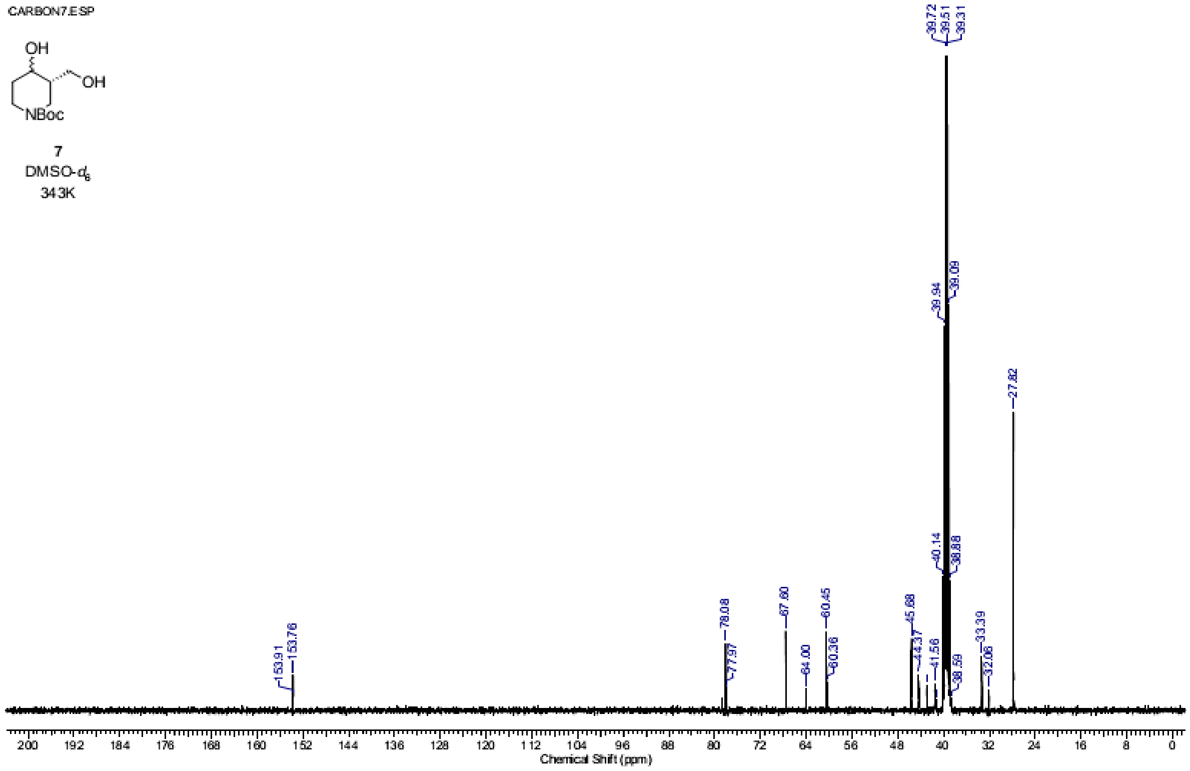
CARBON7.ESP
OH
OH
NBoc
7
DMSO-d_6
343K
39.72 39.51 39.31
39.94 39.09
27.82
40.14 38.88
78.08 77.97
67.60
60.45 60.36
64.00
45.68 44.37 41.56 38.59
33.39 32.06
153.91 153.76
Chemical Shift (ppm)

5. ***N*-Boc-4-hydroxy-3-(tosyloxymethyl)piperidine (9)**

Both diastereoisomers degrade at high temperature

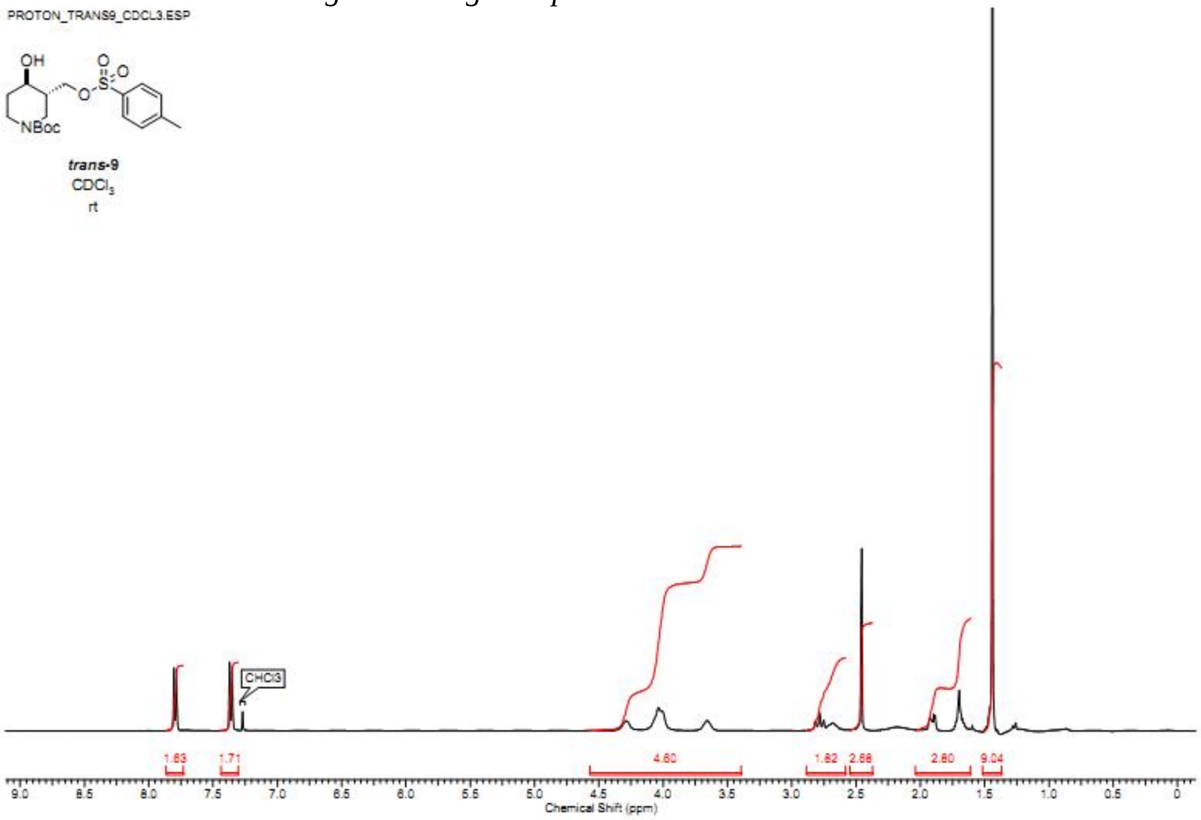

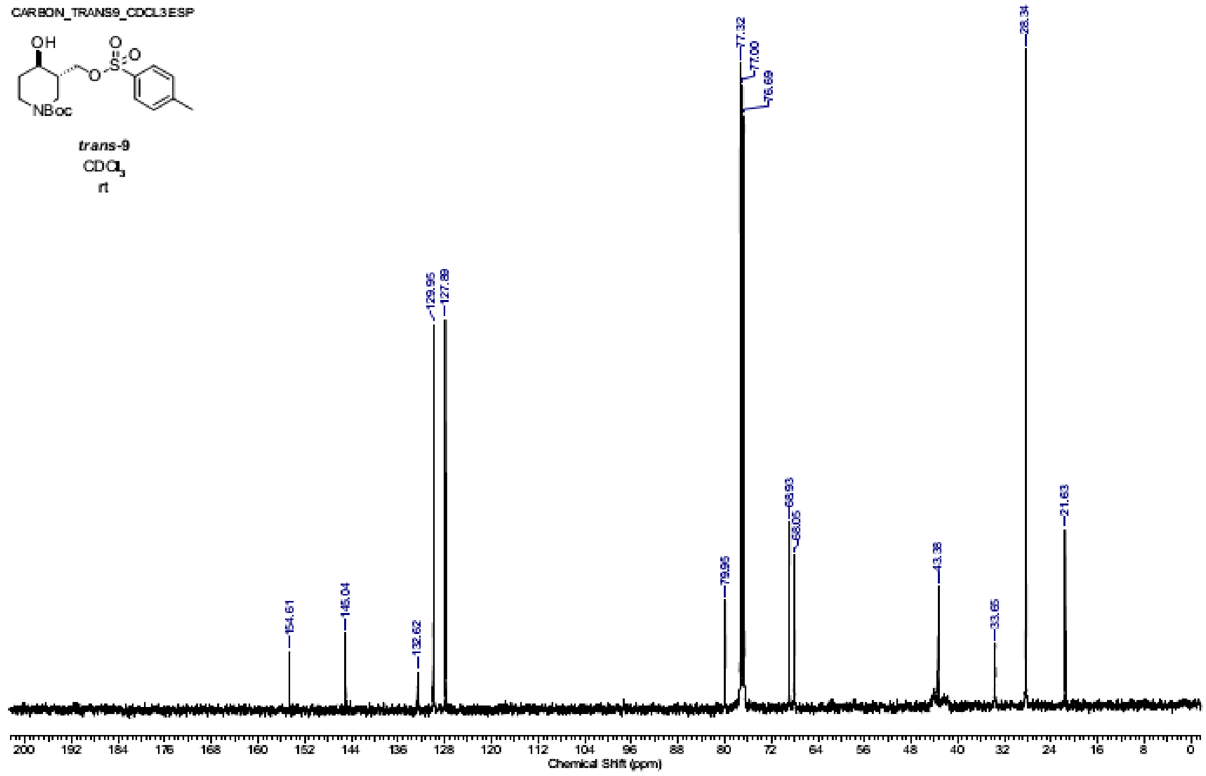

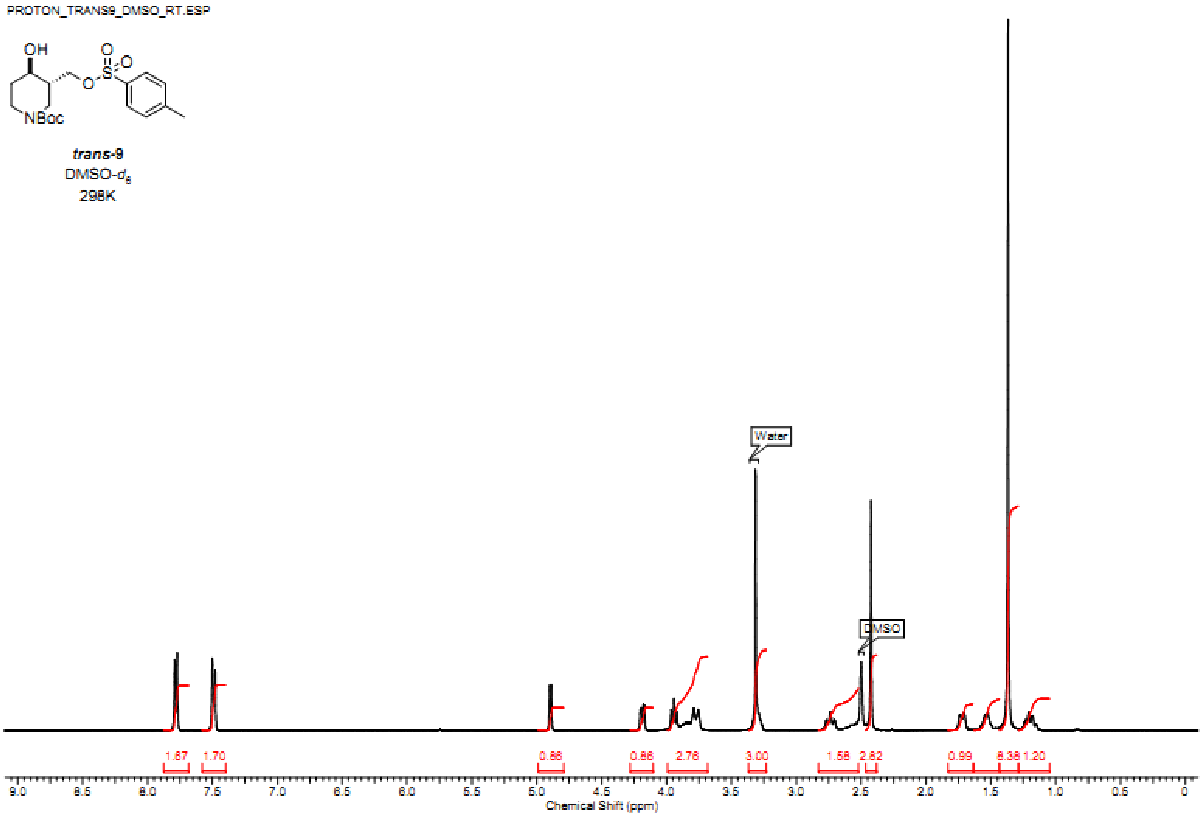
PROTON_TRANS9_DMSO_RT.ESP
OH
NBoc
trans-9
DMSO-d6
298K
Water
DMSO
1.87
1.70
0.88
0.86
2.76
3.00
1.58
2.82
0.99
8.38
1.20
Chemical Shift (ppm)

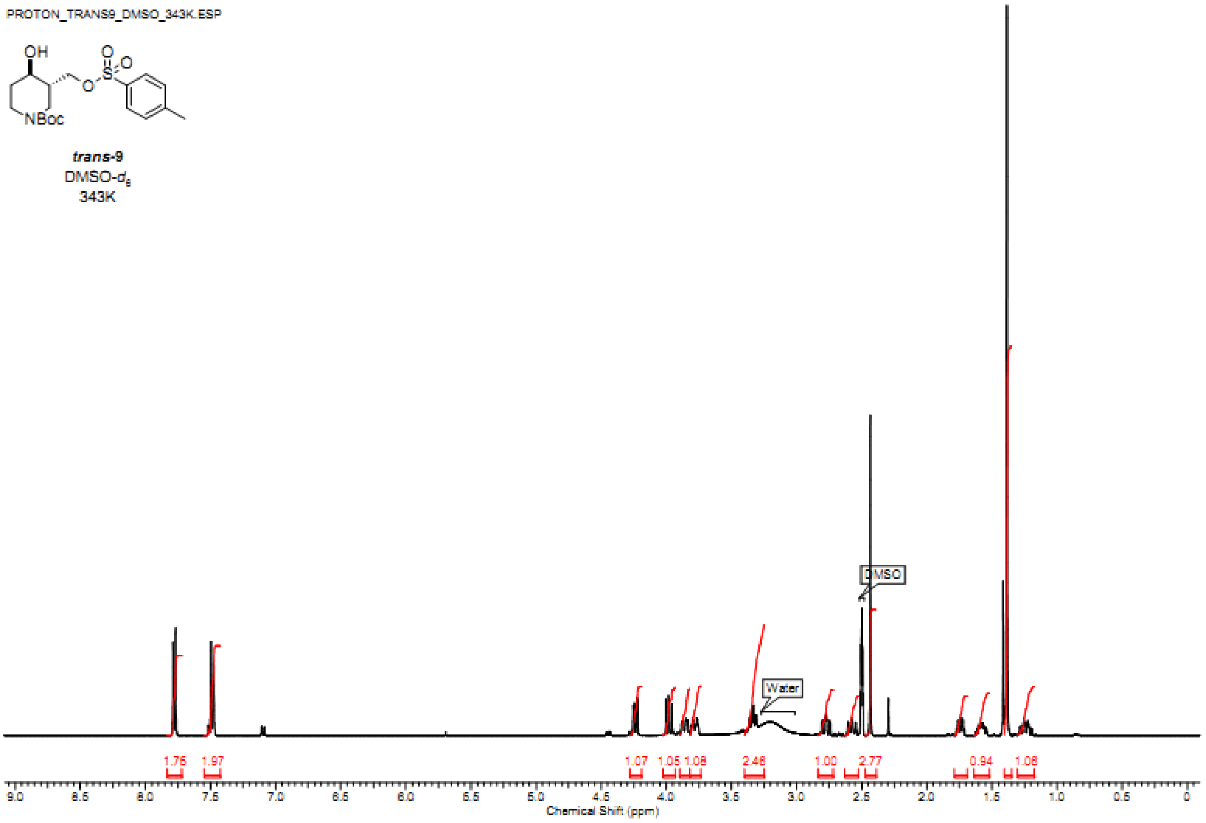
PROTON_TRANS9_DMSO_343K.ESP
OH
NBoc
trans-9
DMSO-d6
343K
DMSO
Water
1.76
1.97
1.07
1.05
1.08
2.46
1.00
2.77
0.94
1.08
Chemical Shift (ppm)

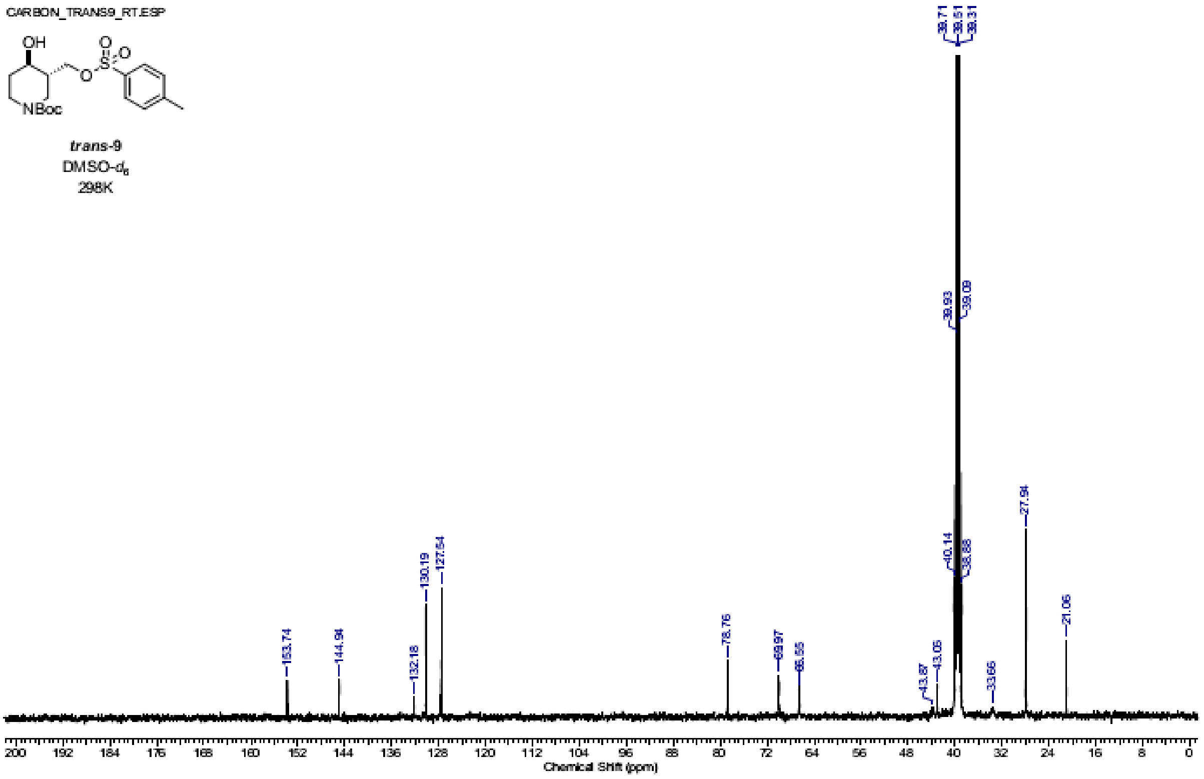
CARBON_TRANS9_RT.ESP
OH
O
S
O
O
NBoc
trans-9
DMSO-d6
298K
39.71
39.51
39.31
39.93
39.09
40.14
38.88
27.94
130.19
127.54
153.74
144.94
132.18
78.76
69.97
66.55
43.87
43.05
33.66
21.06
200 192 184 176 168 160 152 144 136 128 120 112 104 96 88 80 72 64 56 48 40 32 24 16 8 0
Chemical Shift (ppm)

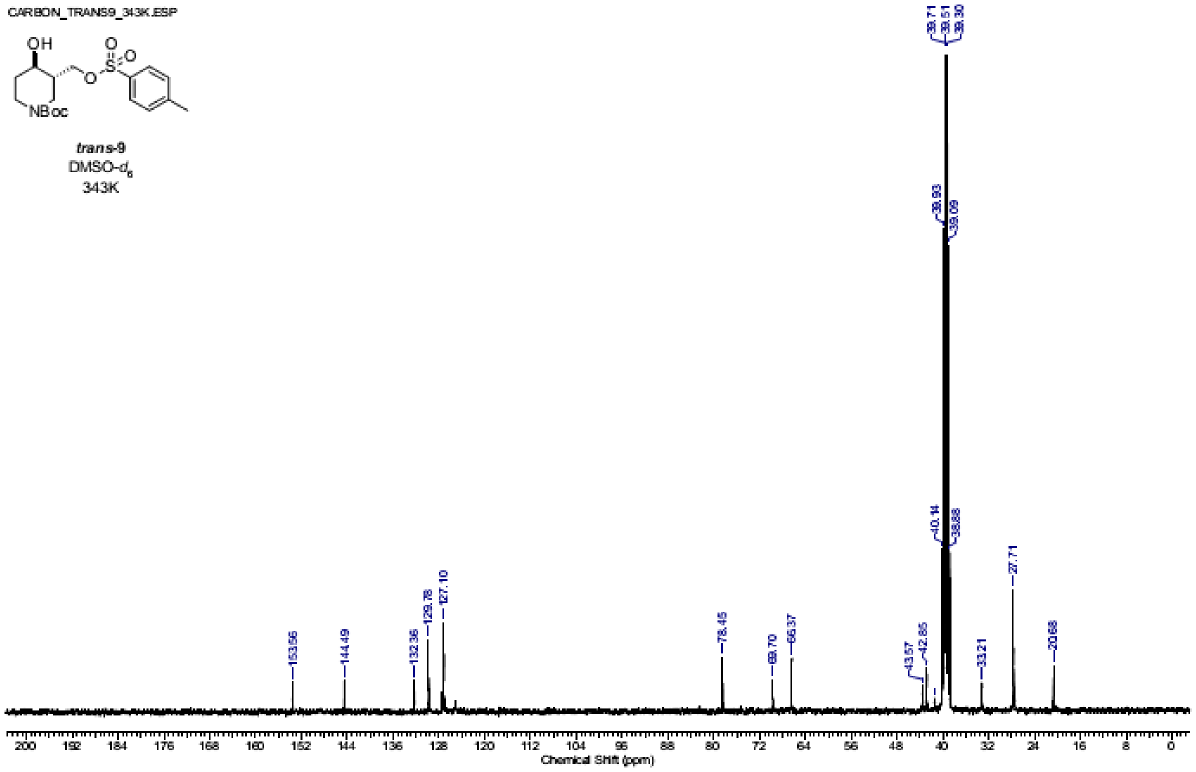
CARBON_TRANS9_343K.ESP
OH
O
S
O
O
NBoc
trans-9
DMSO-d6
343K
39.71
39.51
39.30
39.93
39.09
40.14
38.88
27.71
129.78
127.10
153.56
144.49
132.36
78.45
69.70
66.37
43.57
42.85
33.21
20.68
200 192 184 176 168 160 152 144 136 128 120 112 104 96 88 80 72 64 56 48 40 32 24 16 8 0
Chemical Shift (ppm)

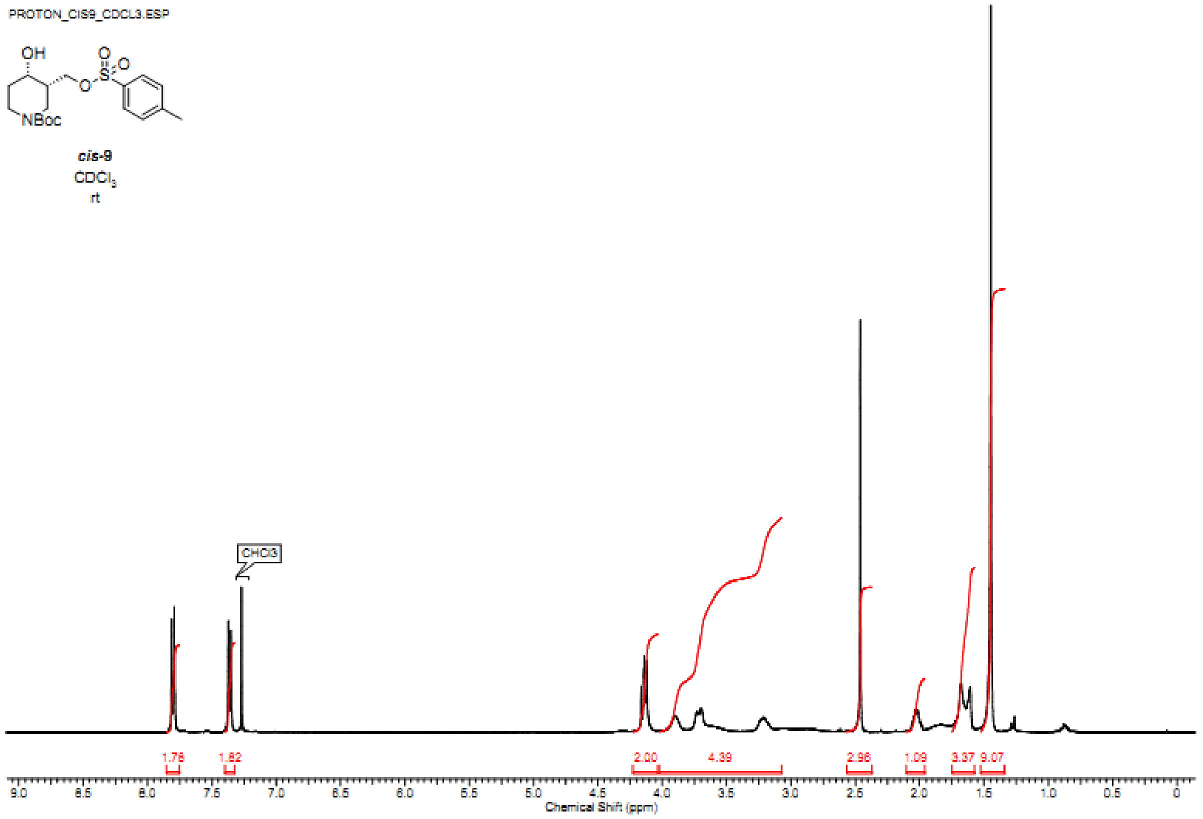

PROTON_CIS9_CDCL3.ESP
OH
O
O
S
O
NBoc
cis-9
CDCl3
rt
CHCl3
1.78
1.82
2.00
4.39
2.96
1.09
3.37
9.07
9.0
8.5
8.0
7.5
7.0
6.5
6.0
5.5
5.0
4.5
4.0
3.5
3.0
2.5
2.0
1.5
1.0
0.5
0
Chemical Shift (ppm)

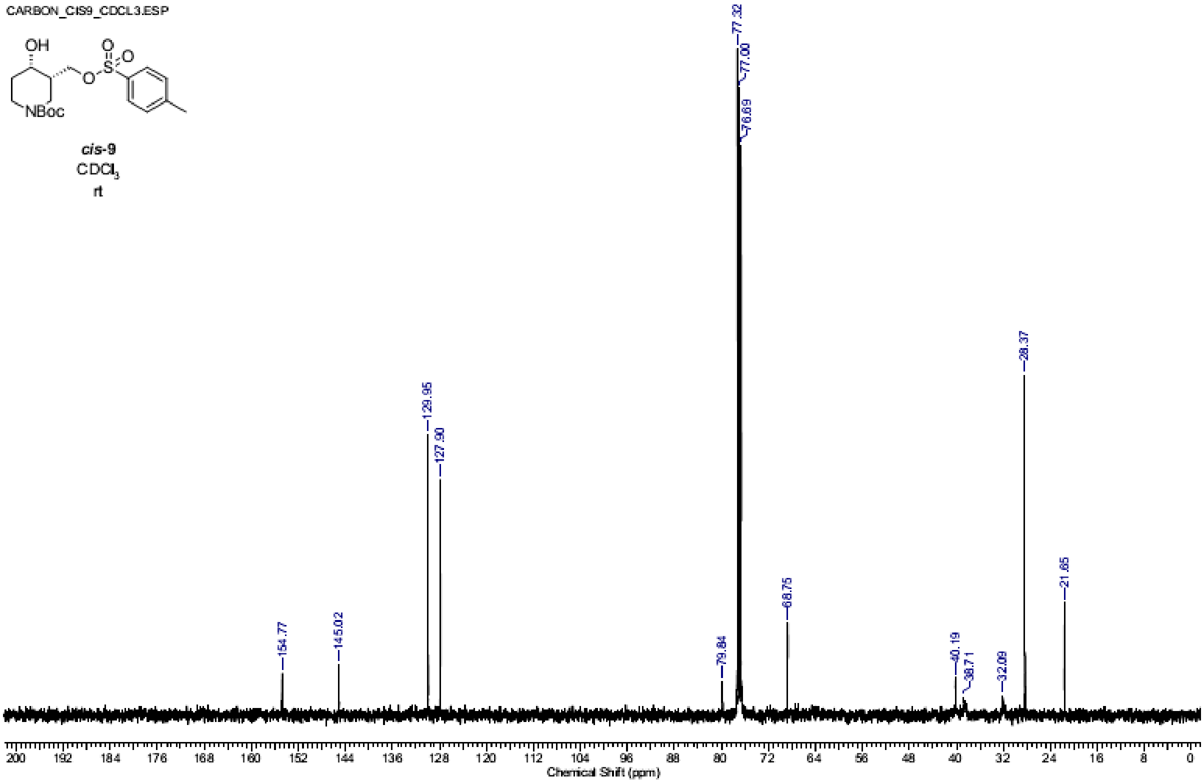

CARBON_CIS9_CDCL3.ESP
OH
O
O
S
O
NBoc
cis-9
CDCl3
rt
154.77
145.02
129.95
127.90
79.84
77.32
77.00
76.69
68.75
40.19
38.71
32.09
28.37
21.65
200
192
184
176
168
160
152
144
136
128
120
112
104
96
88
80
72
64
56
48
40
32
24
16
8
0
Chemical Shift (ppm)

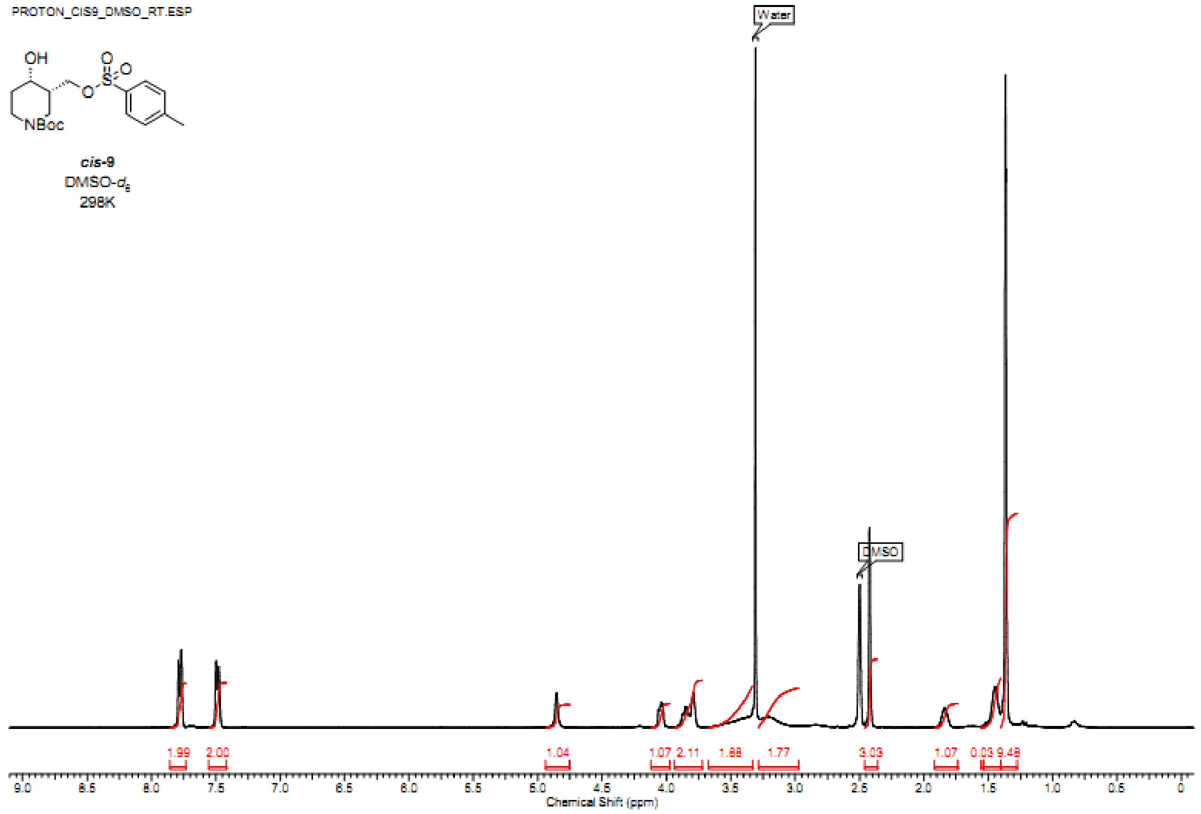
PROTON_CIS9_DMSO_RT.ESP
OH
O
S
O
O
NBoc
cis-9
DMSO-d6
298K
Water
DMSO
1.99
2.00
1.04
1.07
2.11
1.88
1.77
3.03
1.07
0.03
9.48
9.0 8.5 8.0 7.5 7.0 6.5 6.0 5.5 5.0 4.5 4.0 3.5 3.0 2.5 2.0 1.5 1.0 0.5 0
Chemical Shift (ppm)

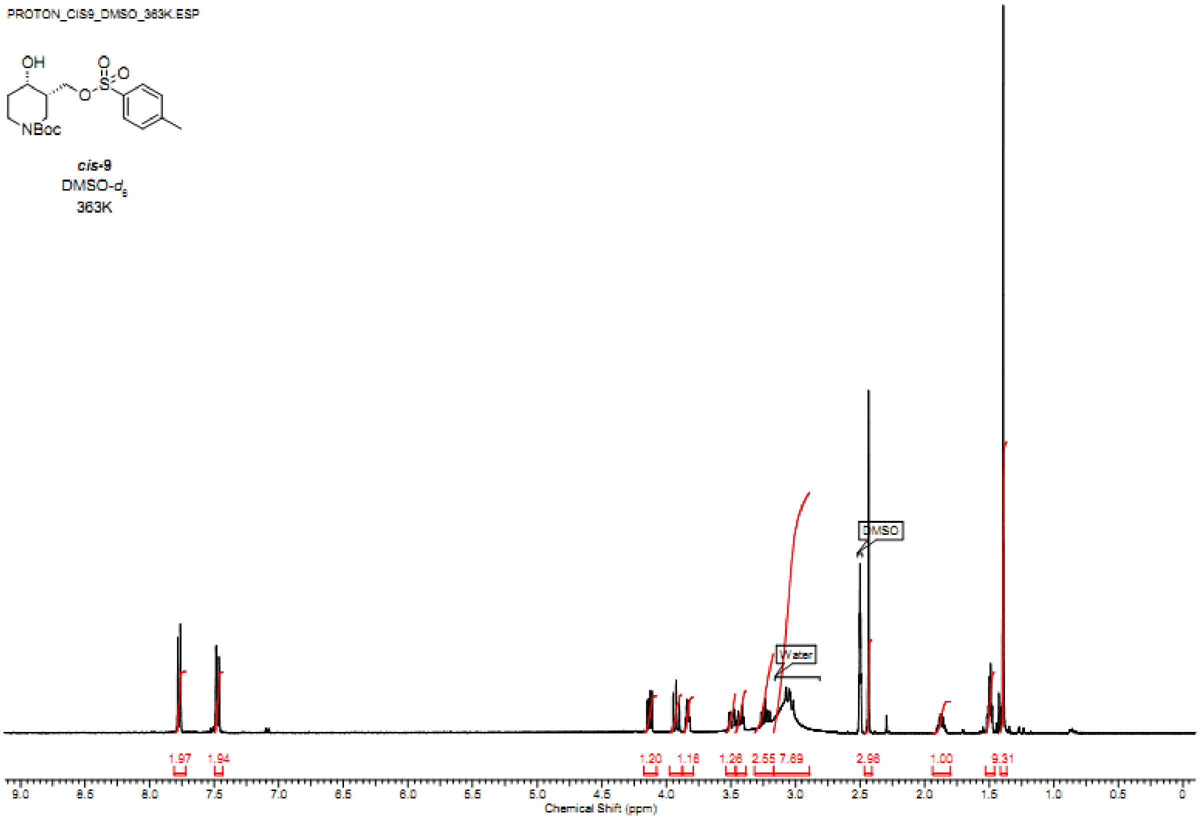
PROTON_CIS9_DMSO_363K.ESP
OH
O
S
O
O
NBoc
cis-9
DMSO-d6
363K
Water
DMSO
1.97
1.94
1.20
1.18
1.26
2.55
7.69
2.98
1.00
9.31
9.0 8.5 8.0 7.5 7.0 6.5 6.0 5.5 5.0 4.5 4.0 3.5 3.0 2.5 2.0 1.5 1.0 0.5 0
Chemical Shift (ppm)

CARBON_CIS9_RT.ESP

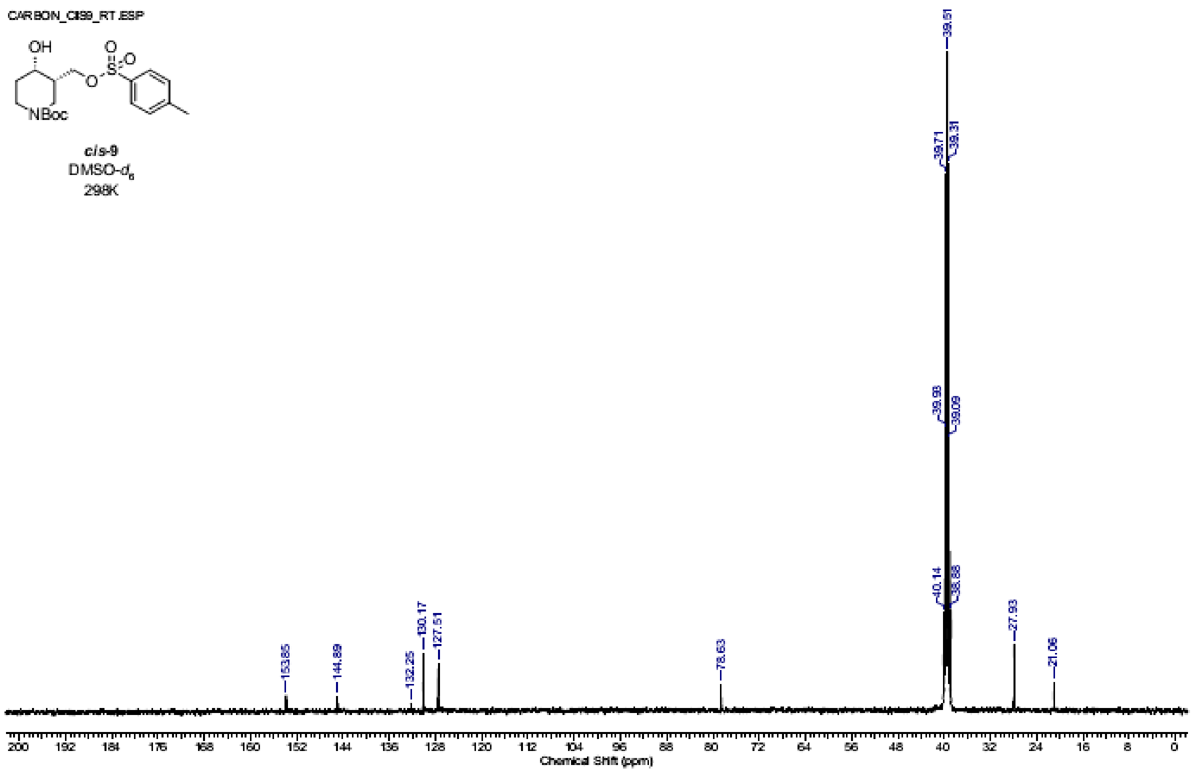

CARBON_CIS9_343K.ESP

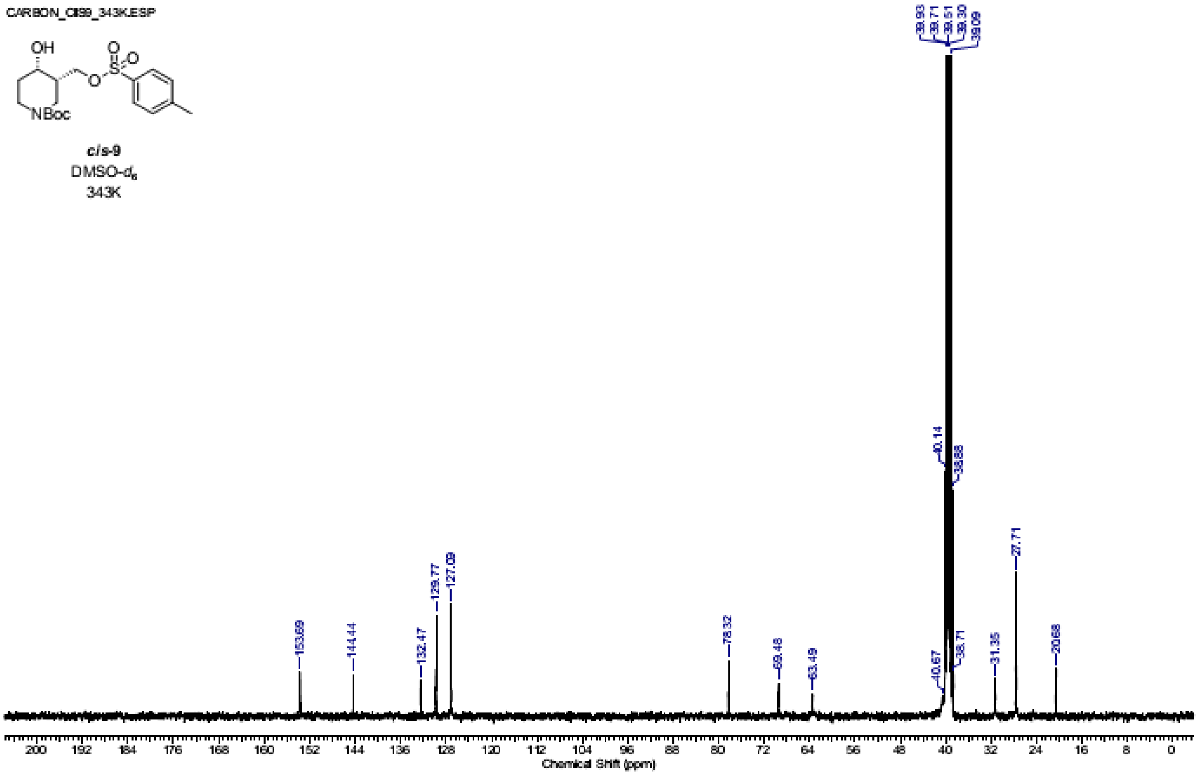

6. *N*-Boc-3-((3,4-(methylenedioxy)phenoxy)methyl)piperidin-4-ol (10)

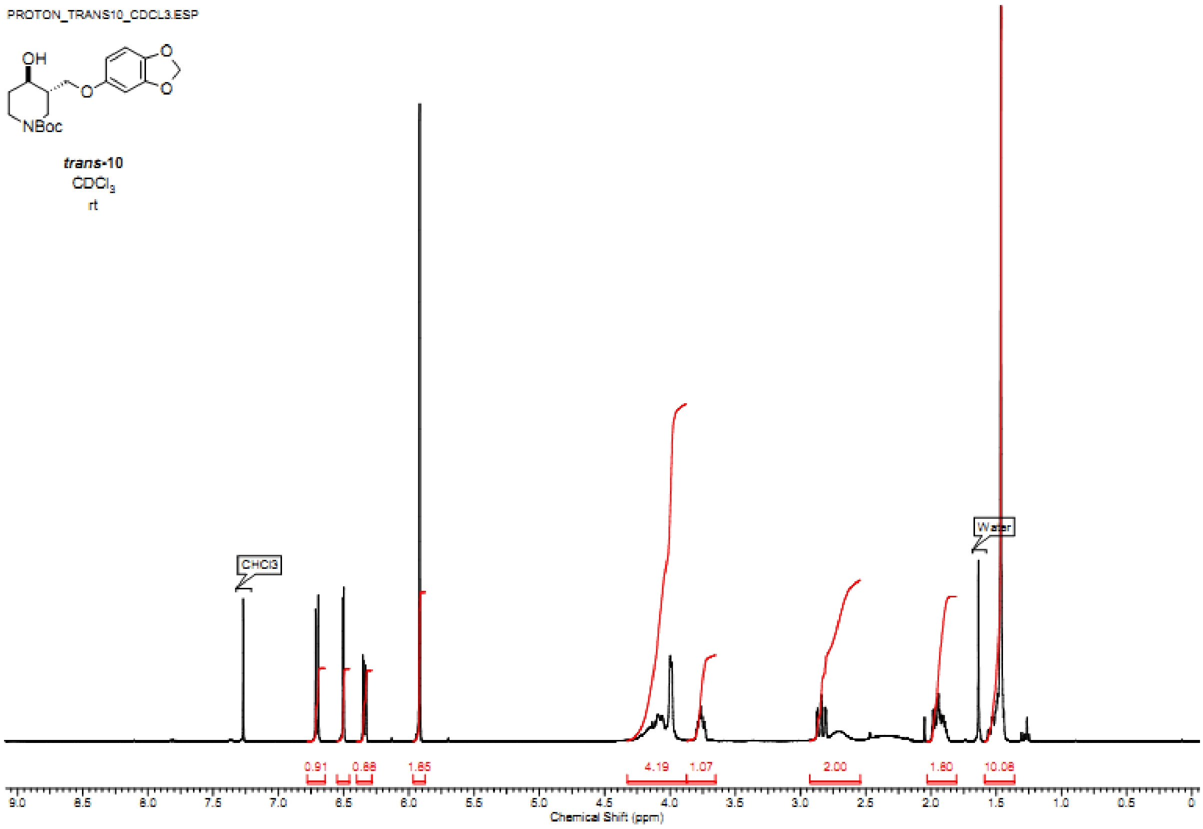

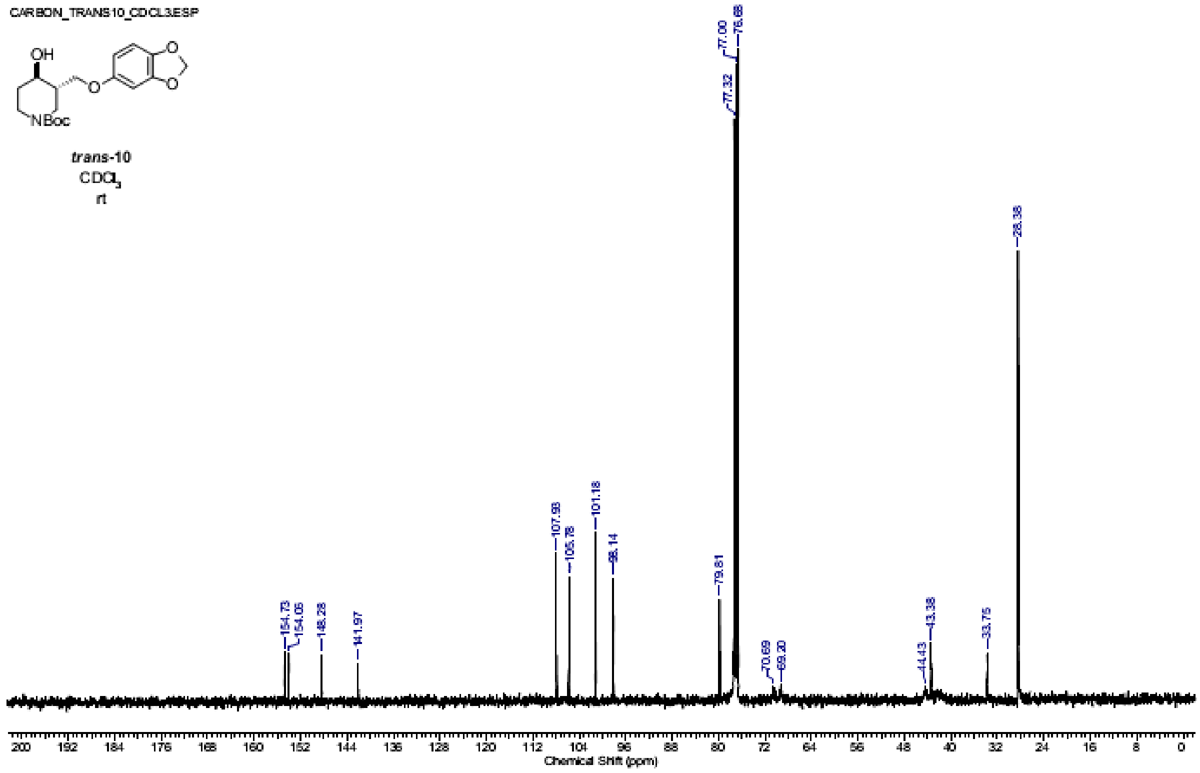

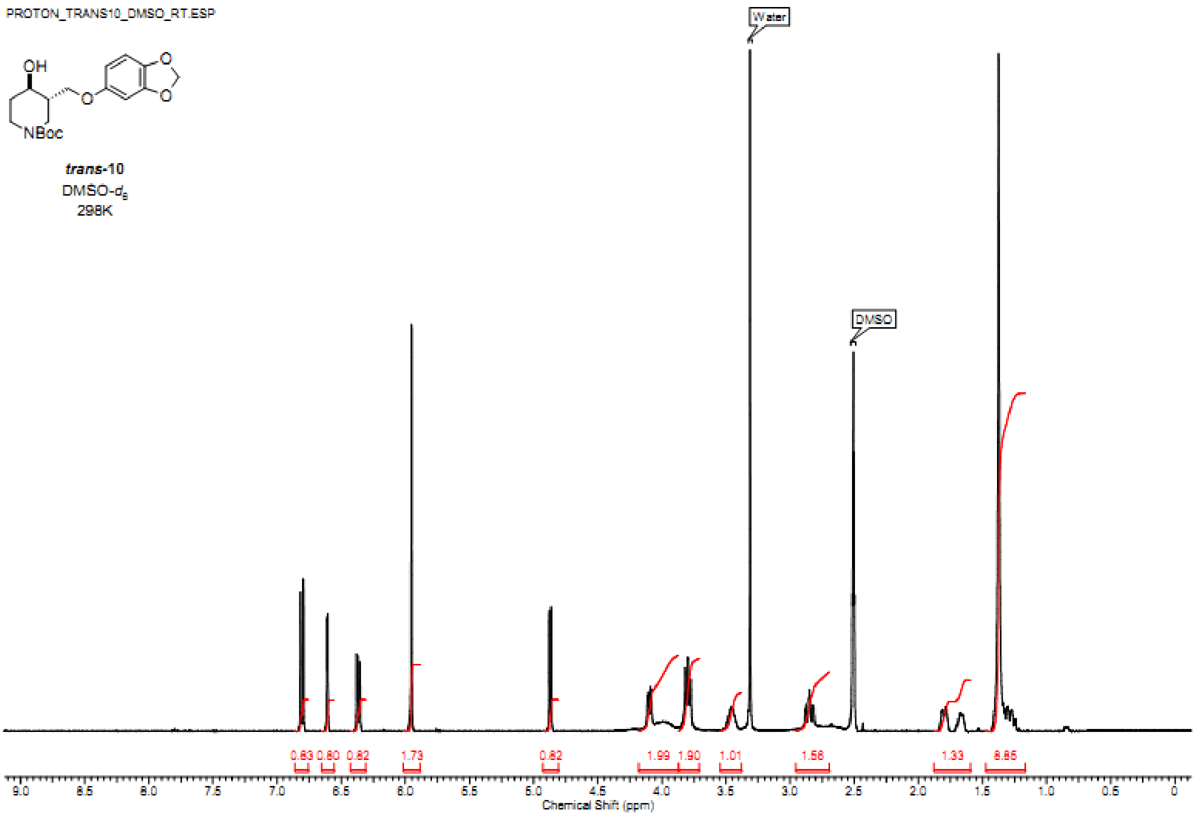
PROTON_TRANS10_DMSO_RT.ESP
OH
O
O
O
NBoc
trans-10
DMSO-d_6
298K
Water
DMSO
0.83 0.80 0.82 1.73 0.82 1.99 1.90 1.01 1.56 1.33 8.85
9.0 8.5 8.0 7.5 7.0 6.5 6.0 5.5 5.0 4.5 4.0 3.5 3.0 2.5 2.0 1.5 1.0 0.5 0
Chemical Shift (ppm)

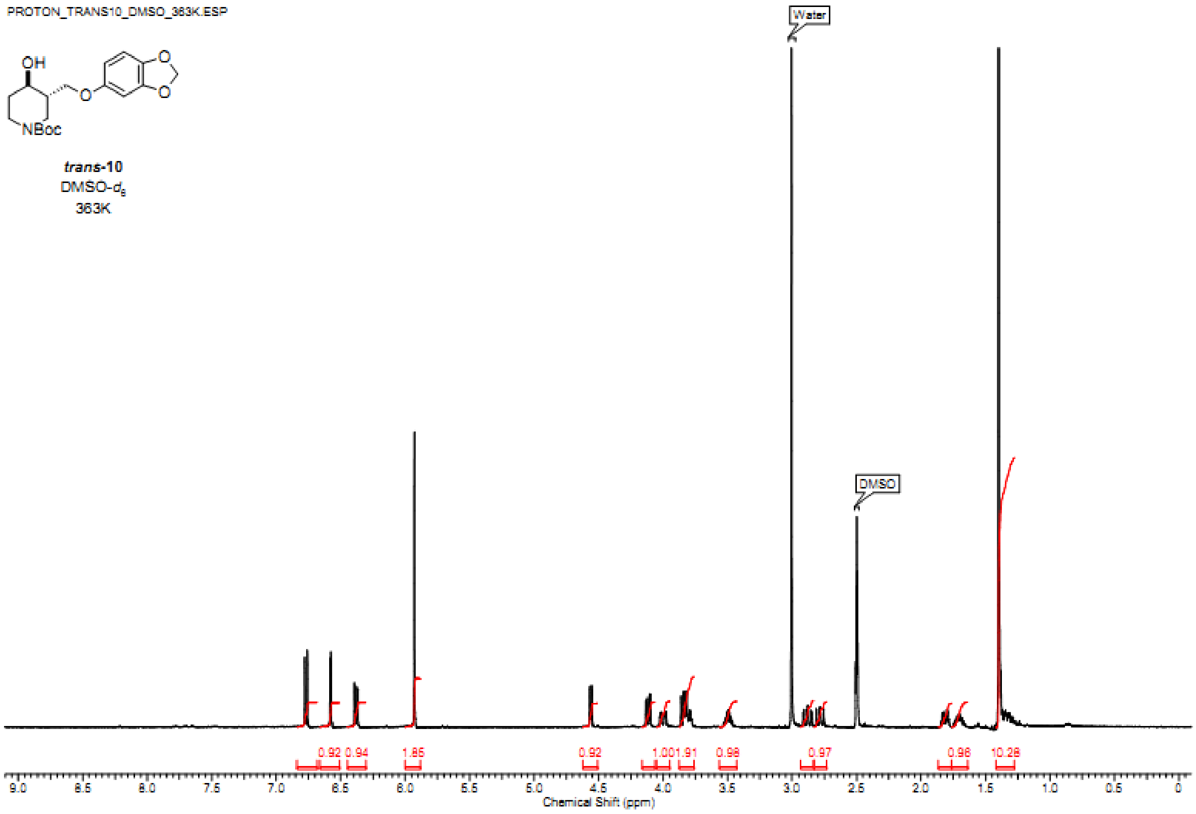
PROTON_TRANS10_DMSO_363K.ESP
OH
O
O
O
NBoc
trans-10
DMSO-d_6
363K
Water
DMSO
0.92 0.94 1.85 0.92 1.00 1.91 0.98 0.97 0.96 10.28
9.0 8.5 8.0 7.5 7.0 6.5 6.0 5.5 5.0 4.5 4.0 3.5 3.0 2.5 2.0 1.5 1.0 0.5 0
Chemical Shift (ppm)

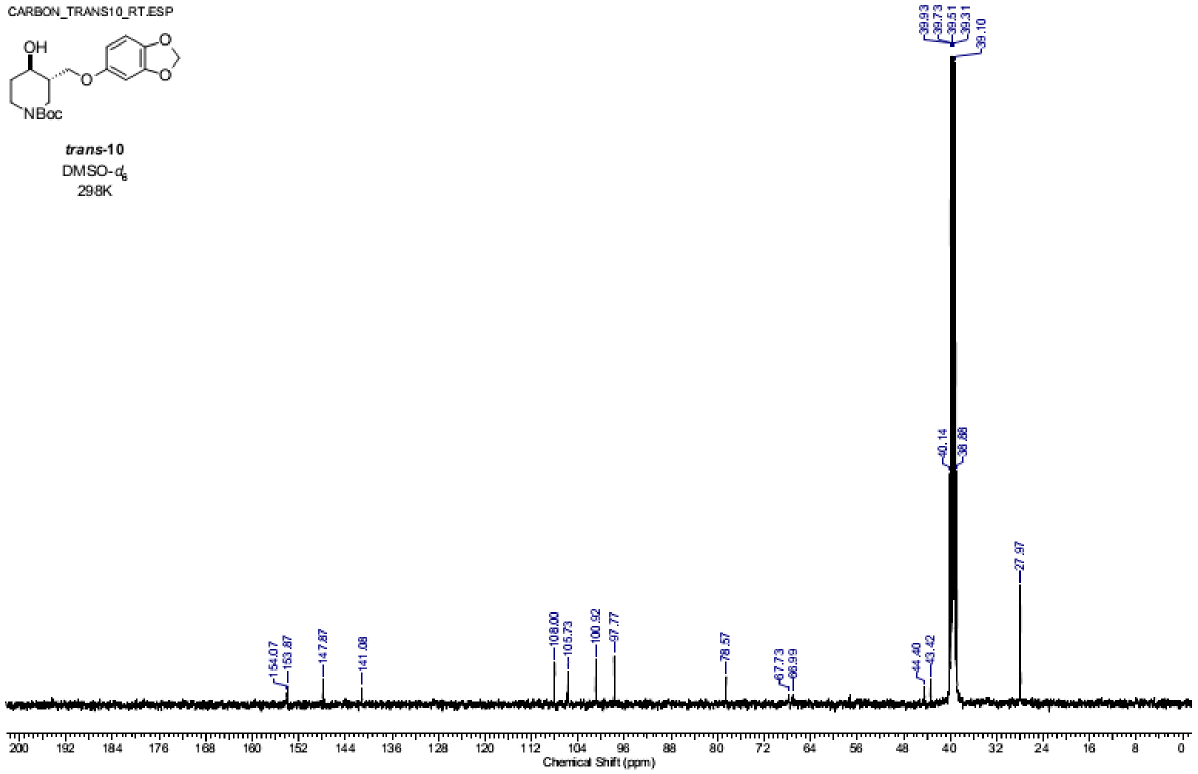
CARBON_TRANS10_RT.ESP
OH
O
O
O
NBoc
trans-10
DMSO-d_6
298K
39.93
39.73
39.51
39.31
39.10
40.14
38.88
27.97
154.07
153.87
147.87
141.08
108.00
105.73
100.92
97.77
78.57
67.73
66.99
44.40
43.42
200 192 184 176 168 160 152 144 136 128 120 112 104 96 88 80 72 64 56 48 40 32 24 16 8 0
Chemical Shift (ppm)

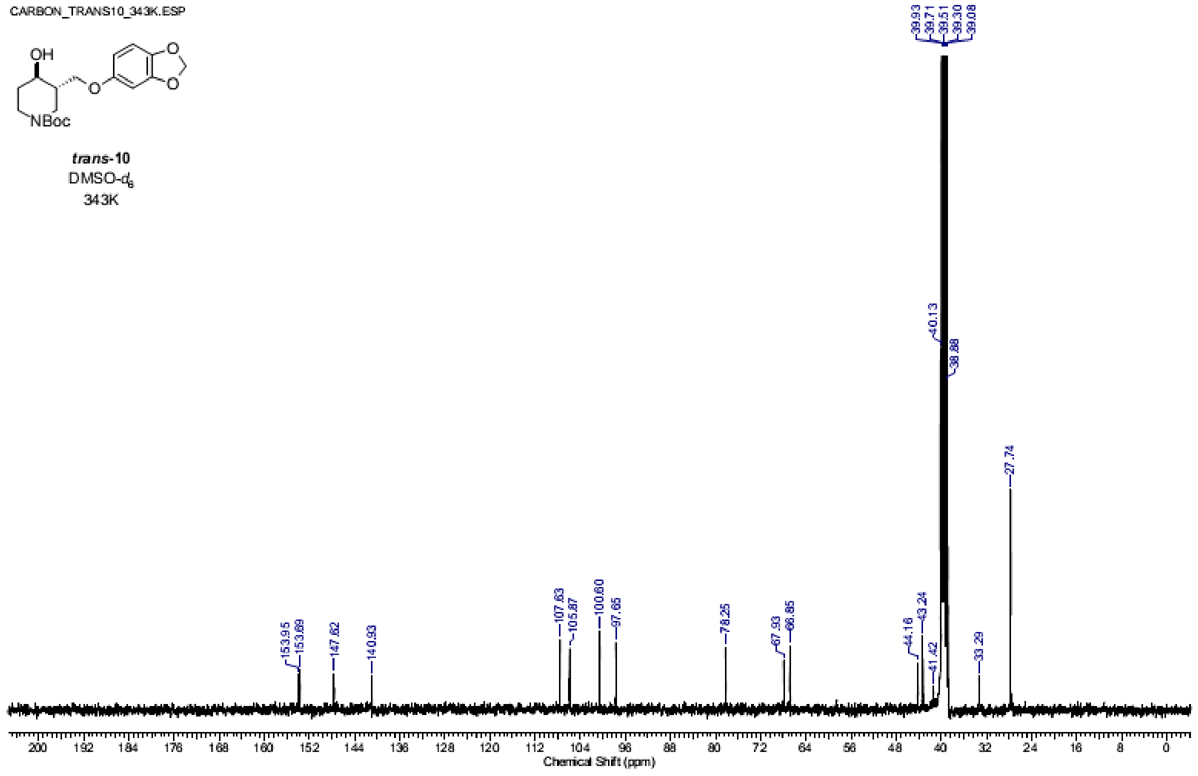
CARBON_TRANS10_343K.ESP
OH
O
O
O
NBoc
trans-10
DMSO-d_6
343K
39.93
39.71
39.51
39.30
39.08
40.13
38.88
27.74
153.95
153.69
147.62
140.93
107.63
105.87
100.80
97.65
78.25
67.93
66.85
44.16
43.24
41.42
33.29
200 192 184 176 168 160 152 144 136 128 120 112 104 96 88 80 72 64 56 48 40 32 24 16 8 0
Chemical Shift (ppm)

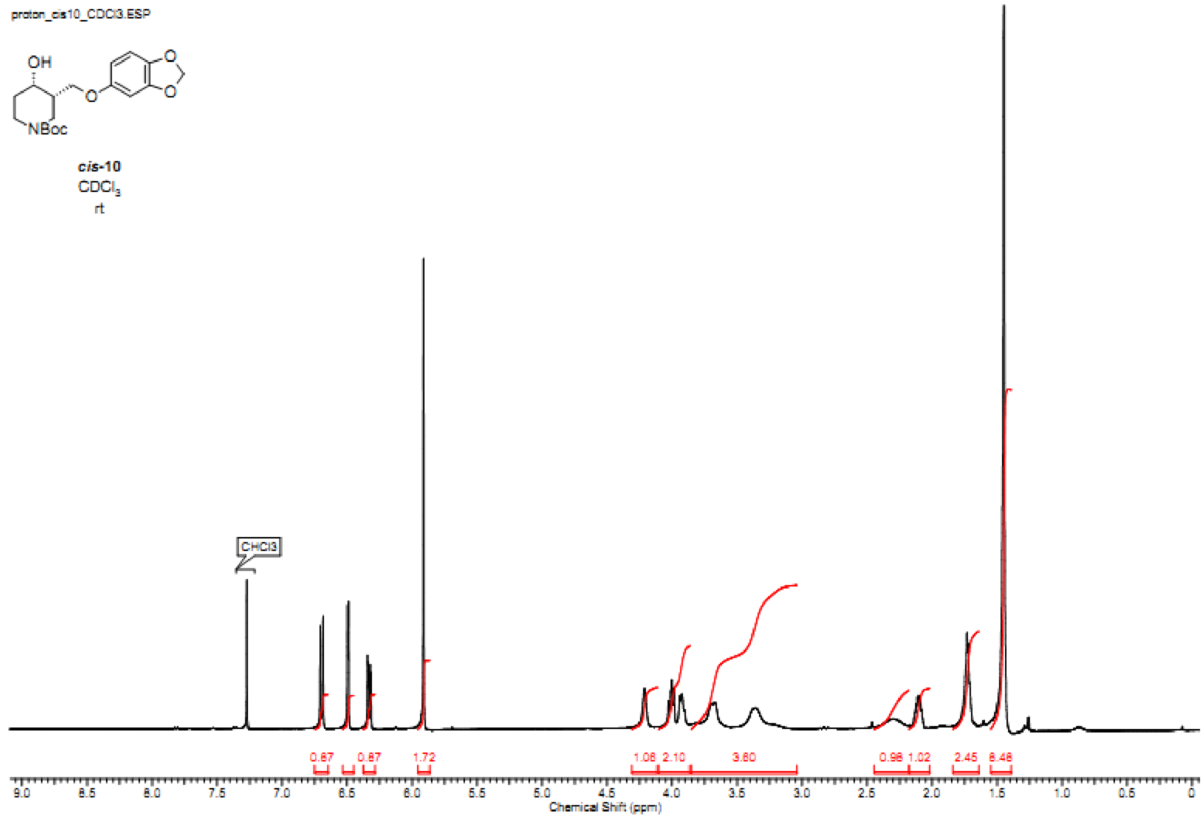

proton_cis10_CDCl3.ESP
OH
O
NBoc
cis-10
$CDCl_3$
rt
CHCl3
0.87
0.87
1.72
1.08
2.10
3.80
0.98
1.02
2.45
8.48
Chemical Shift (ppm)

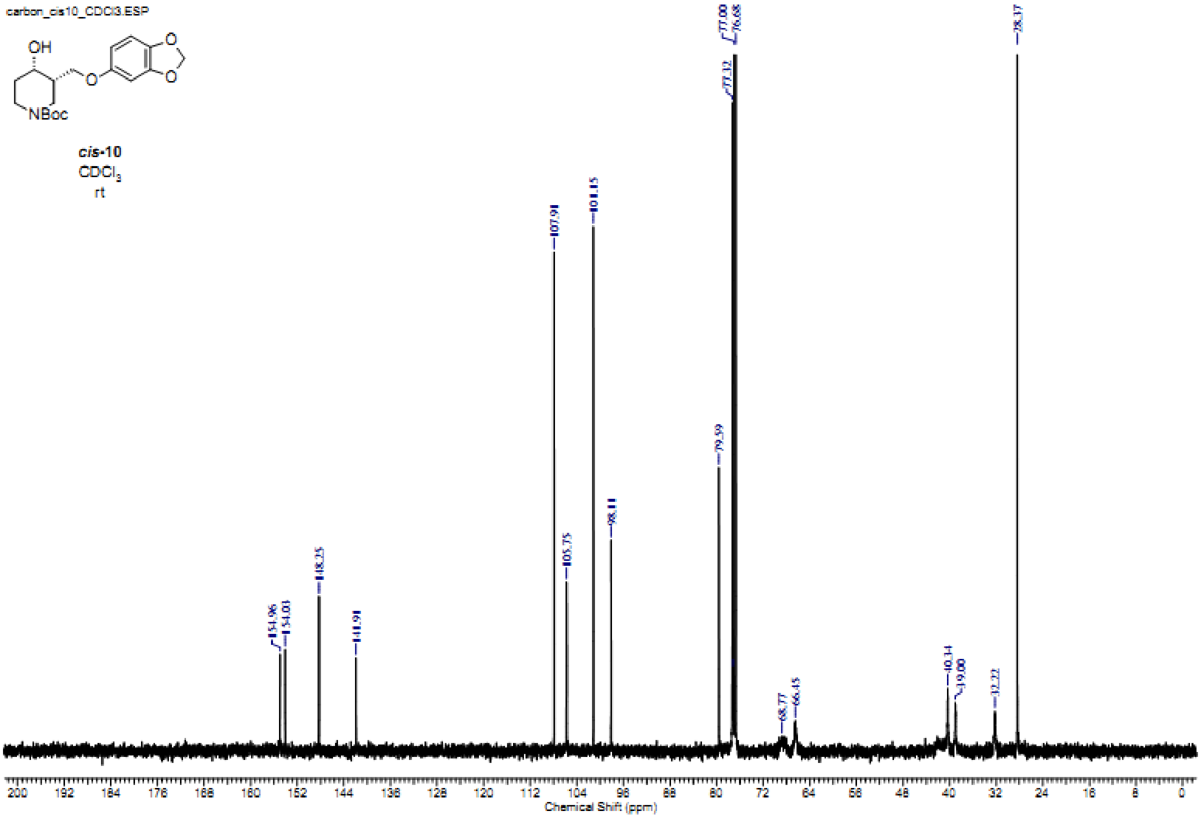

carbon_cis10_CDCl3.ESP
OH
O
NBoc
cis-10
$CDCl_3$
rt
154.96
154.03
148.25
141.91
107.91
105.75
101.15
98.11
79.59
77.32
77.00
76.68
68.77
66.15
40.14
39.00
32.22
28.37
Chemical Shift (ppm)

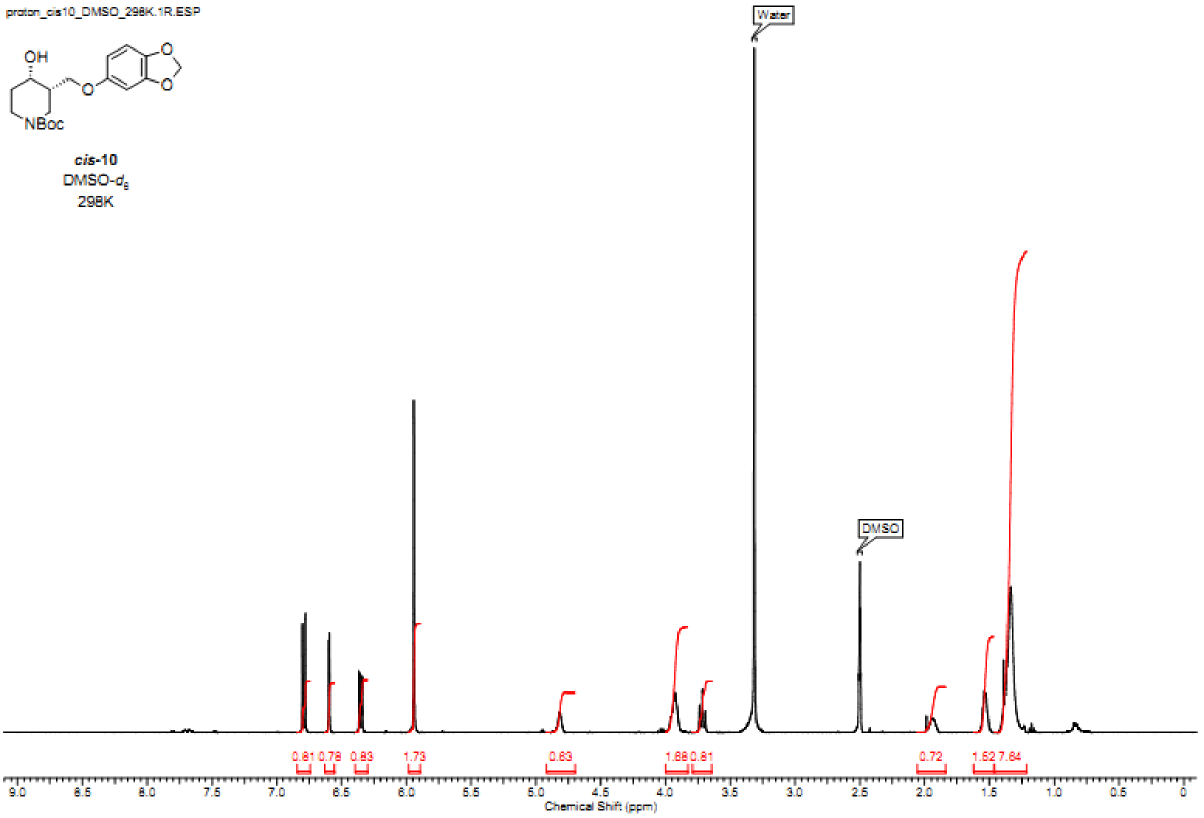
proton_cis10_DMSO_298K.1R.ESP
OH
O
O
O
NBoc
cis-10
DMSO-d6
298K
Water
DMSO
0.81 0.78 0.83 1.73 0.83 1.66 0.81 0.72 1.52 7.84
Chemical Shift (ppm)

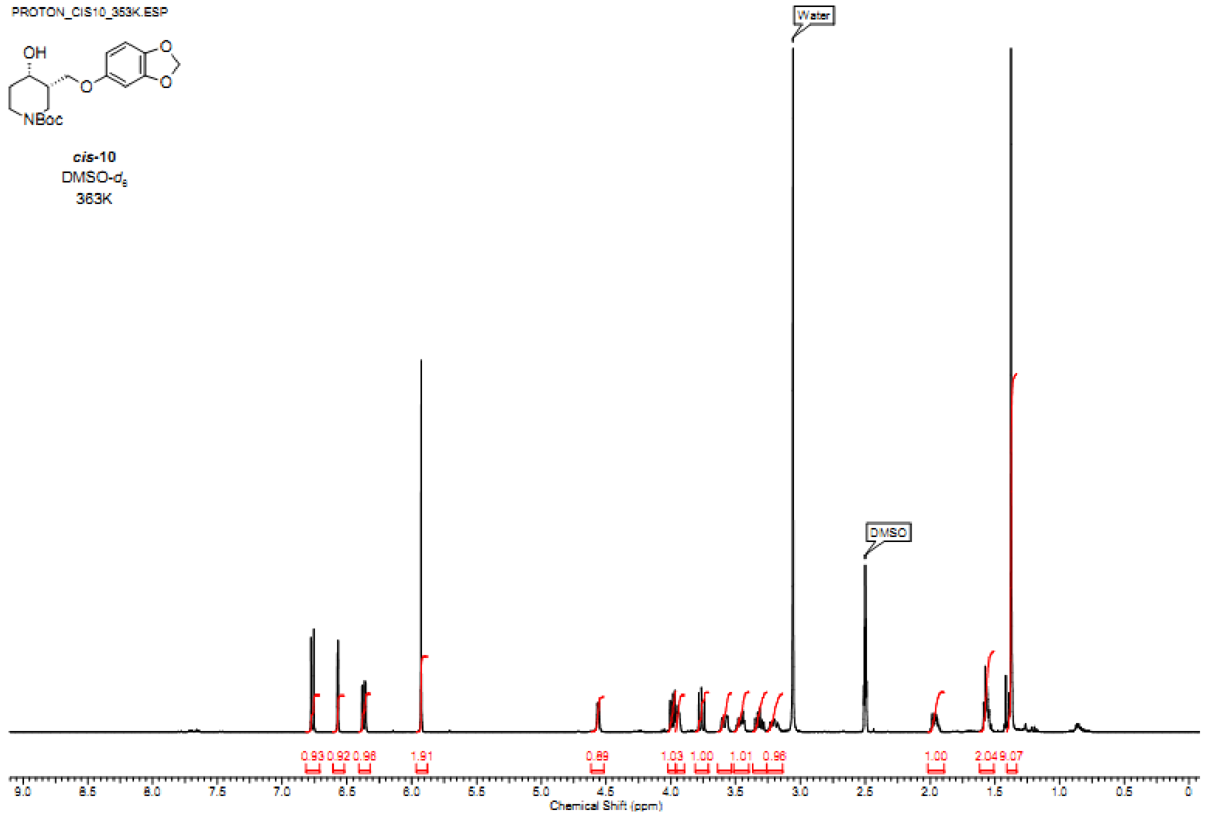
PROTON_CIS10_353K.ESP
OH
O
O
O
NBoc
cis-10
DMSO-d6
363K
Water
DMSO
0.93 0.92 0.98 1.91 0.89 1.03 1.00 1.01 0.96 1.00 2.04 9.07
Chemical Shift (ppm)

CARBON_CIS10_RT.ESP

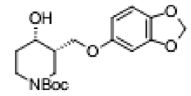

cis-**10**
DMSO-d_6
298K

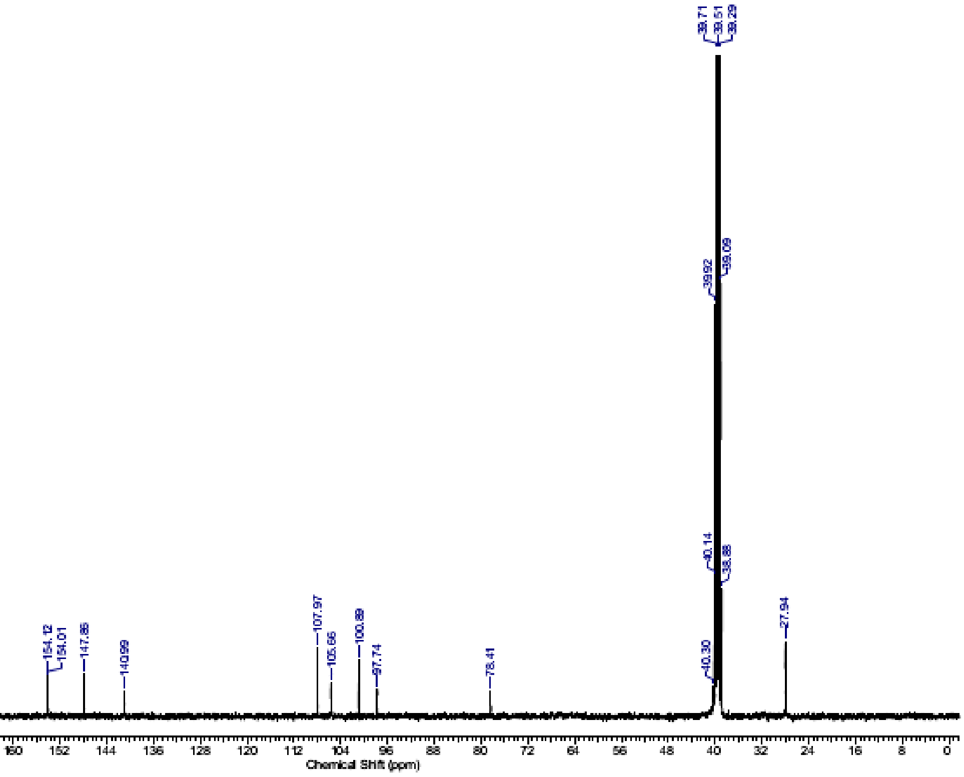

CARBON_CIS10_353K.ESP

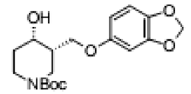

cis-**10**
DMSO-d_6
353K

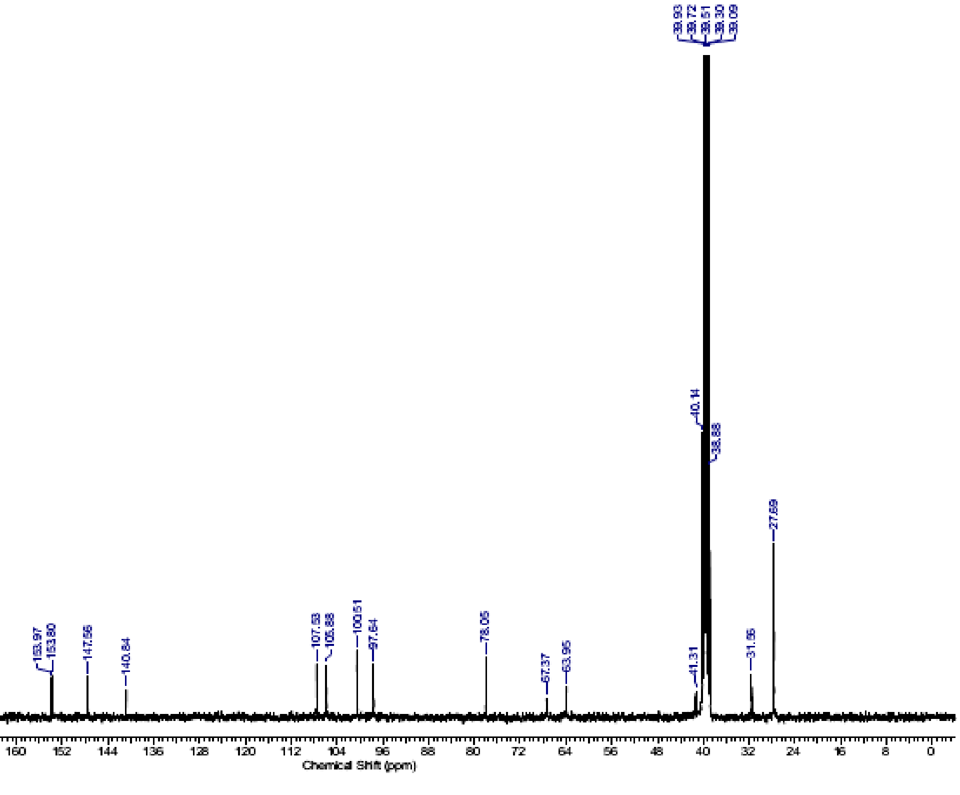

7. ***N*-Boc-4-bromo-3-((3,4-(methylenedioxy)phenoxy)methyl)piperidine (6)**

Trans diastereoisomer degrades rapidly

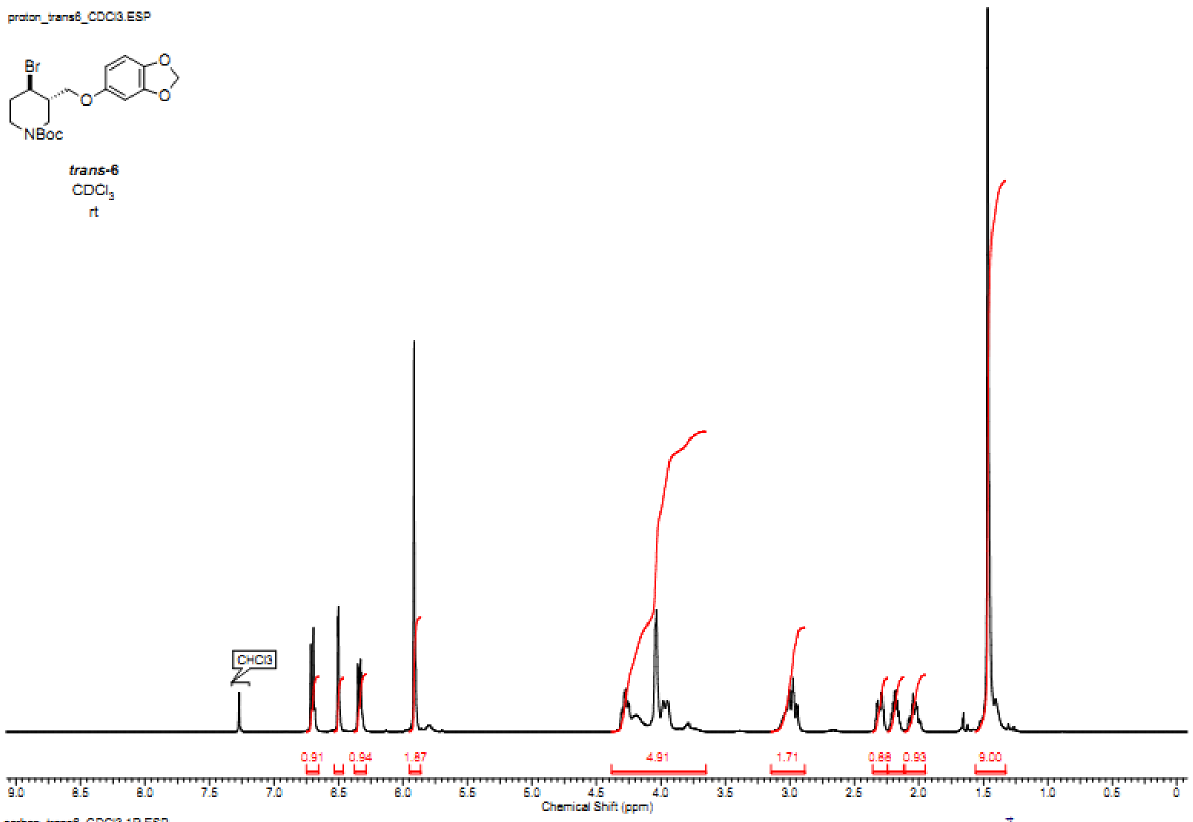

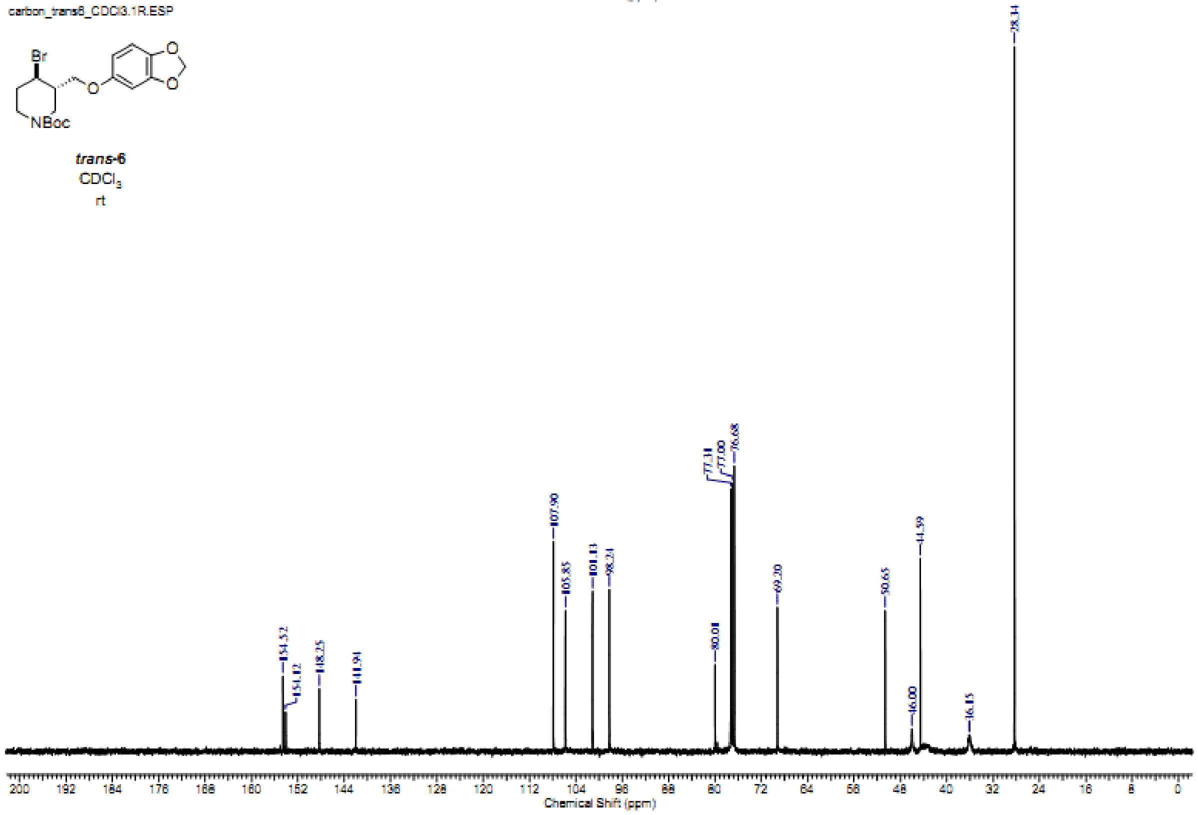

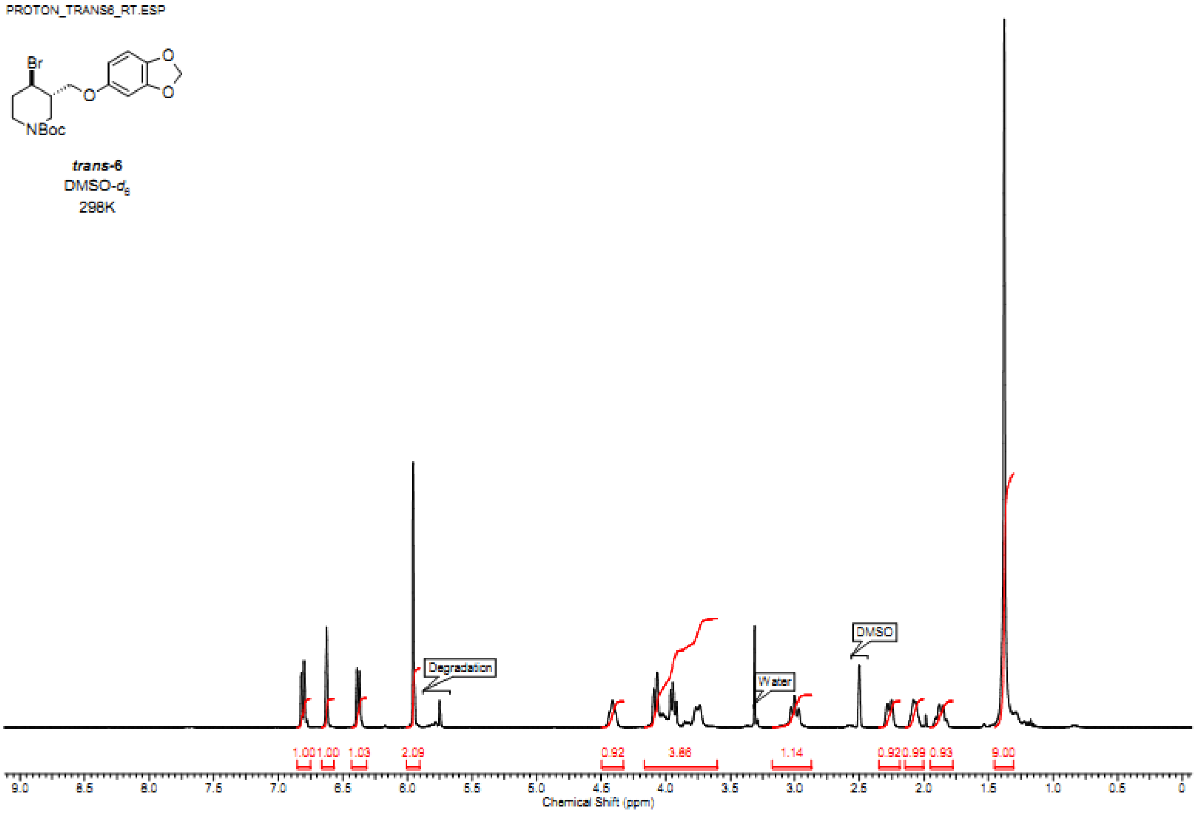

PROTON_TRANS6_RT.ESP
trans-6
DMSO-d_6
298K
Degradation
Water
DMSO
1.00 1.00 1.03 2.09 0.92 3.86 1.14 0.92 0.99 0.93 9.00
Chemical Shift (ppm)

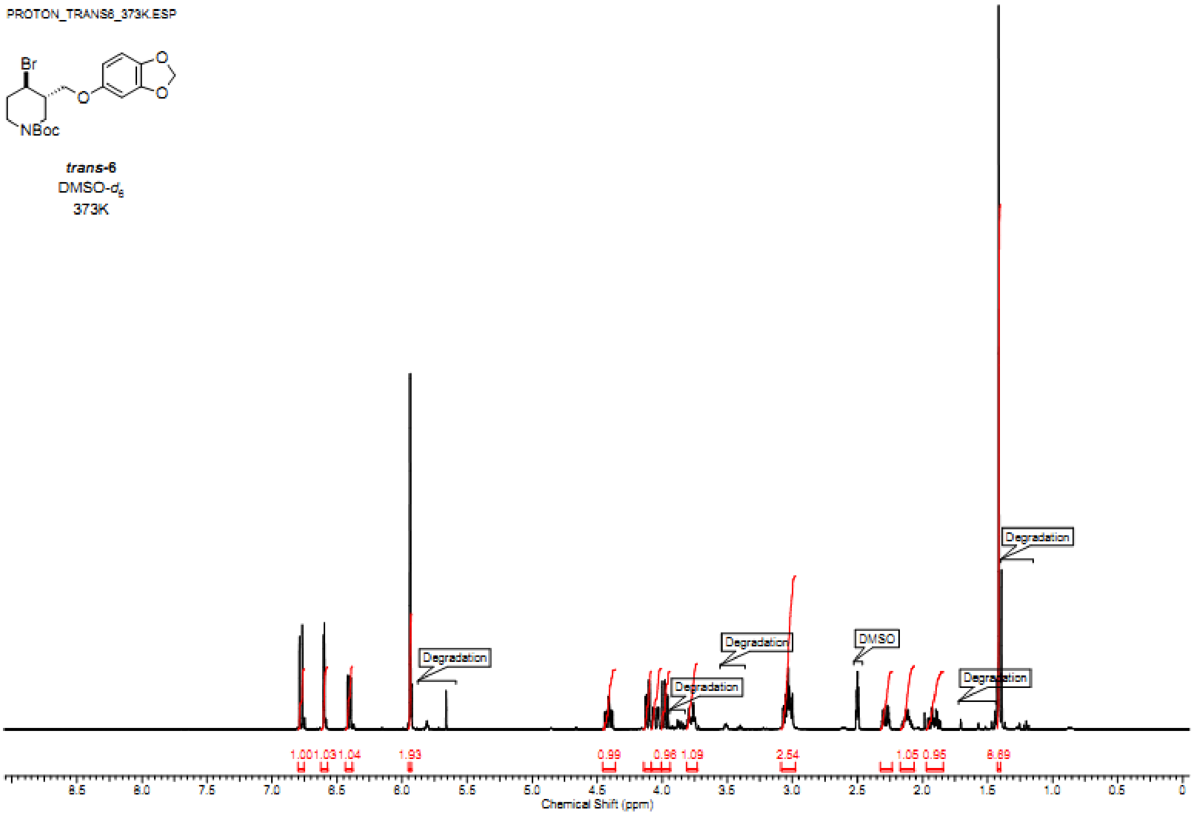

PROTON_TRANS6_373K.ESP
trans-6
DMSO-d_6
373K
Degradation
DMSO
1.00 1.03 1.04 1.93 0.99 0.98 1.09 2.54 1.05 0.95 8.89
Chemical Shift (ppm)

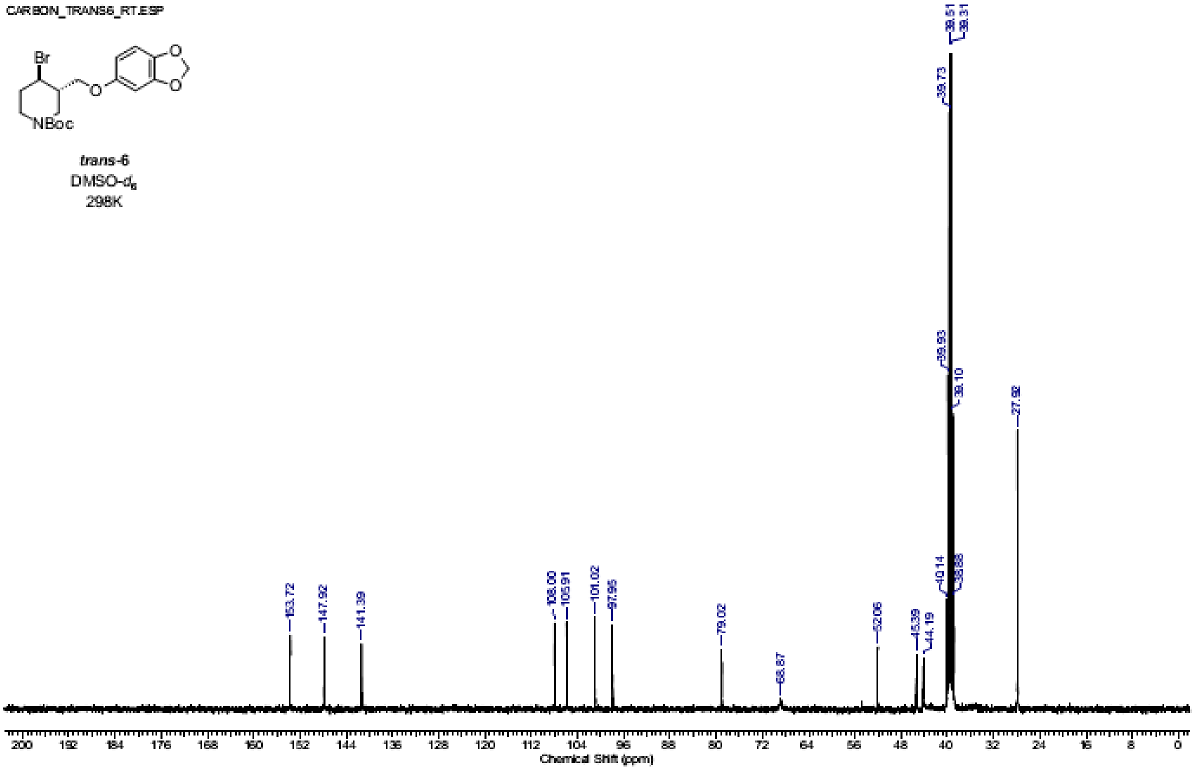
CARBON_TRANS6_RT.ESP
Br
O
O
O
NBoc
trans-6
DMSO-d6
298K
Chemical Shift (ppm)

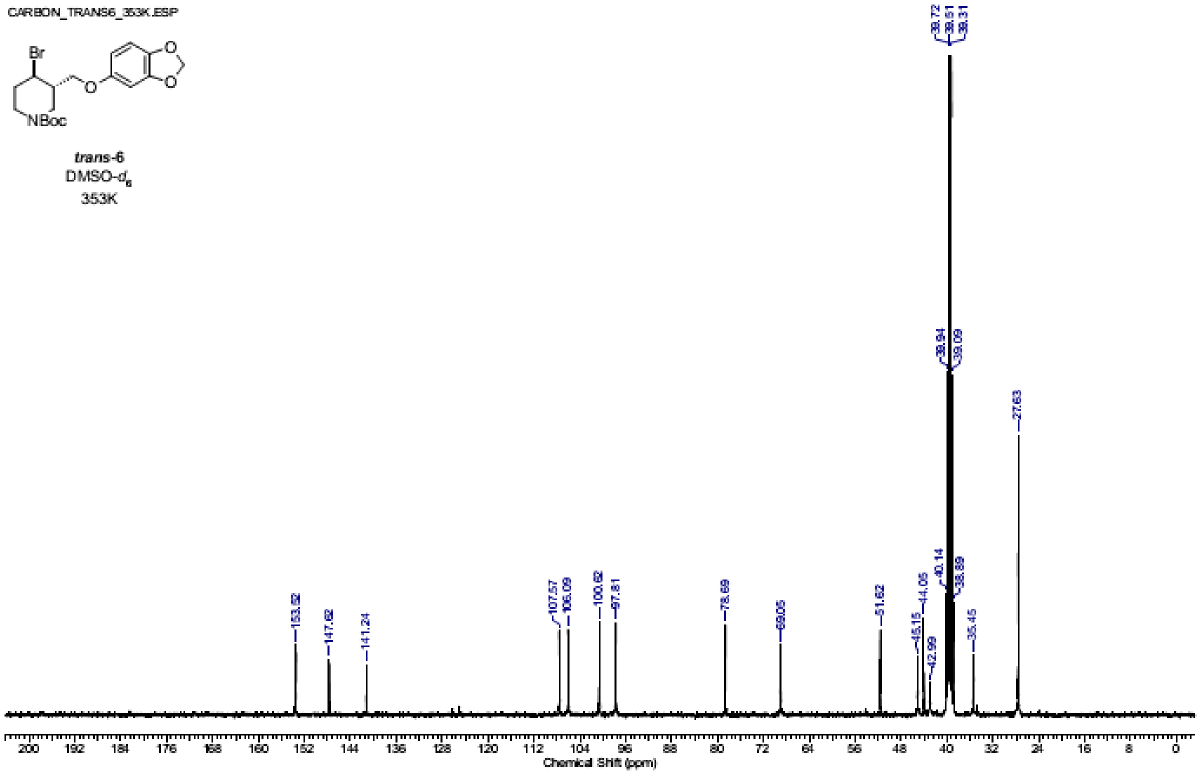
CARBON_TRANS6_353K.ESP
Br
O
O
O
NBoc
trans-6
DMSO-d6
353K
Chemical Shift (ppm)

Cis diastereoisomer degrades after a few days

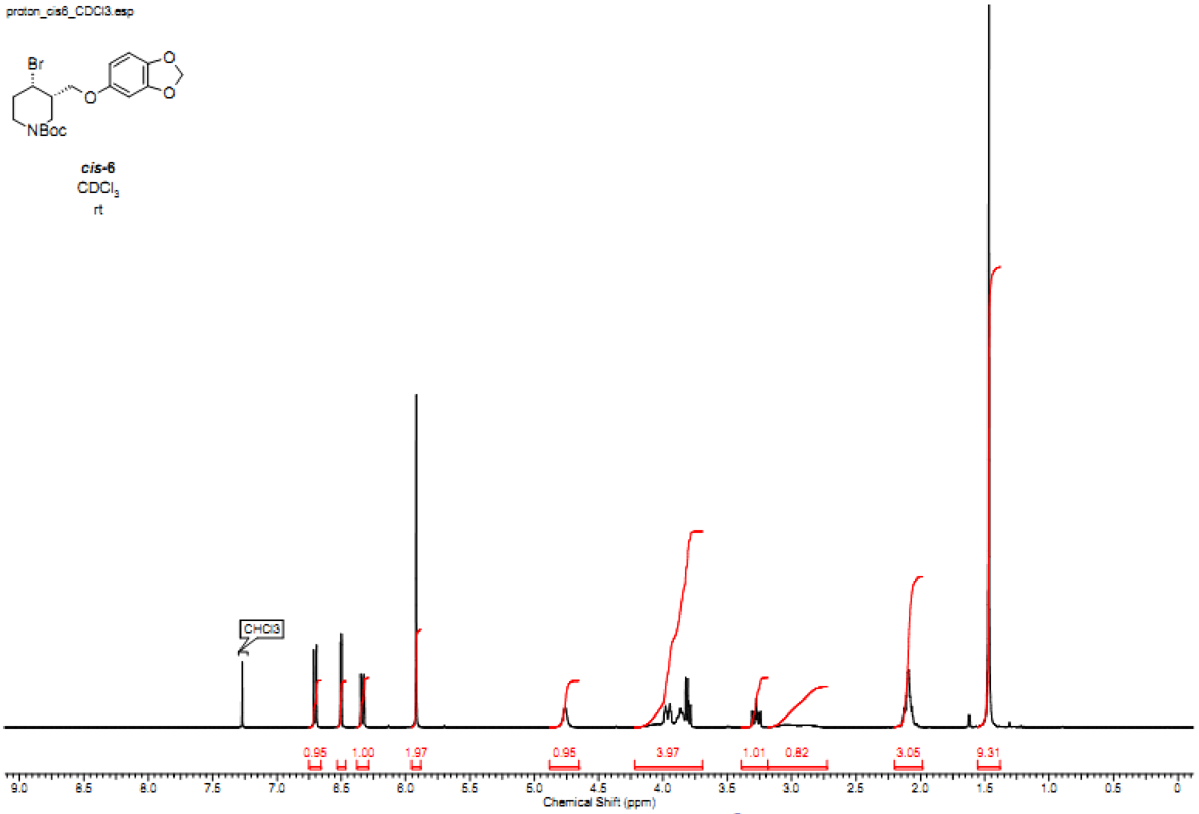

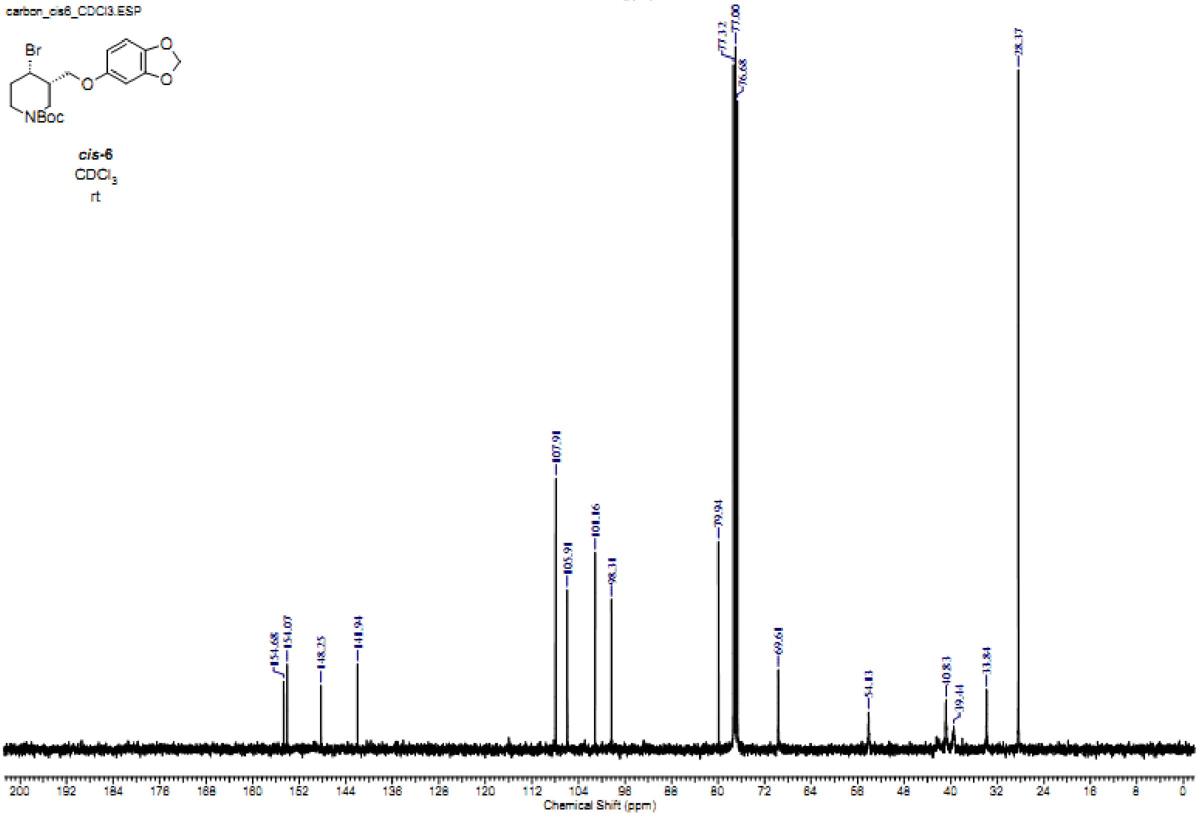

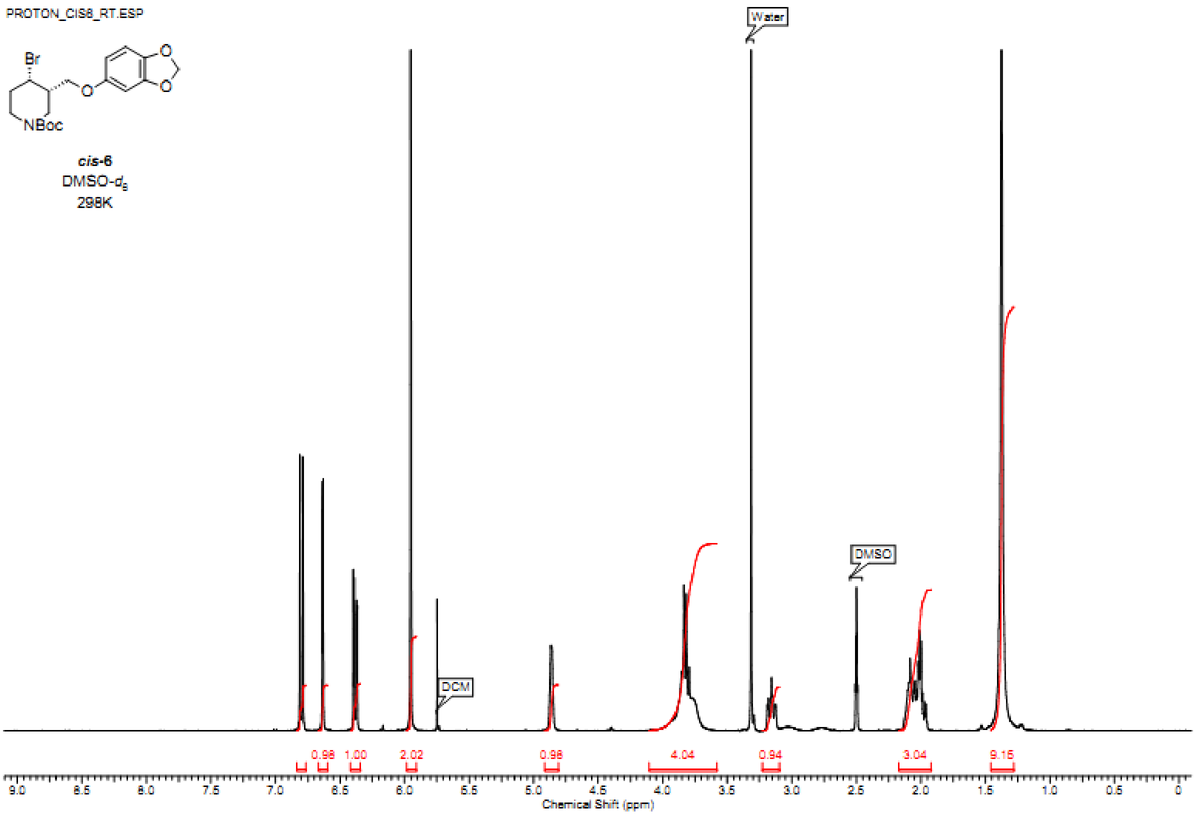
PROTON_CIS6_RT.ESP
Br
O
O
O
NBoc
cis-6
DMSO-d_6
298K
Water
DMSO
DCM
0.98
1.00
2.02
0.98
4.04
0.94
3.04
9.15
9.0 8.5 8.0 7.5 7.0 6.5 6.0 5.5 5.0 4.5 4.0 3.5 3.0 2.5 2.0 1.5 1.0 0.5 0
Chemical Shift (ppm)

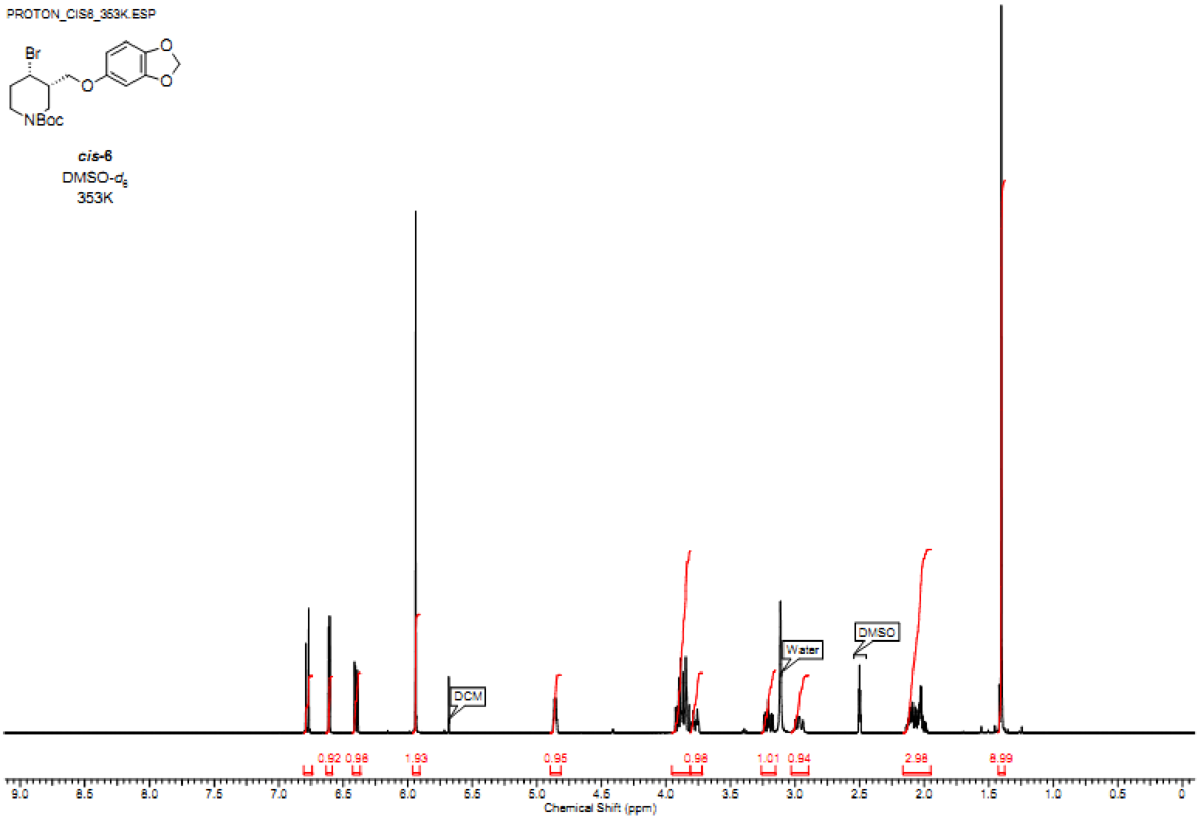
PROTON_CIS6_353K.ESP
Br
O
O
O
NBoc
cis-6
DMSO-d_6
353K
DCM
Water
DMSO
0.92
0.98
1.93
0.95
0.98
1.01
0.94
2.98
8.99
9.0 8.5 8.0 7.5 7.0 6.5 6.0 5.5 5.0 4.5 4.0 3.5 3.0 2.5 2.0 1.5 1.0 0.5 0
Chemical Shift (ppm)

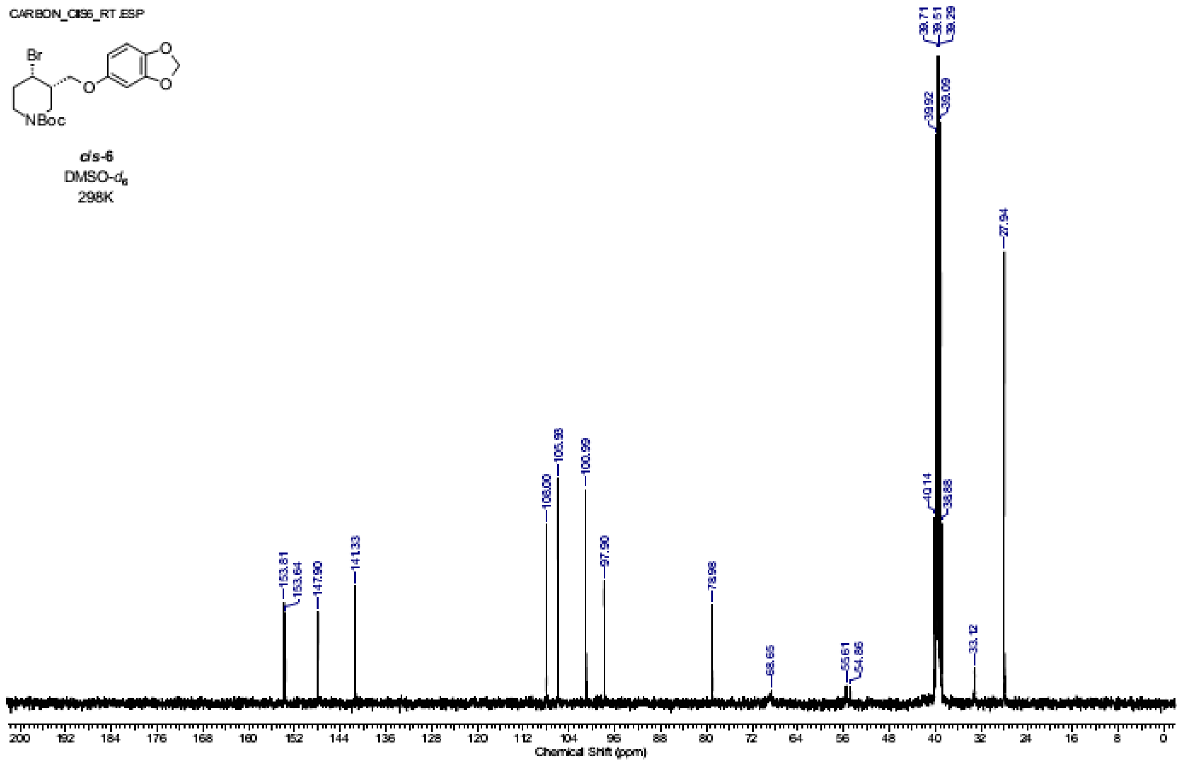

8. ***N*-Boc-4-(*p*-fluorophenyl)-3-[3,4-(methylenedioxy)phenoxymethyl]piperidine (11)**

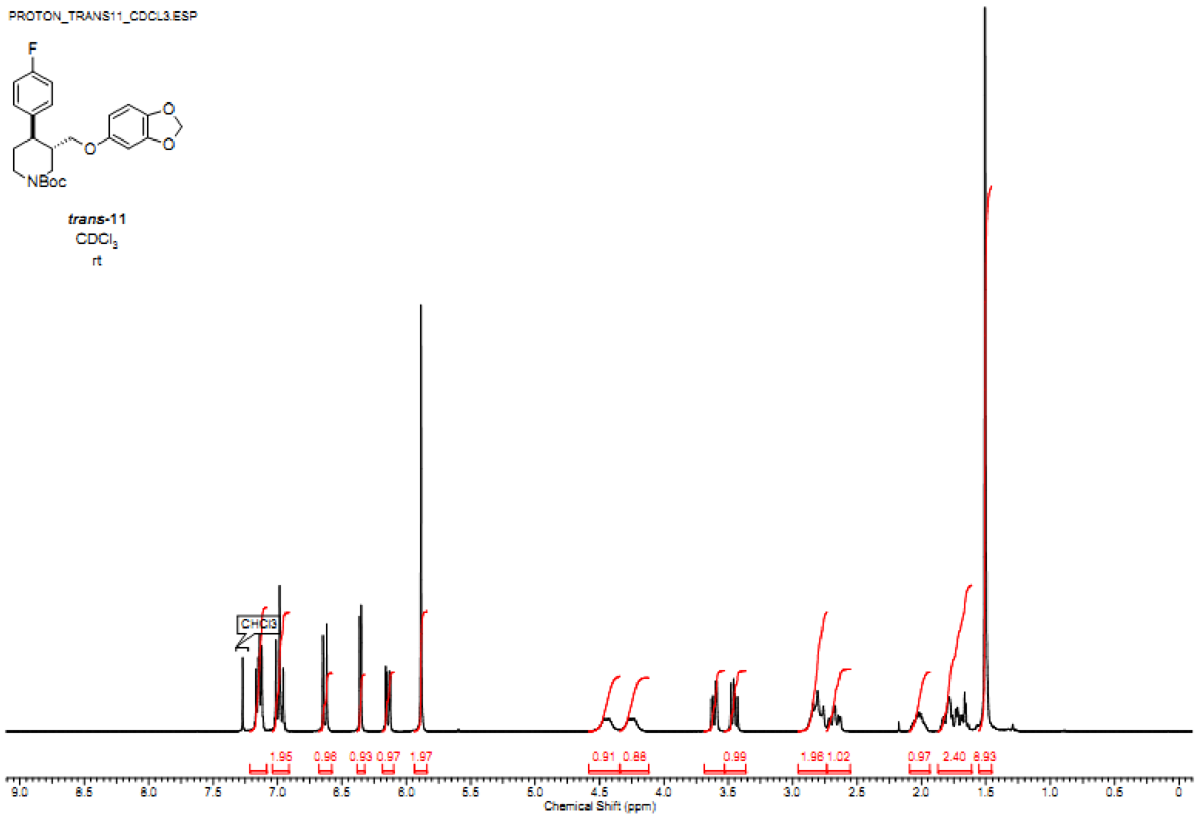

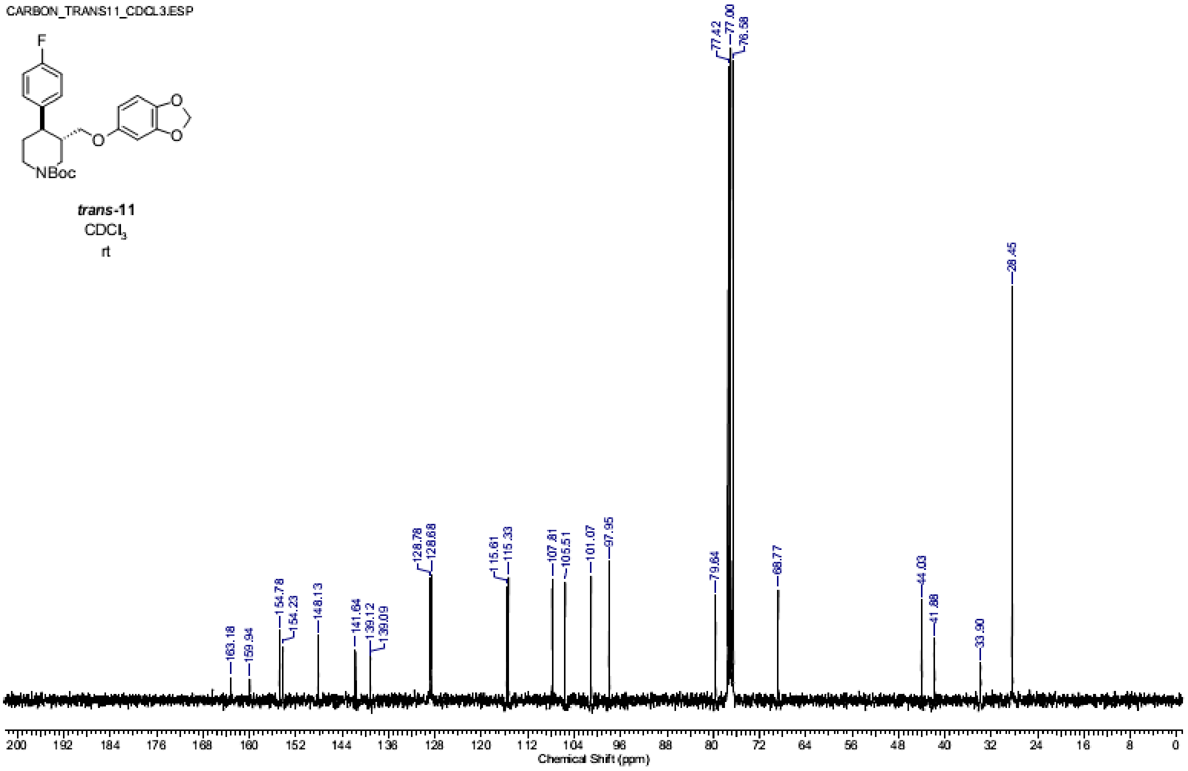
CARBON_TRANS11_CDCL3.ESP
F
O
O
O
NBoc
trans-11
$CDCl_3$
rt
77.42
77.00
76.58
28.45
163.18
159.94
154.78
154.23
148.13
141.64
139.12
139.09
128.78
128.68
115.61
115.33
107.81
105.51
101.07
97.95
79.64
68.77
44.03
41.88
33.90
200 192 184 176 168 160 152 144 136 128 120 112 104 96 88 80 72 64 56 48 40 32 24 16 8 0
Chemical Shift (ppm)

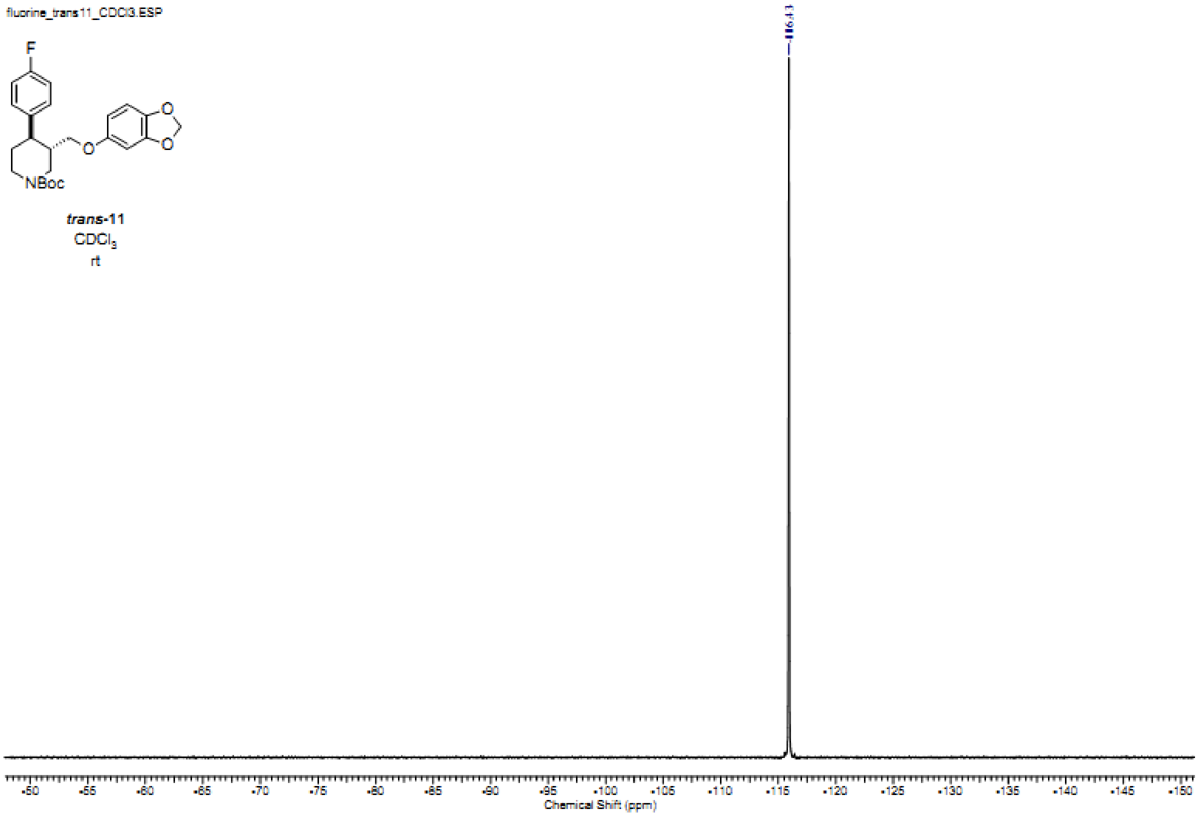
fluorine_trans11_CDCl3.ESP
F
O
O
O
NBoc
trans-11
$CDCl_3$
rt
50 55 60 65 70 75 80 85 90 95 100 105 110 115 120 125 130 135 140 145 150
Chemical Shift (ppm)

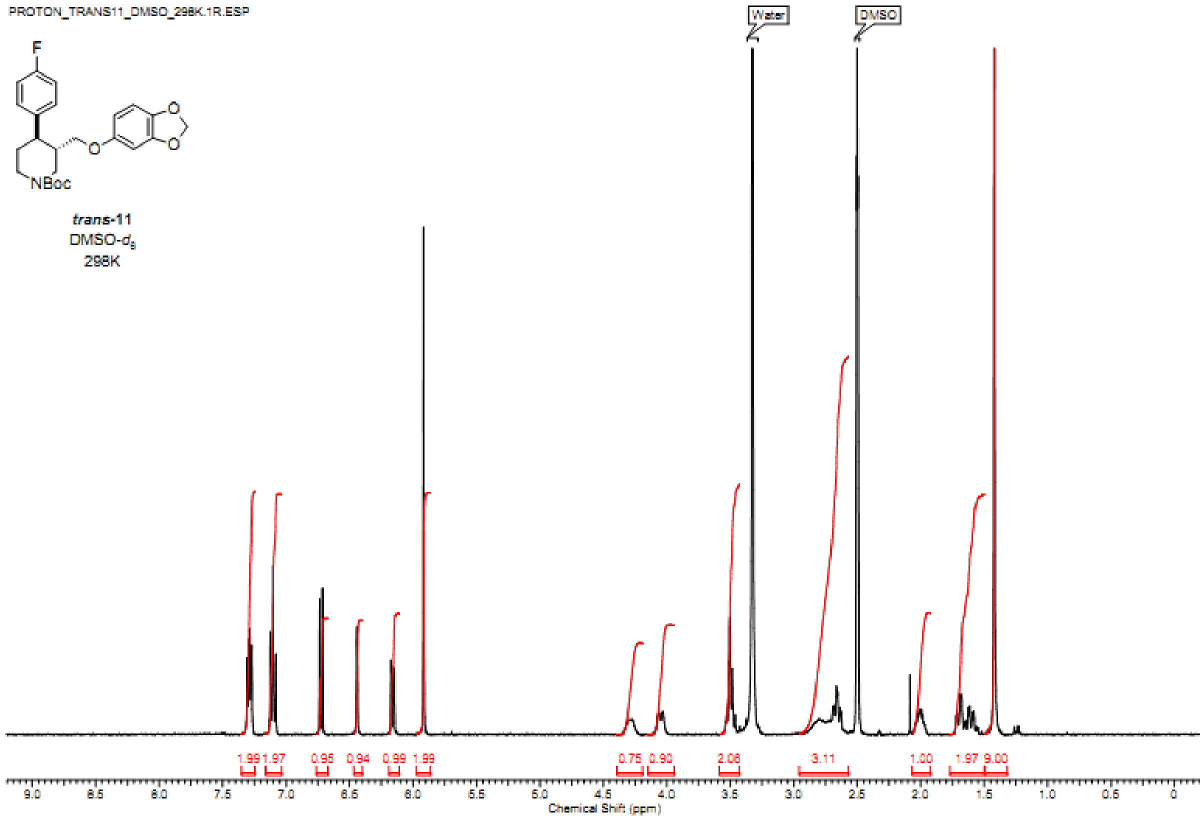
PROTON_TRANS11_DMSO_298K.1R.ESP
F
O
O
O
NBoc
trans-11
DMSO-d_6
298K
Water
DMSO
1.99 1.97 0.95 0.94 0.99 1.99 0.75 0.90 2.08 3.11 1.00 1.97 9.00
9.0 8.5 8.0 7.5 7.0 6.5 6.0 5.5 5.0 4.5 4.0 3.5 3.0 2.5 2.0 1.5 1.0 0.5 0
Chemical Shift (ppm)

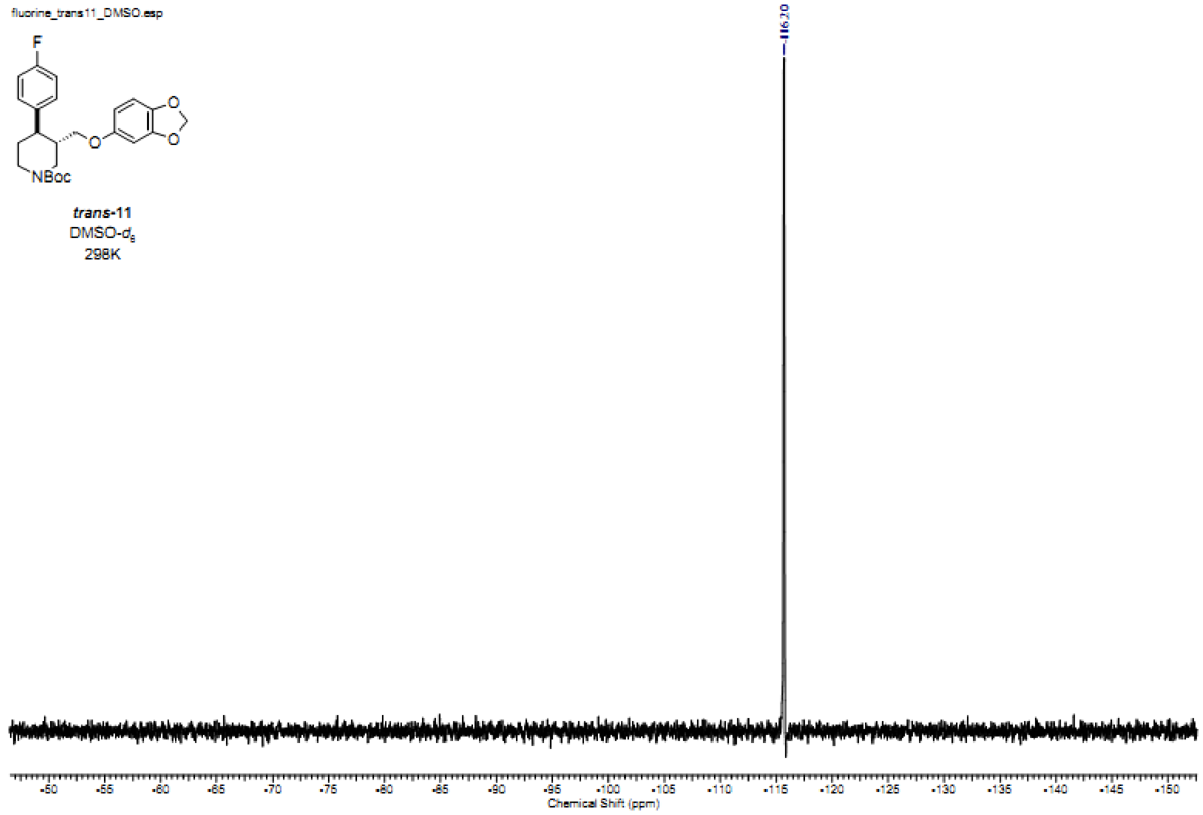
fluorine_trans11_DMSO.esp
F
O
O
O
NBoc
trans-11
DMSO-d_6
298K
-116.20
-50 -55 -60 -65 -70 -75 -80 -85 -90 -95 -100 -105 -110 -115 -120 -125 -130 -135 -140 -145 -150
Chemical Shift (ppm)

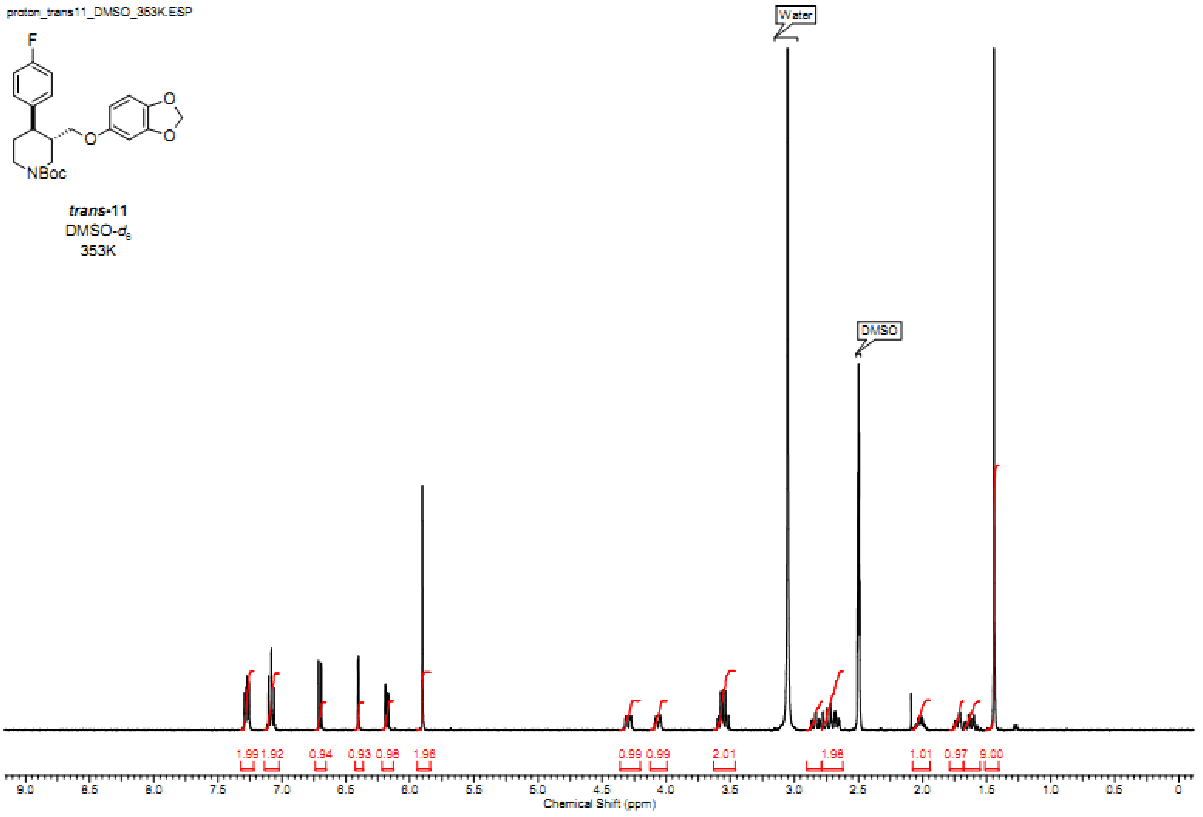
proton_trans11_DMSO_353K.ESP
trans-11
DMSO-d_6
353K
Water
DMSO
Chemical Shift (ppm)

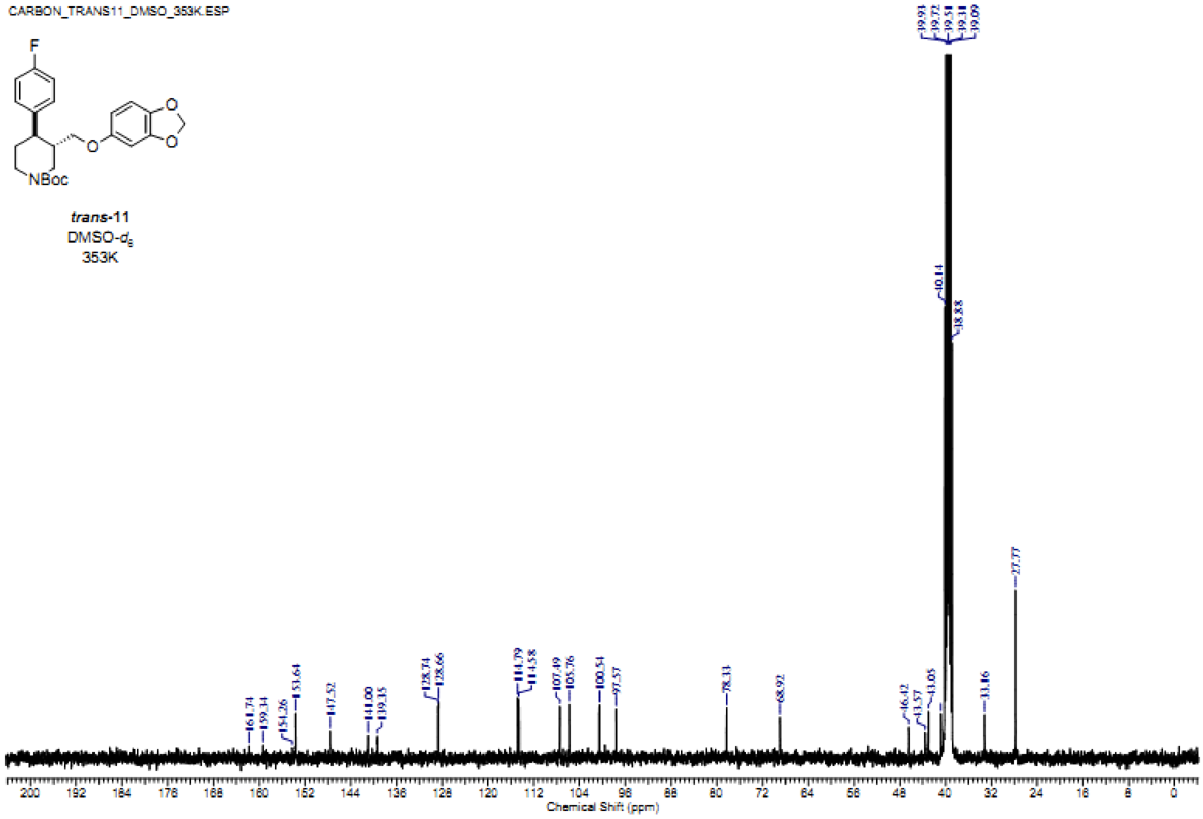
CARBON_TRANS11_DMSO_353K.ESP
trans-11
DMSO-d_6
353K
Chemical Shift (ppm)

Cis diastereoisomer degrades rapidly (NMR ran on a non-dry sample before degradation)

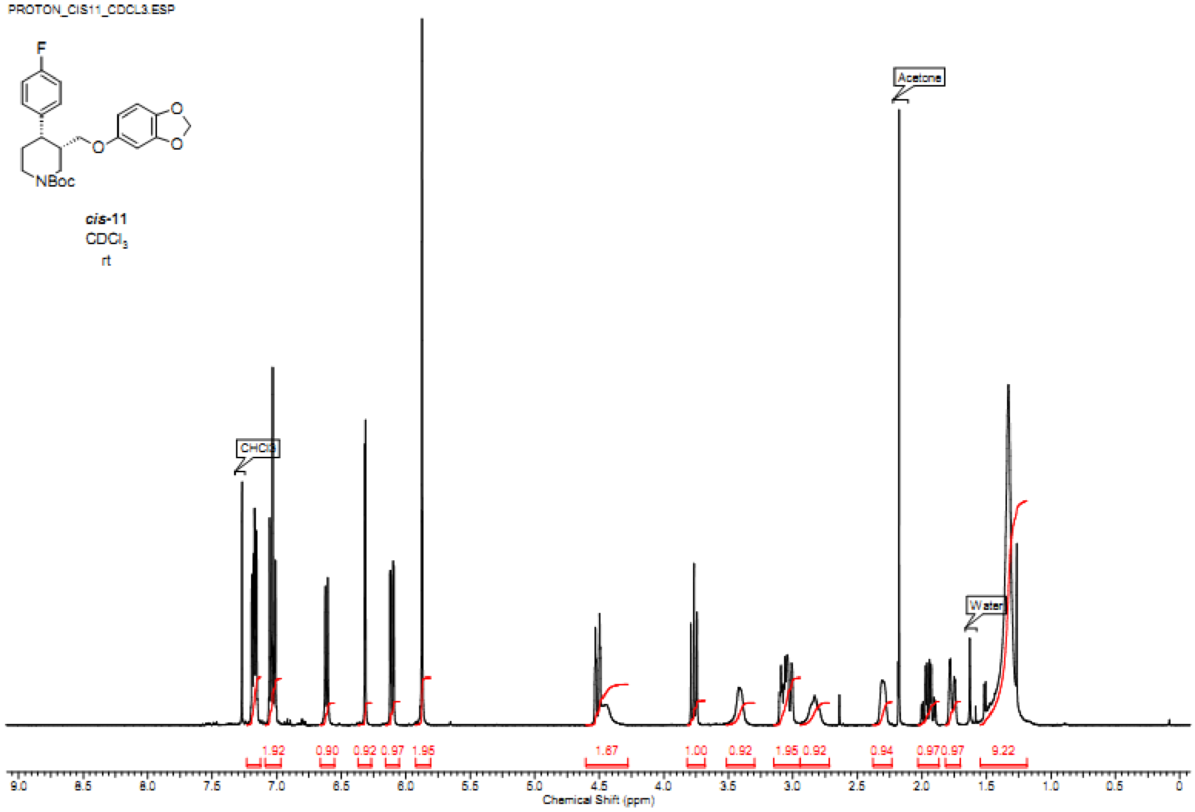

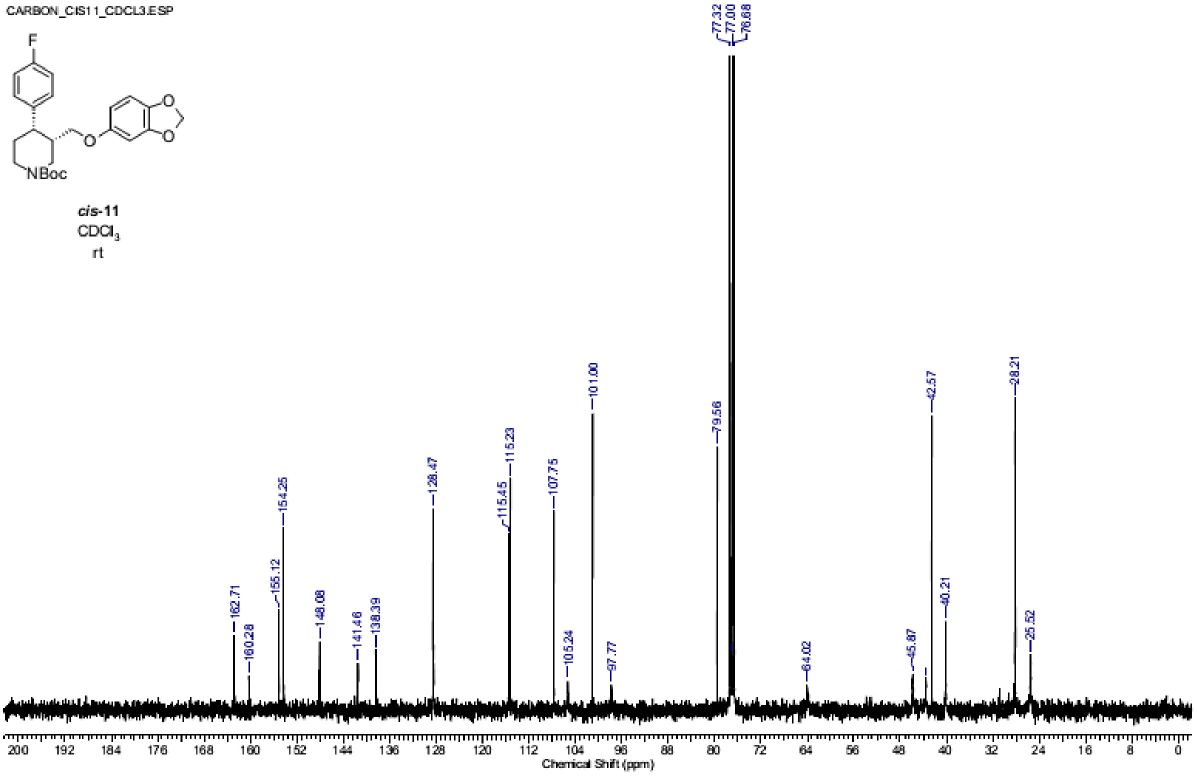

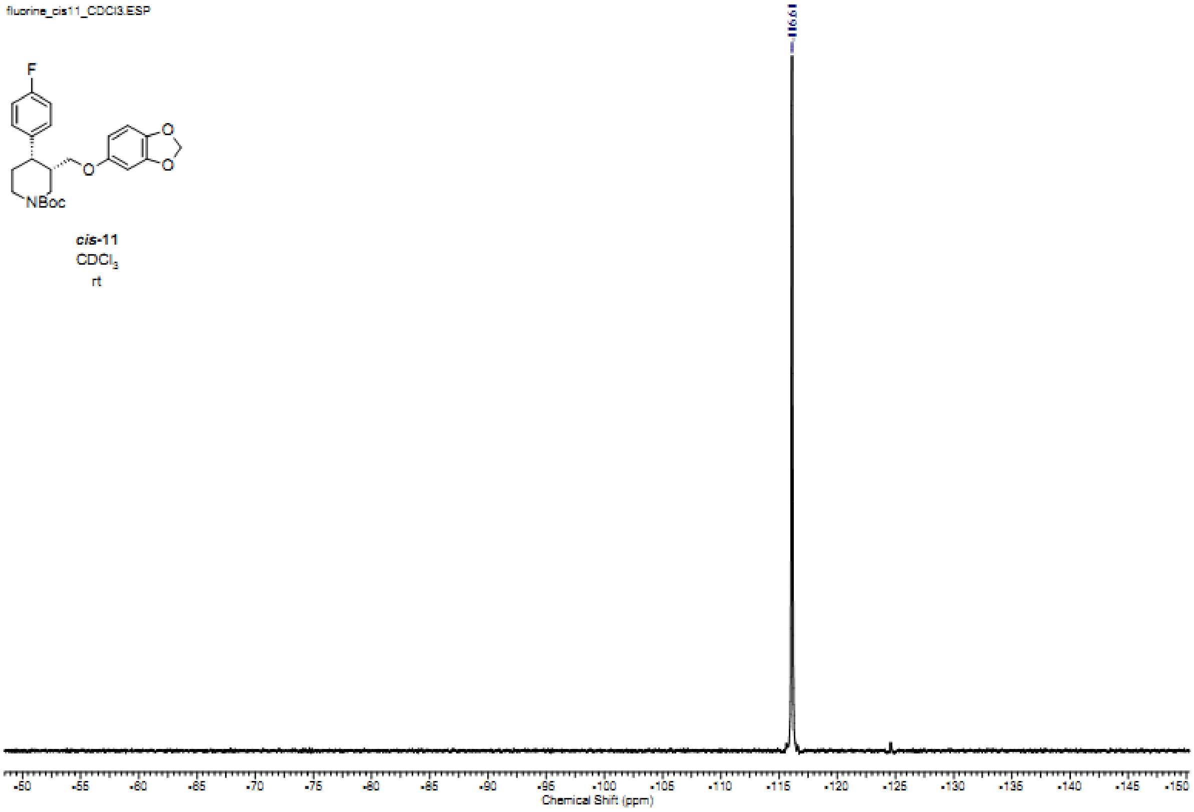
fluorine_cis11_CDCl3.ESP
F
O
O
O
NBoc
cis-11
CDCl3
rt
-50 -55 -60 -65 -70 -75 -80 -85 -90 -95 -100 -105 -110 -115 -120 -125 -130 -135 -140 -145 -150
Chemical Shift (ppm)

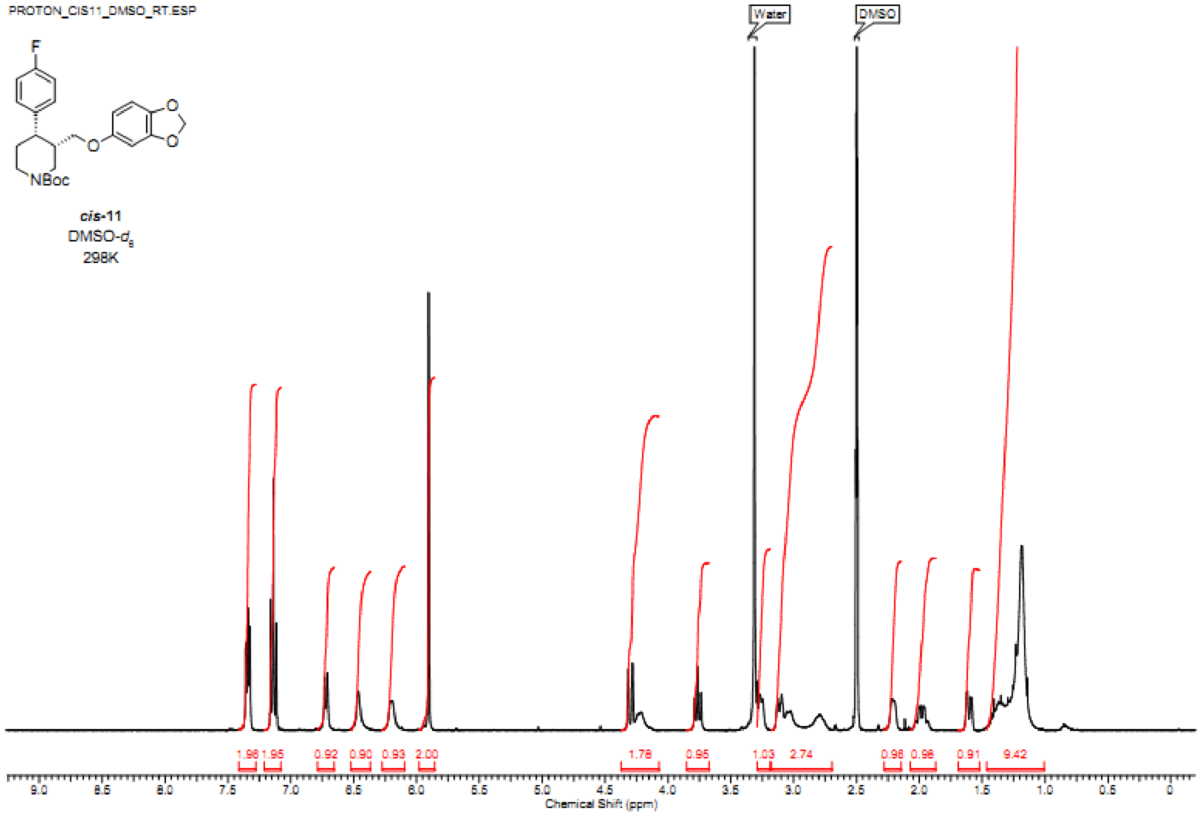
PROTON_CIS11_DMSO_RT.ESP
F
O
O
O
NBoc
cis-11
DMSO-d6
298K
Water
DMSO
1.96 1.95 0.92 0.90 0.93 2.00 1.78 0.95 1.03 2.74 0.96 0.96 0.91 9.42
9.0 8.5 8.0 7.5 7.0 6.5 6.0 5.5 5.0 4.5 4.0 3.5 3.0 2.5 2.0 1.5 1.0 0.5 0
Chemical Shift (ppm)

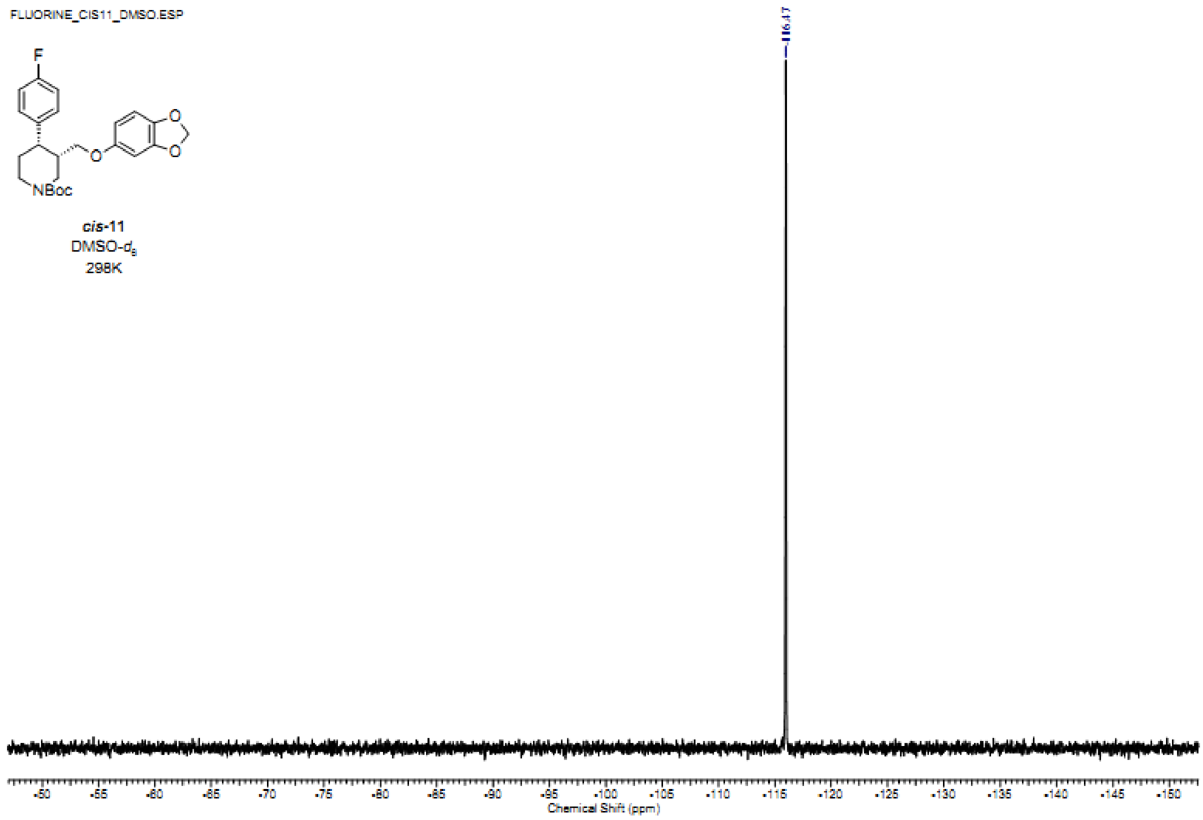
FLUORINE_CIS11_DMSO.ESP
F
O
O
O
NBoc
cis-11
DMSO-d6
298K
-116.47
-50 -55 -60 -65 -70 -75 -80 -85 -90 -95 -100 -105 -110 -115 -120 -125 -130 -135 -140 -145 -150
Chemical Shift (ppm)

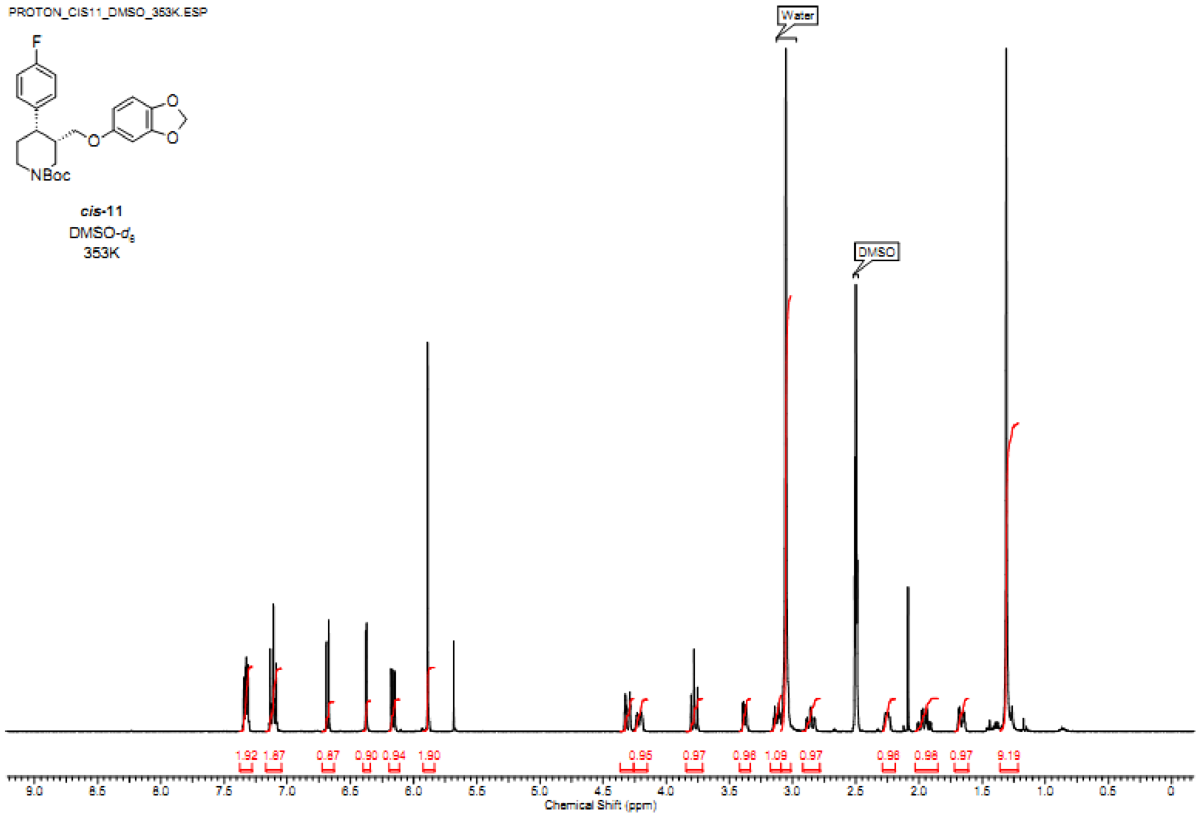
PROTON_CIS11_DMSO_353K.ESP
F
O
O
O
NBoc
cis-11
DMSO-d6
353K
Water
DMSO
1.92 1.87 0.87 0.90 0.94 1.90 0.95 0.97 0.98 1.09 0.97 0.96 0.98 0.97 9.19
9.0 8.5 8.0 7.5 7.0 6.5 6.0 5.5 5.0 4.5 4.0 3.5 3.0 2.5 2.0 1.5 1.0 0.5 0
Chemical Shift (ppm)

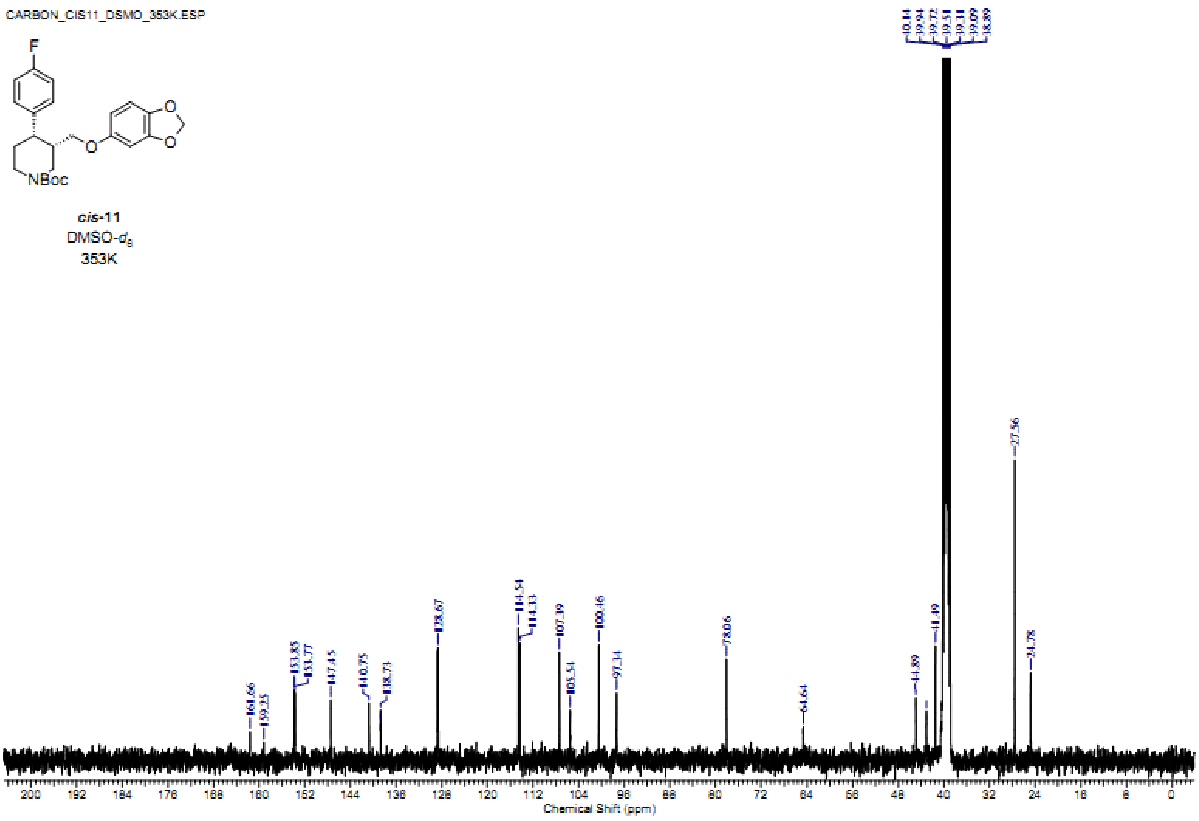

9. (±)-Paroxetine hydrochloride salt (5.HCl)

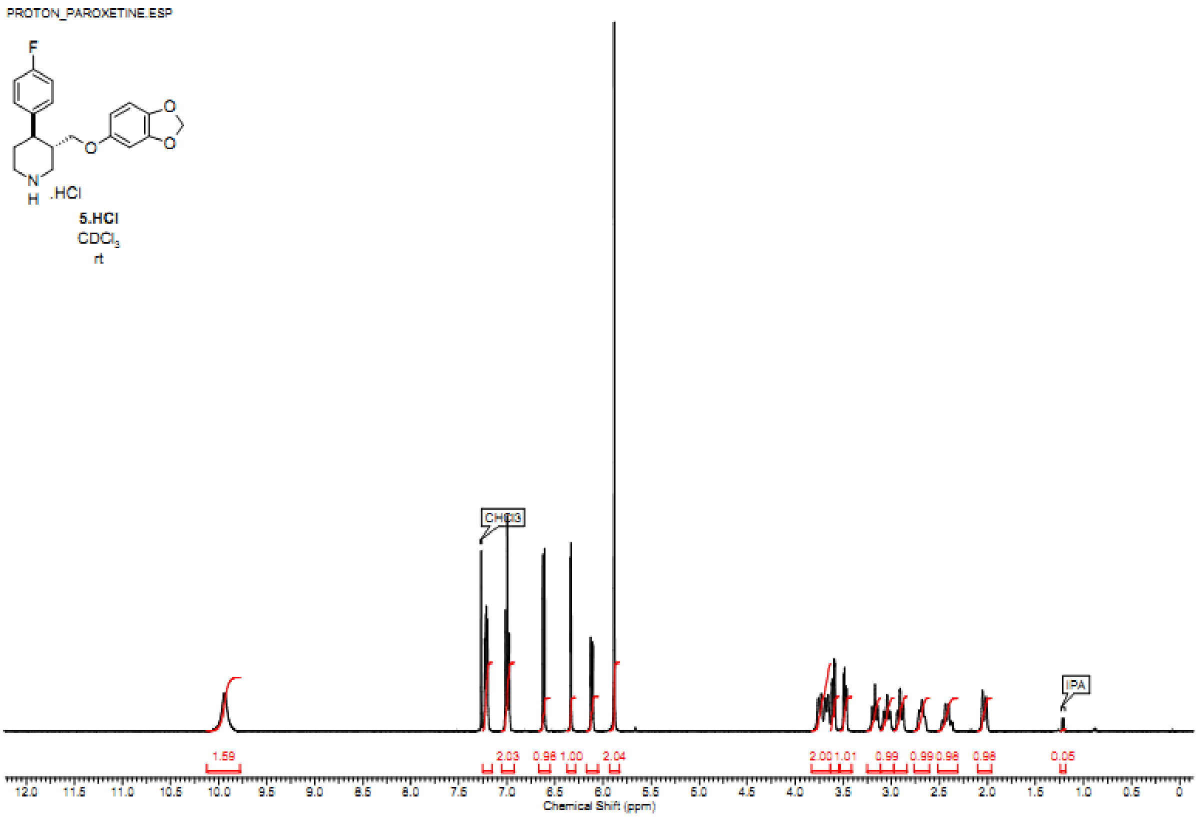

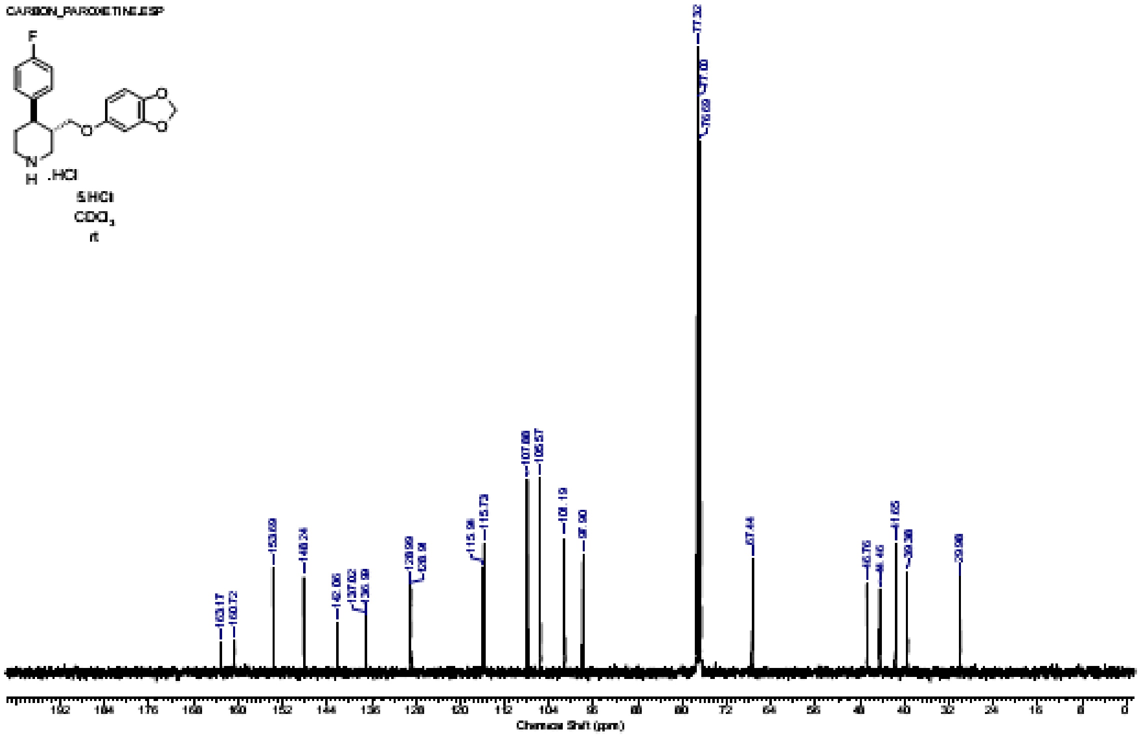
CARBON_PAROXETINE.ESP
F
O
O
O
N
H
.HCl
5.HCl
CDCl3
rt
77.32
77.00
76.69
163.17
160.72
153.69
148.24
142.06
137.02
136.99
128.99
128.91
115.98
115.73
107.89
105.57
101.19
97.90
67.44
45.76
44.45
41.65
39.39
29.98
Chemical Shift (ppm)

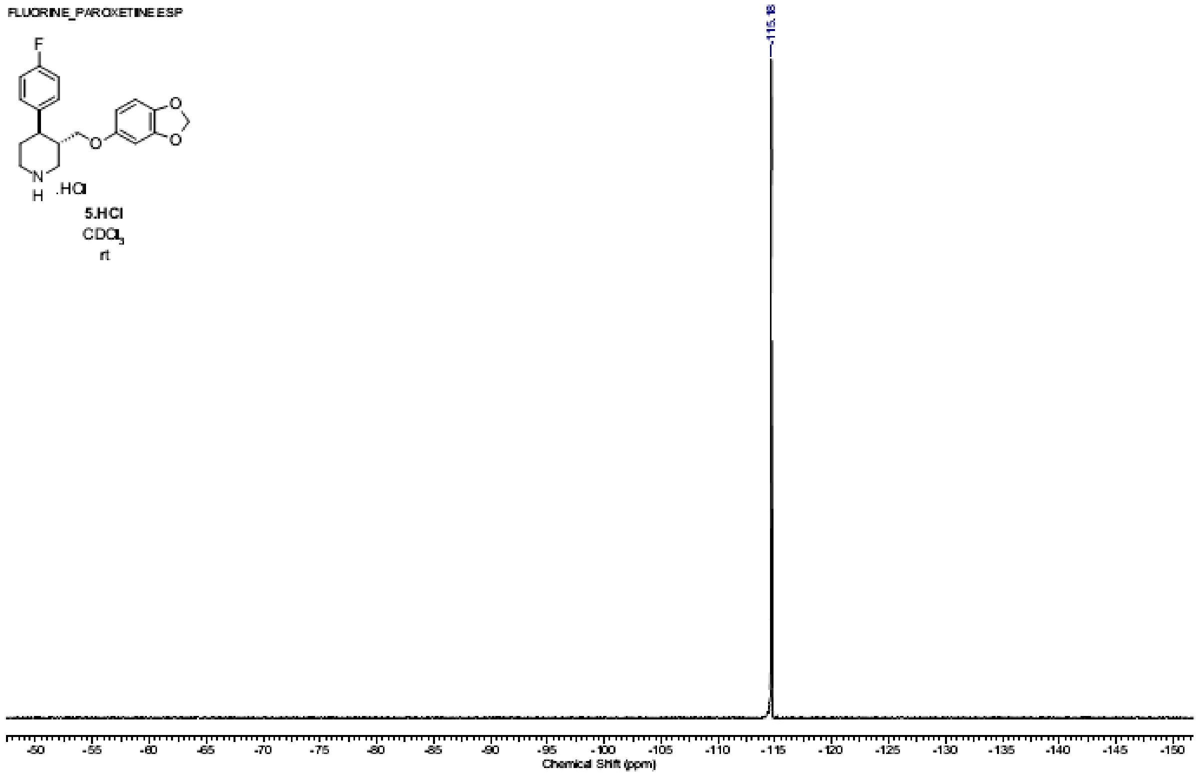
FLUORINE_PAROXETINE.ESP
F
O
O
O
N
H
.HCl
5.HCl
CDCl3
rt
-115.18
Chemical Shift (ppm)

10. 1-bromo-2-[3,4-(methylenedioxy)phenoxymethyl]-4-(tert-butyl)cyclohexane (*cis*-13)

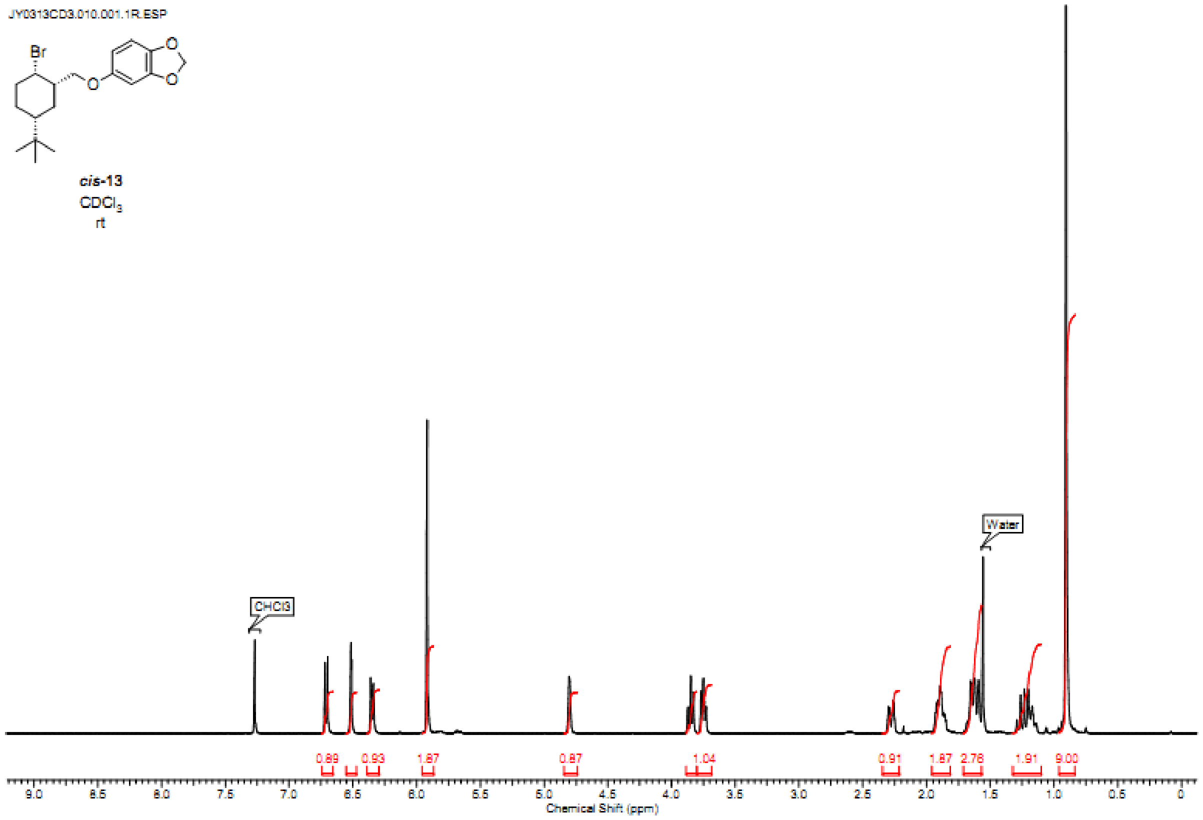

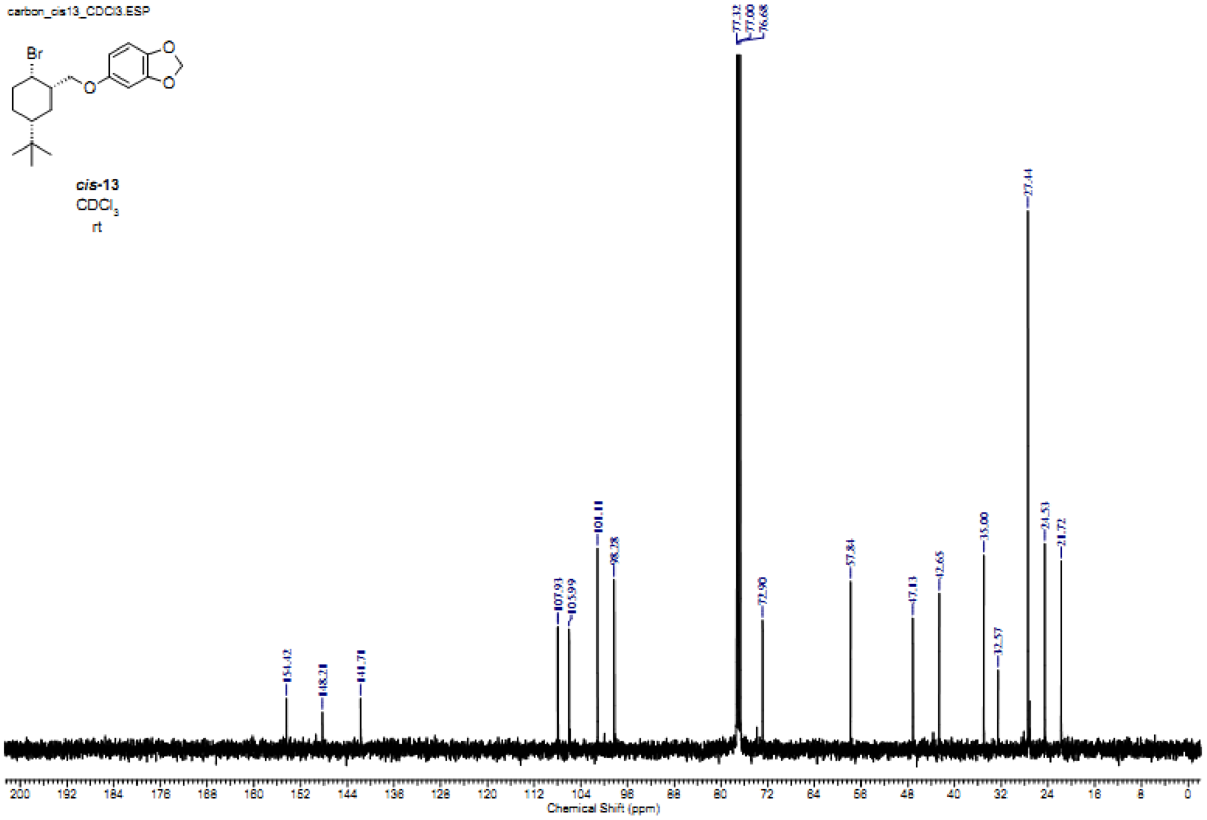

11. 1-(4-fluorophenyl)-2-[3,4-(methylenedioxy)phenoxymethyl]-4-(tert-butyl)cyclohexane (*trans*-14)

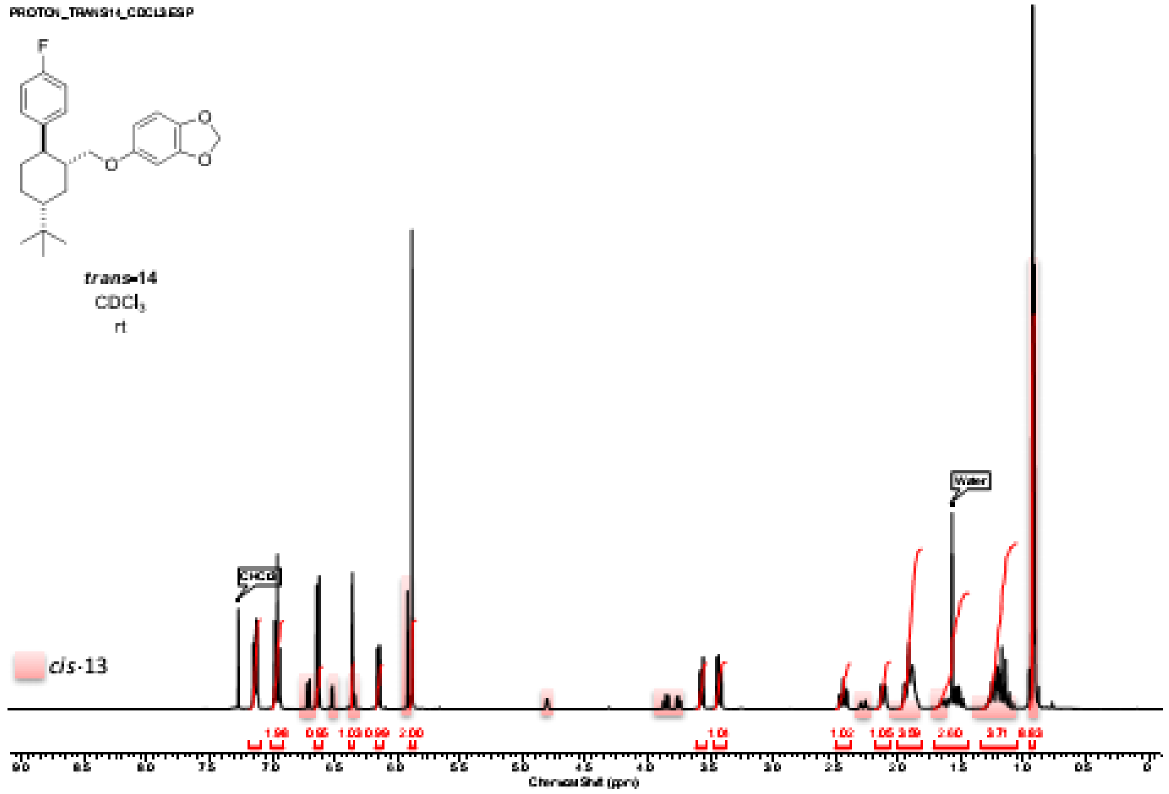

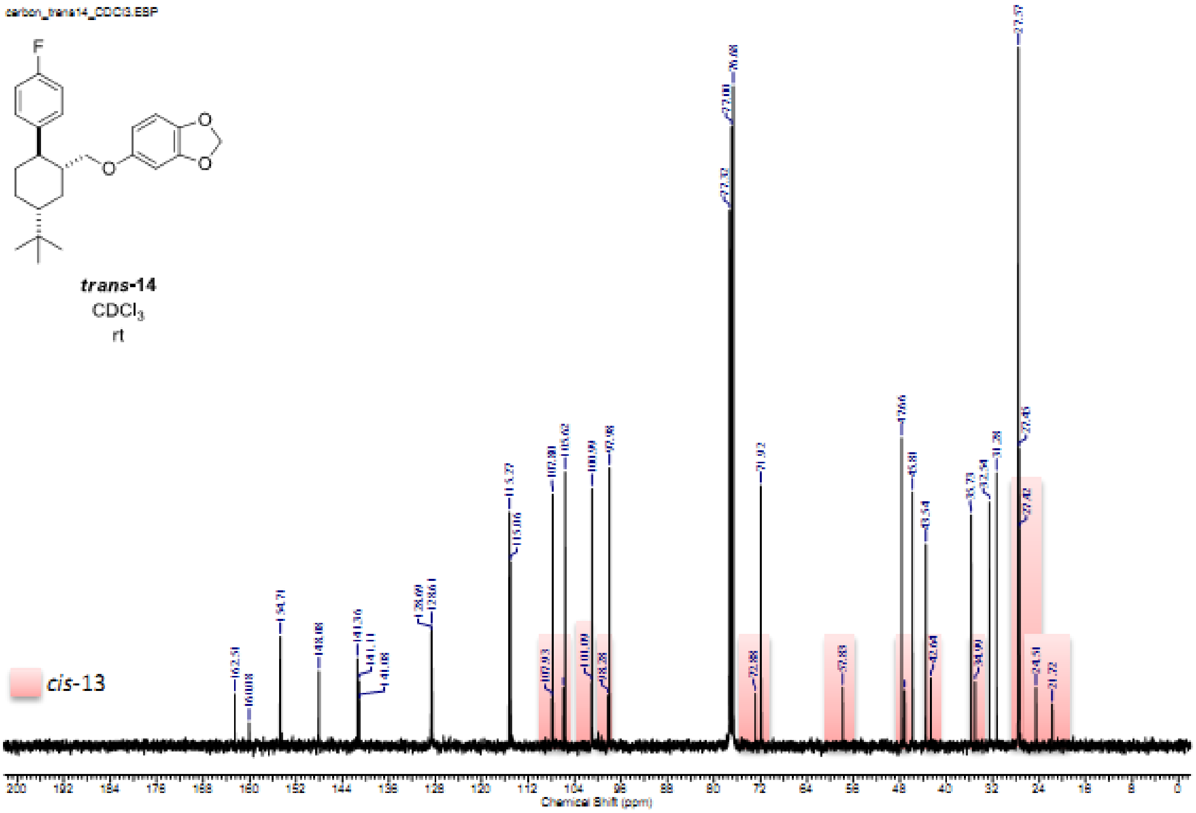

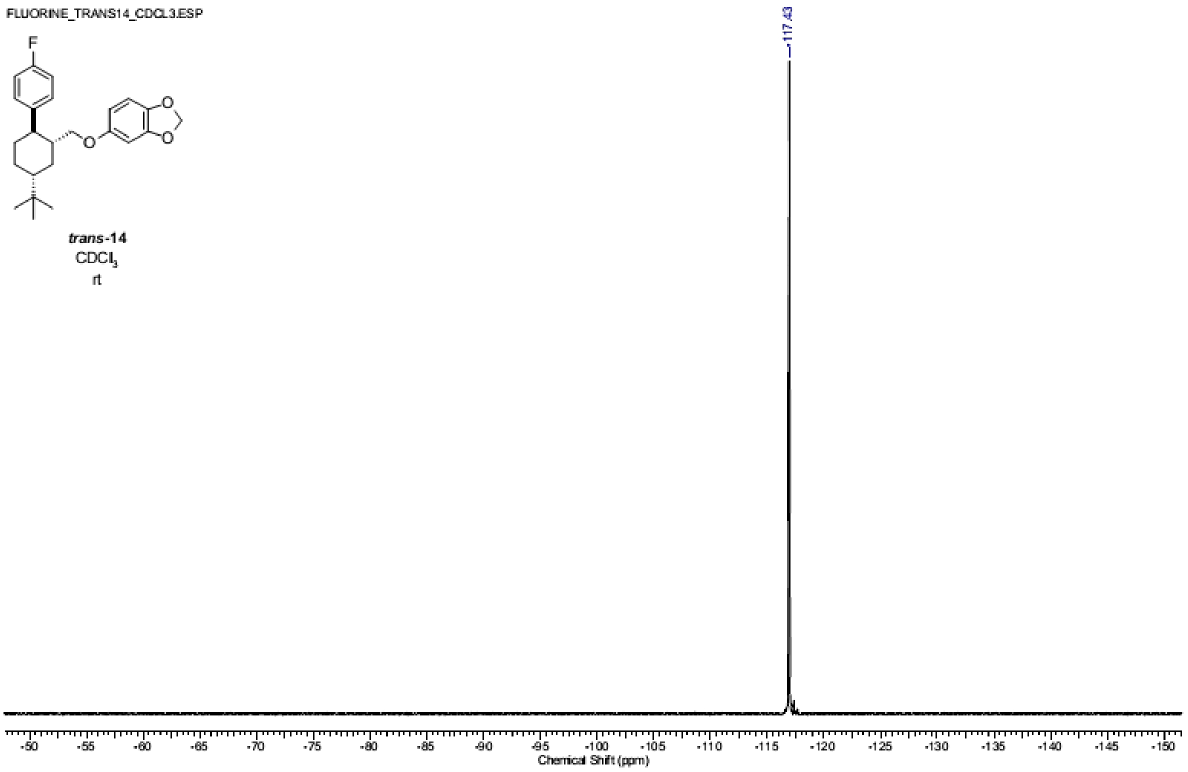

References

[1] J. Lázár, G. Bernáth, *Heterocycl. Chem.* **1990**, *27*, 1885-1892.
[2] K. Soai, H. Oyamada, *Synthesis* **1984**, *1984*, 605-607.
[3] M. Amat, J. Bosch, J. Hidalgo, M. Canto, M. Perez, N. Llor, E. Molins, C. Miravitlles, M. Orozco, J. Luque, *J. Org. Chem.* **2000**, *65*, 3074-3084.
[4] J. F. Bower, T. Riis-Johannessen, P. Szeto, A.J. Whitehead, T. Gallagher, *Chem. Commun.* **2007**, 728-730.
[5] M. Lachia, F. Dénès, F. Beaufils, P. Renaud, *Org. Lett.* **2005**, *7*, 4103-4106.
